# Pathogenic role of acyl coenzyme A binding protein (ACBP) in Cushing’s syndrome

**DOI:** 10.1038/s42255-024-01170-0

**Published:** 2024-11-22

**Authors:** Hui Pan, Ai-Ling Tian, Hui Chen, Yifan Xia, Allan Sauvat, Stephanie Moriceau, Flavia Lambertucci, Omar Motiño, Liwei Zhao, Peng Liu, Misha Mao, Sijing Li, Shuai Zhang, Adrien Joseph, Sylvère Durand, Fanny Aprahamian, Zeyu Luo, Yang Ou, Zhe Shen, Enfu Xue, Yuhong Pan, Vincent Carbonnier, Gautier Stoll, Sabrina Forveille, Marion Leduc, Giulia Cerrato, Alexandra Cerone, Maria Chiara Maiuri, Frederic Castinetti, Thierry Brue, Hongsheng Wang, Yuting Ma, Isabelle Martins, Oliver Kepp, Guido Kroemer

**Affiliations:** 1 Metabolomics and Cell Biology Platforms, UMS AMMICa, Gustave Roussy Institut, Villejuif, France; 2 Centre de Recherche des Cordeliers, Equipe labellisée par la Ligue contre le cancer, INSERM U1138, Université Paris Cité, Sorbonne Université, Paris, France; 3 Faculté de Médecine, Université Paris-Saclay, Paris, France; 4 National Key Laboratory of Immunity and Inflammation, Suzhou Institute of Systems Medicine, Chinese Academy of Medical Sciences and Peking Union Medical College, Suzhou, China; 5 Institut Imagine, Platform for Neurobehavioral and Metabolism, Structure Federative de Recherche Necker, 26 INSERM US24/CNRS UAR, Paris, France; 6 Department of Surgical Oncology, Sir Run Run Shaw Hospital, Zhejiang University, Hangzhou, China; 7 Department of Respiratory and Critical Care Medicine, Union Hospital, Tongji Medical College, Huazhong University of Science and Technology, Wuhan, China; 8 Service de Réanimation Medicale, Hôpital Saint-Louis, Assistance Publique Hôpitaux de Paris, Paris, France; 9 Department of Orthopedics, West China Hospital/West China School of Medicine, Sichuan University, Chengdu, China; 10 Center for Tumor Diagnosis and Therapy, Jinshan Hospital, Fudan University, Shanghai, China; 11 Research Center of Avian Disease, College of Veterinary Medicine, Sichuan Agricultural University, Chengdu, China; 12 Assistance Publique Hôpitaux de Marseille, Department of Endocrinology, La Conception Hospital, Marseille, France; 13 Department of Mycobacterium, Jiangsu Key Laboratory of Molecular Biology for Skin Diseases and STIs, Institute of Dermatology and Hospital for Skin Diseases, Chinese Academy of Medical Sciences and Peking Union Medical College, Nanjing, China; 14 Center for Global Health, School of Public Health, Nanjing Medical University, Nanjing, China; 15 Institut du Cancer Paris CARPEM, Department of Biology, Hôpital Européen Georges Pompidou, AP-HP, Paris, France

**Keywords:** Steroid hormones, Metabolomics, Metabolism

## Abstract

Cushing’s syndrome is caused by an elevation of endogenous or pharmacologically administered glucocorticoids. Acyl coenzyme A binding protein (ACBP, encoded by the gene diazepam binding inhibitor, *Dbi*) stimulates food intake and lipo-anabolic reactions. Here we found that plasma ACBP/DBI concentrations were elevated in patients and mice with Cushing’s syndrome. We used several methods for ACBP/DBI inhibition in mice, namely, (1) induction of ACBP/DBI autoantibodies, (2) injection of a neutralizing monoclonal antibody, (3) body-wide or hepatocyte-specific knockout of the *Dbi* gene, (4) mutation of the ACBP/DBI receptor *Gabrg2* and (5) injections of triiodothyronine or (6) the thyroid hormone receptor-β agonist resmetirom to block *Dbi* transcription. These six approaches abolished manifestations of Cushing’s syndrome such as increased food intake, weight gain, excessive adiposity, liver damage, hypertriglyceridaemia and type 2 diabetes. In conclusion, it appears that ACBP/DBI constitutes an actionable target that is causally involved in the development of Cushing’s syndrome.

## Abstract (web summary)

The authors highlight the role of acyl coenzyme A binding protein (encoded by *DBI*) in Cushing’s syndrome by using six different inhibition methods and mapping the physiological effects.

## Main

Cushing’s syndrome results from the chronic hyperactivation of glucocorticoid receptors, usually for several months, and is marked by a characteristic phenotype that includes a round ‘moon face’ with capillary vasodilatation, skin acne, face hirsutism, cranial alopecia, central obesity, lipodystrophy with a ‘buffalo hump’ at the back of the neck, profuse striae, skin atrophy, sarcopenia and osteoporosis^1,2^. In addition, Cushing’s syndrome is accompanied by a metabolic syndrome including dyslipidaemia (mostly triglyceridaemia), insulin resistance, hyperglycaemia and arterial hypertension, sometimes culminating in death due to atherosclerotic disease, cardiac failure or thromboembolism^3,4^. Furthermore, immunosuppression may increase the susceptibility to severe infections^5^. Endogenous Cushing’s syndrome, which is often diagnosed with a significant delay (mean delay to diagnosis: 34 months) occurs due to the excessive production of endogenous glucocorticoids, usually as the result of tumours, mostly pituitary adenomas, that produce adrenocorticotropic hormone (ACTH), then overstimulating the adrenal glands to produce cortisol^4^. Iatrogenic Cushing’s syndrome results from long-term treatments with synthetic glucocorticoids, as this may be necessary for the control of chronic asthma, rheumatoid arthritis, lupus, sarcoidosis and other severe inflammatory conditions^1,2^.

Acyl coenzyme A binding protein (ACBP) is encoded by the gene diazepam binding inhibitor (*Dbi*). This dual designation, ACBP/DBI, reflects the two roles of the protein^6^. First, ACBP/DBI is an intracellular protein interacting with activated fatty acids as well as with other lipids to facilitate their transport between organelles. Thus, ACBP/DBI acts as a regulator of long-chain acyl-CoA and ceramide metabolism, and as a facilitator of mitochondrial transport of cholesterol for steroid synthesis^7–9^. Second, ACBP/DBI can be found in the extracellular space where it acts as a positive allosteric modulator on a specific subtype of γ-aminobutyric acid (GABA) A receptors (GABA_A_R) containing the diazepam-binding subunit GABRG2^10,11^. In addition, ACBP/DBI and its fragmentation products (such as octadecaneuropeptide) can interact with a G-protein-coupled receptor in the central nervous system^12^. ACBP/DBI is a leaderless peptide that cannot be secreted by conventional (Golgi-dependent) protein secretion but rather leaves cells through an autophagy-associated pathway^13,14^. ACBP/DBI is phylogenetically conserved throughout the eukaryotic radiation, and this mode of unconventional secretion is maintained in unicellular fungi and mammalian cells^15,16^. ACBP/DBI plays a major role in adapting cells and organisms to nutrient stress. Starvation causes an acute autophagy-mediated surge in extracellular ACBP/DBI protein, thus activating an adaptive response to nutrient stress (such as sporulation in fungi, pharyngeal pumping in nematodes and feeding behaviour in mice)^14,17,18^. Stimulation of feeding behaviour by ACBP/DBI released into the systemic circulation is mediated by indirect effects on appetite control centres in the brain because ACBP/DBI does not cross the blood–brain barrier^18^. In addition, extracellular ACBP/DBI acts as an extracellular checkpoint of autophagy^6,19^, which constitutes a mechanism how cells recycle their cytoplasm, adapt to stress and rejuvenate themselves^20,21^. Long-term elevations of ACBP/DBI are observed in old age, obesity, metabolic syndrome, chronic inflammation and kidney failure^6,14,22–26^. They are probably maladaptive because knockout of ACBP/DBI prolongs lifespan in model organisms (yeast and nematodes)^27,28^, prevents accelerated cardiac ageing induced by anthracyclines in mice^6^ and protects several organs (that is, brain, heart, liver and lung) against acute damage by reducing cell death, inflammation and fibrosis^29^.

Intrigued by the possibility that ACBP/DBI is (one of) the phylogenetically most ancient peptide hormone(s)^29^, we became interested in its (patho)physiological roles. Here, we used an in vitro screen to identify other neuroendocrine factors that regulate ACBP/DBI. We found that glucocorticoid receptor activation stimulates the release of ACBP/DBI from cultured cells and increases plasma ACBP/DBI concentrations in mice. Moreover, thyroid hormone transcriptionally downregulates ACBP/DBI. In a mouse model of iatrogenic Cushing’s syndrome, knockout of the *Dbi* gene, mutation of *Gabrg2*, antibody-mediated neutralization of ACBP/DBI or transcriptional downregulation of ACBI/DBI by thyroid hormone all prevent the metabolic consequences of chronic glucocorticoid administration. Our observations suggest that important facets of Cushing’s syndrome are mediated by a surge in extracellular ACBP/DBI.

## Results

### Corticosteroids and thyroid hormones modulate ACBP

**Fig. 1:**
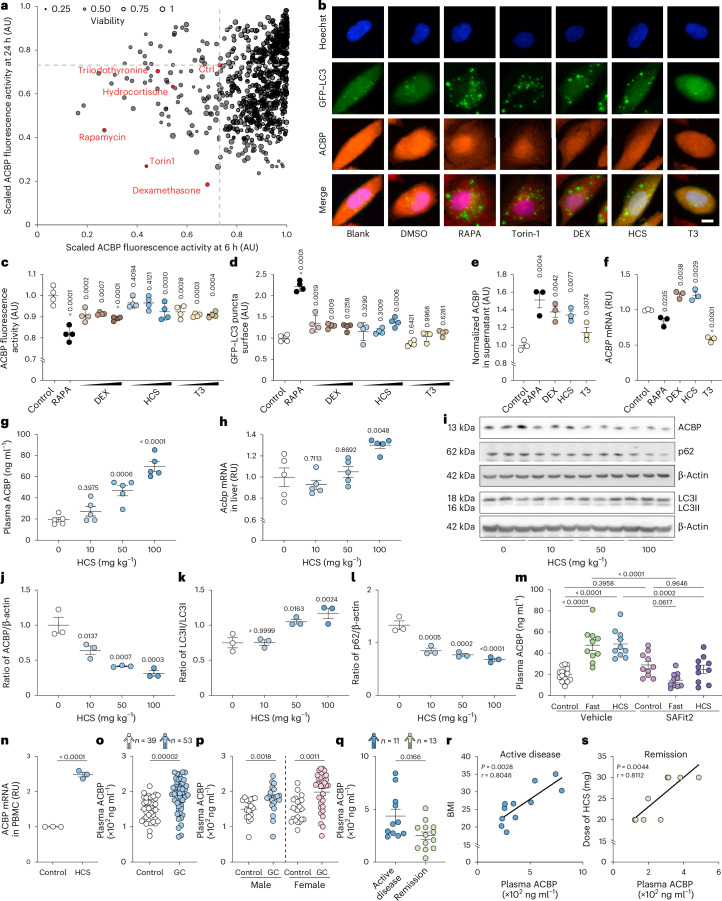
Identification of corticosteroids and thyroid hormone as ACBP/DBI modulators. **a**,**b**, H4 cells expressing GFP–LC3 were treated with agonists and antagonists of neurotransmitter and hormone receptors (5 µM). ACBP was assessed by immunohistochemistry. Scaled ACBP fluorescence intensity and viability are depicted (**a**) together with representative images (**b**). Rapamycin (RAPA; 10 µM), Torin-1 (0.3 µM) and DMSO (control) were used as controls. Scale bar, 5 µm. **c**,**d**, The plots show ACBP fluorescence (**c**) and GFP–LC3 puncta (**d**) (16 h; 0.01, 0.05 and 0.1 µM DEX, HCS and T3 in dialysed foetal bovine serum (AU, arbitrary units; mean ± s.d.). **e**,**f**, H4 cells were treated with DEX, HCS and T3 (0.1 µM) for 16 h. ACBP release was assessed by ELISA (**e**), and *ACBP* mRNA levels were measured by qRT–PCR (RU, relative units) (**f**). **g**,**h**, Female C57BL/6J (8-week-old) mice (*n* = 5 per group) were treated with HCS (10, 50 and 100 mg kg^−1^; i.p.) for 24 h. Plasma ACBP was measured by ELISA (**g**) and hepatic *Acbp* mRNA was assessed by qRT–PCR (**h**). **i**–**l**, ACBP abundance, LC3 conversion and p62 degradation were measured in liver tissue (representative blots in (**i** and quantifications of the ratios of the indicated proteins in **j**–**l**) (*n* = 3 per group)). **m**, Mice were fasted or received HCS (100 mg kg^−1^; i.p.) for 24 h combined with SAFit2 (40 mg kg^−1^; i.p.) or vehicle, and plasma ACBP was measured by ELISA (*n* = 15, 10, 10, 10, 10 and 10 mice per group). **n**, PBMCs were treated with HCS (0.5 μM) for 16–18 h, and *ACBP* mRNA was assessed by qRT–PCR. **o**,**p**, Plasma ACBP levels were measured in dermatology patients receiving (*n* = 53) or not (*n* = 39) glucocorticoid treatment (**o**), and data were grouped by sex (**p**). The Wilcoxon test was used and *P* values were calculated according to a multivariate model including age and BMI. **q**, Plasma ACBP was measured in ACTH-dependent patients with Cushing’s syndrome with hypercortisolaemia (*n* = 11) or in remission (*n* = 13); an unpaired *t*-test was used for analysis. **r**,**s**, Plasma ACBP was plotted against BMI in the hypercortisolaemia group (*n* = 11) (**r**) and against daily HCS dose (in the case of corticotroph deficiency) in the remission group (*n* = 10) (**s**). The dot plots depict mean ± s.e.m., if not otherwise indicated. One-way ANOVA with Dunnet correction (**c**–**h** and **j**–**m**), Mann–Whitney test (two tailed) (**o** and **p**), unpaired *t*-test (two tailed) (**q** and **n**) and Pearson correlation (**r** and **s**) were used for statistical analysis (*P* values are indicated). Source data

**Extended Data Fig. 1:**
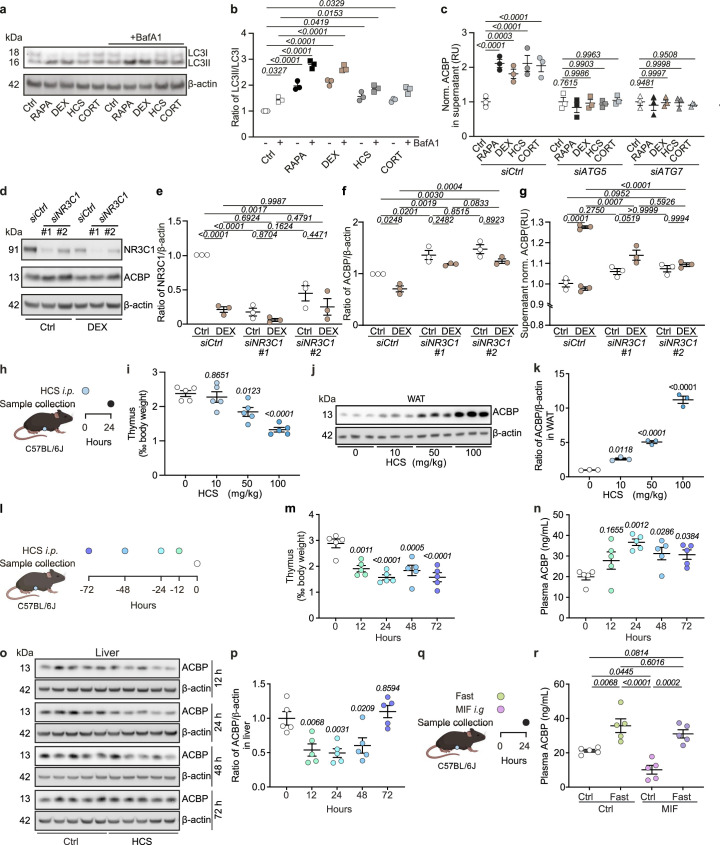
Identification of corticosteroids and thyroid hormones as ACBP/DBI modulators. (**a-c**) H4 cells were treated with dexamethasone (DEX), hydrocortisone (HCS), or corticosterone (CORT) (1 μM) for 6 h with/without bafilomycin A1 (BafA1, 100 nM, final 2 h). Rapamycin (RAPA, 10 μM) was used as positive control. Representative immunoblots of LC3 conversion are shown in (**a**) and quantifications in (**b**) (*n=3/group*). β-actin was used as a loading control. (**c**) Cells were transfected with siRNA targeting *ATG5* and *ATG7* or control siRNA (*siCtrl*) and then treated as indicated (*n=3/group;* RU, relative units). (**d-g**) Cells were transfected with siRNA targeting *NR3C1* or *siCtrl*. ACBP release was assessed by ELISA. (**d**) Representative immunoblots show NR3C1 and ACBP levels in *NR3C1* knockdowns with/without DEX (1 μM, 24 h). (**e-f**) Scatter plot shows the ratio of NR3C1/β-actin (**e**) and ACBP/β-actin (**f**) with the indicated treatments. (**g**) ACBP release from cells with the indicated treatments is shown in (**d**) (RU; *n=3*; One-way ANOVA; *P-*values are indicated). All dot plots depict means ± SEM. (**h**) Scheme shows the schedule of hydrocortisone (HCS, 10, 50, 100 mg/kg, *i.p*.) administration to female C57BL/6J mice (*n=5/group*). (**i**) Percentage of mouse thymus relative to body weight following HCS treatment at the specified doses (*n=5/group*). (**j**, **k**) Representative immunoblot (**j**) showing ACBP levels in WAT treated with HCS at different dose (*n=5/group*). Scatter plot (**k**) shows the ratio of ACBP/β-actin (**k**). (**l**) Scheme shows the schedule of hydrocortisone (HCS, 100 mg/kg, *i.p*.) administration to female C57BL/6J mice for the indicated duration. (**m**) Percentage of mouse thymus relative to body weight following HCS treatment at various time points (*n=5/time point*). (**n**) Plasma ACBP level under HCS treatments at various time points (*n=5/time point*). (**o**) Representative immunoblots show hepatic ACBP upon treatment with HCS at various time points (*n=5/group*). Scatter plots show the ratio of ACBP/β-actin (**p**). (**h**) Scheme shows the schedule of mifepristone (Mif, 120 mg/kg, *i.g*.) administration and fasting in female C57BL/6J mice for 24 h. (**q**, **r**) Scheme of the experiment (**q**) and plasma ACBP levels (**r**) upon Mif treatment and fasting for 24 h. One-way ANOVA with Dunnett correction was used for statistical analysis (**i,k,m,n,p,r**). Data in **b,c,e,f,g** were analyzed using two-way ANOVA with Tukey correction. (*P-*values are indicated). All dot plots depict means ± SEM. Source data

Driven by the theoretical consideration that neuroendocrine factors are usually embedded in regulatory circuitries involving other neuroendocrine factors^30,31^, we designed a screen in which we evaluated the impact of 710 distinct agonists and antagonists of neurotransmitter and hormone receptors on ACBP/DBI expression by H4 human neuroglioma cells. For this, H4 cells expressing an autophagy biosensor (microtubule-associated protein 1A/1B-light chain fused to green fluorescent protein, GFP–LC3) were cultured for 6 or 24 h in the absence or presence of 5 µM of each of the agents assembled in a custom arrayed compound library (Supplementary Table 1). The cells were then subjected to the immunofluorescence detection of ACBP/DBI^32^. Automated fluorescence microscopy followed by image analyses confirmed that autophagy inducers used as positive controls (rapamycin and torin-1) reduced the fluorescent signal corresponding to ACBP/DBI while they caused the aggregation of GFP–LC3 in cytoplasmic dots (Fig. 1a–d). Glucocorticoids exemplified by hydrocortisone (HCS, the natural human hormone) and dexamethasone (DEX, a synthetic analogue with higher potency than HCS) induced a similar pattern of ACBP/DBI reduction and GFP–LC3 puncta (Fig. 1a–d). In contrast, the thyroid hormone 3,3′,5-triiodo-l-thyronine (triiodothyronine, T3) attenuated ACBP/DBI expression without induction of GFP–LC3 puncta (Fig. 1a–d). These effects, also detected in the HepG2 cell line, were dose dependent (Supplementary Fig. 1) and were accompanied by the secretion of ACBP/DBI into culture supernatants for rapamycin, DEX and HCS, but not for T3 (Fig. 1e). Both HCS and DEX upregulated, whereas T3 downregulated, the messenger RNA coding for ACBP/DBI (Fig. 1f). HCS and DEX induced the autophagy-associated lipidation of LC3, giving rise to the electrophoretically more mobile LC3-II band, and this was found both in the absence and in the presence of bafilomycin A1 (BafA1), indicating that corticosteroids induce autophagic flux (Extended Data Fig. 1a,b). Moreover, ACBP secretion in response to glucocorticoids was inhibited by the knockdown of autophagy-related 5 (*ATG5*) and autophagy-related 7 (*ATG7*) (Extended Data Fig. 1c). The capacity of corticosteroids to attenuate intracellular ACBP/DBI expression and to increase secretion of ACBP/DBI into culture supernatants was inhibited by knockdown of the glucocorticoid receptor NR3C1 (Extended Data Fig. 1d–g). In mice, HCS administration induced dose- and time-dependent thymolysis (Extended Data Fig. 1h,i,l,m). This effect was accompanied by a significant increase in plasma ACBP/DBI concentrations (Fig. 1g and Extended Data Fig. 1n) and hepatic *ACBP*/*DBI* mRNA levels (Fig. 1h). Conversely, there was a decrease in liver ACBP/DBI protein (Fig. 1i,j and Extended Data Fig. 1o,p), contrasted by an increase in white adipose tissue (WAT) ACBP/DBI protein levels (Extended Data Fig. 1j,k). These changes were associated with signs of autophagy, including LC3 lipidation and depletion of SQSTM1/p62 (Fig. 1i,k,l).

**Extended Data Fig. 2:**
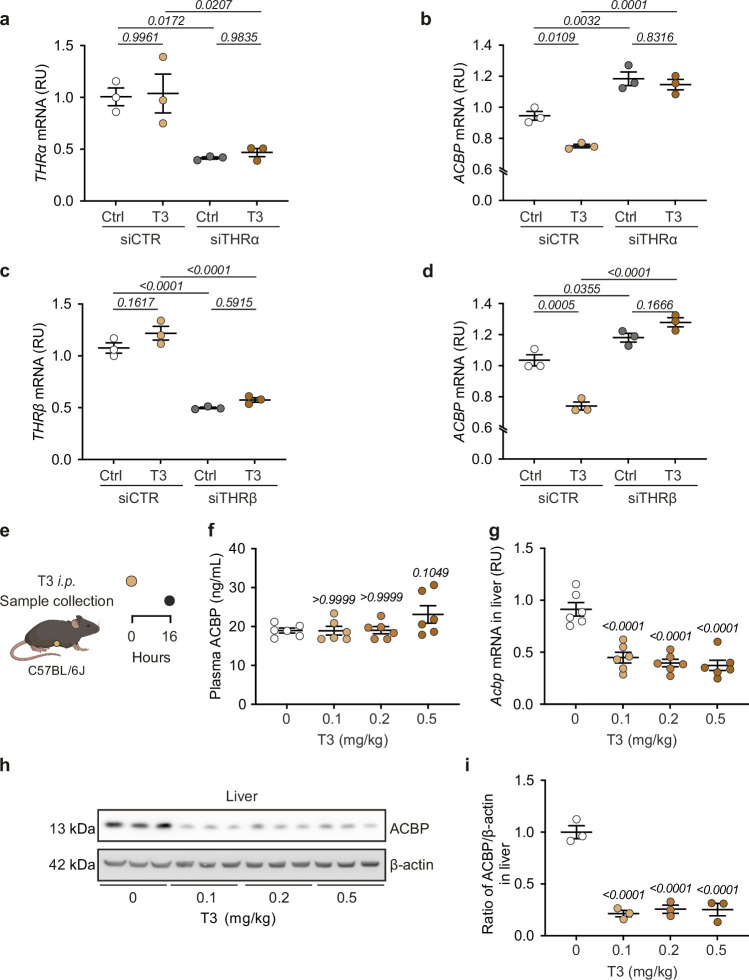
Effects of short-term triiodothyronine administration on ACBP/DBI expression. (**a-d**) Cells were treated with siRNA targeting the thyroid hormone receptor (*THR*) α and β genes or control siRNA (*siCtrl*) and then cultured in the presence of triiodothyronine (T3). Then cells were subjected to the quantification of the mRNA coding for THRα, THRβ or ACBP (*n=3/group*; RU, relative units). (**e**) Scheme of T3 administration to female C57BL/6 mice for 16 h. (**f**) Plasma ACBP was assessed by ELISA after T3 treatment at different doses (*n=6*/*group*). (**g**) Liver *Acbp* mRNA levels (*n=6/group;* RU, relative units) after T3 treatment. (**h**) Representative immunoblot of ACBP after T3 treatment (*n=3/group*). β-actin was used as a loading control. (**i**) Scatter plot showing the ACBP/β-actin ratio. One-way ANOVA with Tukey correction was used for statistical analysis (*P-*values are indicated). All dot plots indicate means ± SEM. Source data

Of note, the fasting and HCS-induced surge in plasma ACBP/DBI was abrogated by treatment with SAFit2 (Fig. 1m), which is an inhibitor of autophagy-dependent secretion^33^. All effects caused by HCS in vivo were mimicked by DEX and were counteracted by the glucocorticoid receptor antagonist mifepristone, indicating that they act on-target (Supplementary Fig. 2). Mifepristone injection into mice reduced ACBP/DBI levels at baseline but failed to prevent the 24 h fasting-induced surge in plasma ACBP/DBI (Extended Data Fig. 1q,r). The downregulation of *ACBP*/*DBI* mRNA by thyroid hormone was inhibited by knockdown of thyroid hormone receptor α and β (Extended Data Fig. 2a–d). Short-term (16 h) administration of T3 had no effect on plasma ACBP/DBI protein, but reduced *Acbp*/*Dbi* mRNA and protein in the liver (Extended Data Fig. 2e–i).

In sum, our data indicate that corticosteroids reduce the cellular content of ACBP/DBI through its autophagy-associated release into the extracellular space, while T3 downregulates ACBP/DBI expression at the mRNA level.

### Glucocorticoids and hypercortisolaemia elevate plasma ACBP

HCS significantly enhanced *ACBP*/*DBI* expression in peripheral blood mononuclear cells (PBMCs) from healthy human donors (Fig. 1n). We next analysed the expression of ACBP in two human cohorts. Results from the first cohort indicated that plasma ACBP/DBI concentrations in both female and male patients receiving glucocorticoids (together *n* = 53) were significantly higher than those in the control group (*n* = 39) (Fig. 1o,p). The second cohort comprised patients with ACTH-dependent Cushing’s syndrome. Plasma ACBP/DBI concentrations were significantly higher in patients with active disease (*n* = 11) compared with patients in remission (*n* = 13) (Fig. 1q). In patients with active disease, ACBP/DBI concentration exhibited a significant and robust positive correlation with body mass index (BMI), which is a proxy of Cushing’s syndrome severity (Fig. 1r). Moreover, in patients in remission, supplemental glucocorticoid doses correlated with ACBP/DBI (Fig. 1s).

### ACBP vaccination prevents consequences of Cushing’s syndrome

**Fig. 2:**
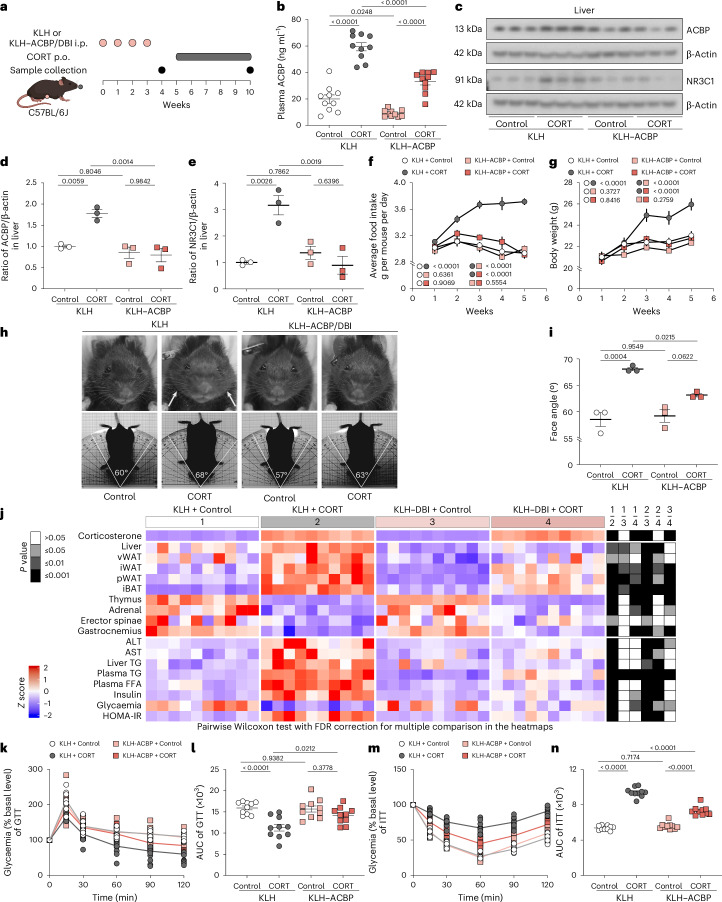
Autoantibody-mediated neutralization of ACBP/DBI prevents Cushing’s syndrome. **a**, The experimental schedule of CORT administration in auto-immunized C57BL/6J female mice against ACBP/DBI. Female C57BL/6J mice were treated with KLH–ACBP for autoimmunization or KLH alone, both administered intraperitoneally for 4 weeks (*n* = 10 mice per group). One week later, mice received CORT (100 μg ml^−1^) or vehicle control (control) in drinking water orally (p.o.) for an additional 5 weeks (*n* = 10 mice per group). **b**, Plasma levels of ACBP were measured by ELISA. **c**–**e**, Hepatic ACBP and NR3C1 were analysed by immunoblot. Representative blots (**c**) and quantifications of the indicated protein ratios (**d** and **e**) are shown (*n* = 3 per group). β-Actin was used as a loading control. **f**,**g**, The average food intake (*n* = 4 cages per group) (**f**) and body weight (*n* = 10 mice per group) (**g**) was monitored in the indicated groups. The *P* value represents the comparison of areas under the curve. **h**,**i**, Representative frontal and longitudinal photographs of one mouse of each group are shown (**h**), and facial angles were measured (**i**) at the end of week 5 (*n* = 3 mice per group). The lines indicate the measurement of the facial angle in mice. **j**, The heatmap shows the standardized deviations (*z* scores) of tissue weights relative to body weight and the quantification of various biochemical parameters across the treatment groups (*n* = 10 per group). vWAT, visceral white adipose tissue; iWAT, inguinal white adipose tissue; pWAT, perigonadal white adipose tissue; iBAT, interscapular brown adipose tissue. Statistical comparisons were performed by pairwise (two-tailed) Wilcoxon test with false discovery rate correction for multiple comparisons (*P* values are indicated). **k**–**n**, GTT (*n* = 10 mice per group) (**k**) and ITT (*n* = 10 mice per group) (**m**) were monitored in the indicated groups. The *P* value represents the comparison of areas under the curve (GTT (**l**) and ITT (**n**)). All dot plots depict mean ± s.e.m. The curves in **f** and **g** were longitudinally analysed with type II ANOVA and pairwise comparisons. The data in **b**, **d**, **e**, **i**, **l** and **n** were analysed using one-way ANOVA with Tukey correction. Source data

We speculated that (part of) the Cushing’s syndrome phenotype including increased food intake, weight gain, adiposity and type 2 diabetes (T2D) might be related to the increase in ACBP/DBI. To explore this possibility, we repeatedly immunized female C57BL/6J mice with ACBP/DBI protein coupled to the potent immunogen keyhole limpet haemocyanine (KLH) using a protocol that breaks self-tolerance against ACBP/DBI and, hence, induces neutralizing autoantibodies^34^. Immunization with KLH alone was performed as a control. Then, the mice received high-dose corticosterone (CORT, which is the primary adrenal corticosteroid in laboratory rodents) for 5 weeks in the drinking water (or 0.66% ethanol (EtOH) in water as a control) (Fig. 2a). Immunization with KLH–ACBP reduced the plasma ACBP/DBI concentration in both control and CORT-treated mice (Fig. 2b) but did not affect CORT levels (Supplementary Fig. 3a). In KLH-only immunized mice, CORT induced an increase in ACBP/DBI expression in the liver (Fig. 2c,d) and enhanced the expression of the glucocorticoid receptor NR3C1 (Fig. 2c,e). All these CORT effects were attenuated upon vaccination with KLH–ACBP (Fig. 2c–e).

**Extended Data Fig. 3:**
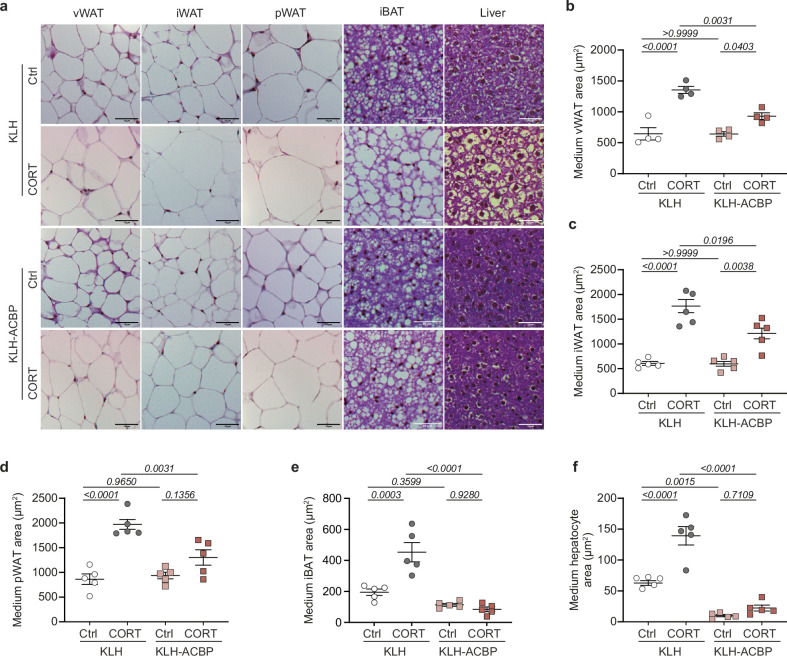
Attenuation of corticosterone-induced changes in hepatic morphology and adipose tissue by autoantibody-mediated neutralization of ACBP/DBI model. (**a**) Representative hematoxylin and eosin stains of liver, white adipose tissue (WAT), and interscapular brown adipose tissue (iBAT) of female C57BL/6J mice treated with corticosterone (CORT; 100 μg/mL or vehicle control (Ctrl) in drinking water, *p.o*.) for 5 weeks together with KLH-ACBP for autoimmunization or KLH alone both administered *i.p*. Scale bar equals 40 µm. (**b-f**) Medium area of visceral WAT (vWAT), inguinal WAT (iWAT), perigonadal WAT (pWAT), iBAT and hepatocytes was assessed in the indicated groups (*n=4-5 mice tissue sections*/*condition*). One-way ANOVA with Tukey correction was used for statistical analysis (*P-*values are indicated). All dot plots depict means ± SEM. Source data

Concomitantly, KLH–ACBP vaccination prevented the CORT-induced surge in food intake (Fig. 2f) and body weight gain (Fig. 2g). CORT administration to KLH-only immunized mice induced a major increase in body mass with an increase in the face angle reminiscent of the moon face found in patients with Cushing’s syndrome (Fig. 2h,i). At necropsy, signs of CORT-induced thymolysis, atrophy of the adrenal gland and sarcopenia affecting the erector spinae were not prevented by KLH–ACBP vaccination (Fig. 2j and Supplementary Fig. 3). However, the increase of liver weight, visceral, inguinal, perigonadal WAT and interscapular brown adipose tissue (iBAT), which was accompanied by an increase in median adipocyte diameter (Extended Data Fig. 3), was attenuated by ACBP/DBI autoantibodies. In addition, the CORT-induced increase in liver triglycerides (TG) and circulating liver enzymes including alanine aminotransferase (ALT) and aspartate aminotransferase (AST) was prevented by KLH–ACBP vaccination. Concomitantly, an elevation of circulating TG and free fatty acids (FFA), alterations in the glucose tolerance test (GTT) and insulin tolerance tests (ITT) and signs of T2D such as hyperinsulinaemia and altered fasting plasma glucose, yielding increased homeostasis model assessment of insulin resistance (HOMA-IR) values, were found in KLH-only vaccinated mice treated with CORT, but not after KLH–ACBP vaccination (Fig. 2j and Supplementary Fig. 3).

In sum, autoantibodies neutralizing ACBP/DBI blunt major phenotypic and metabolic manifestations of Cushing’s syndrome including an increase in appetite, weight gain, hypertrophy of WAT and iBAT, liver damage, dyslipidaemia and insulin resistance.

### Genetic inhibition of ACBP prevents Cushing’s syndrome

Autoantibodies against ACBP/DBI might mediate off-target and side effects due to immune complex disease. To rule out this possibility, we attempted to inhibit Cushing’s syndrome by two alternative methods, namely, (1) tamoxifen-inducible expression of a CRE recombinase that excises the floxed intron 2 of the gene encoding ACBP/DBI, thus leading to its conditional ablation, either at the whole-body level or in hepatocytes alone, and (2) a point mutation (F77I in subunit GABRG2) in the ACBP/DBI receptor, GABA_A_R, that abolishes its interaction with ACBP/DBI^10,11^.

**Fig. 3:**
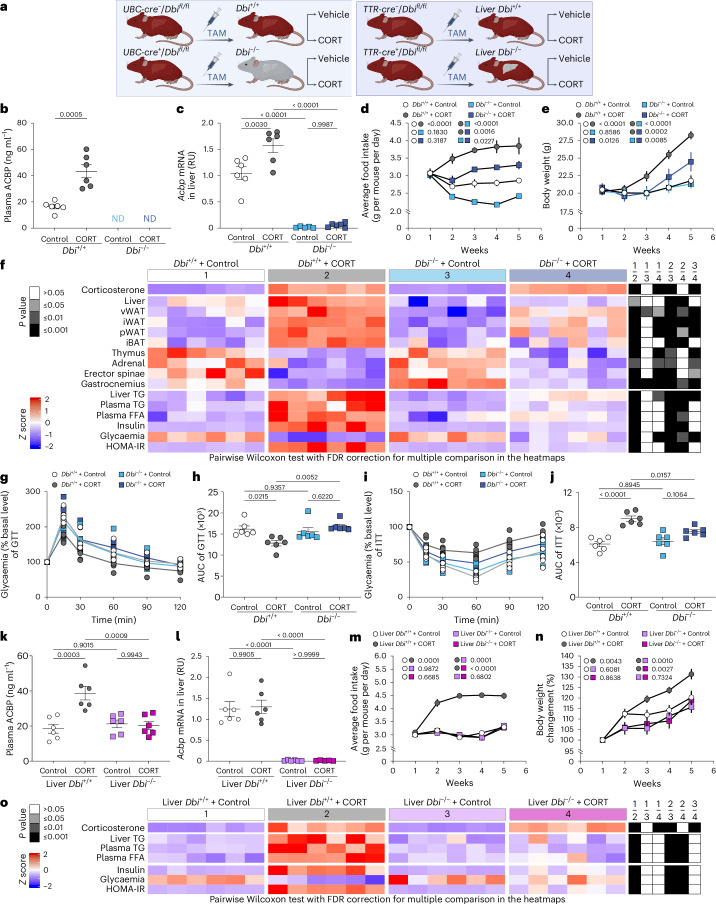
Genetic depletion of ACBP/DBI prevents Cushing’s syndrome. **a**, A schematic representation of the different C57BL/6J lineages conditionally knocked out for the ACBP/DBI protein. The conditional knockout of *Acbp*/*Dbi* was achieved by administering repeated i.p. injections of tamoxifen (TAM) to mice with a floxed *Acbp*/*Dbi* gene (genotype: *Dbi*^*fl/fl*^), combined with either a latent ubiquitous CRE recombinase (UBC-cre^+^) or a hepatocyte-specific CRE recombinase (TTR-cre^+^). **b**–**o**, Female *Dbi*^−*/*−^ mice and their wild-type controls (*Dbi*^*+/+*^) as well as female liver-*Dbi*^−*/*−^ mice and their wild-type controls (liver-*Dbi*^*+/+*^) were treated with CORT (100 μg ml^−1^) or vehicle (control) in drinking water (p.o.) for 5 weeks: Plasma ACBP was quantified by ELISA (*n* = 6 per group) (**b** and **k**), and hepatic *Acbp* mRNA was assessed by qRT–PCR (*n* = 6 per group) (**c** and **l**); average food intake (*n* = 3 cages per group) (**d** and **m**) and body weight (*n* = 8 and 6 mice per group) (**e** and **n**) were monitored in the indicated groups (the *P* value represents the comparison of areas under the curve); the heatmap shows the standardized deviations (*z* scores) of tissue weights relative to body weight and the quantification of various biochemical parameters across the treatment groups (*n* = 6 mice per group) (**f** and **o**) (statistical comparisons were performed by pairwise (two-tailed) Wilcoxon test with false discovery rate (FDR) correction for multiple comparisons; *P* values are indicated); GTT (*n* = 6 mice per group) (**g**) and ITT (*n* = 6 mice per group) (**i**) were monitored in the indicated groups (the *P* value represents the comparison of areas under the curve; **h** and **j**). Statistical comparisons were performed by pairwise Wilcoxon test with FDR correction for multiple comparisons in the heatmaps (*P* values are indicated). All dot plots depict mean ± s.e.m. Two independently repeated experiments were conducted; only one representative result is shown. The curves in **d**, **e**, **m** and **n** were longitudinally analysed with type II ANOVA and pairwise comparisons. The data in **b**, **c**, **h** and **j**–**l** were analysed using one-way ANOVA with Tukey correction. vWAT, visceral white adipose tissue; iWAT, inguinal white adipose tissue; pWAT, perigonadal white adipose tissue; iBAT, interscapular brown adipose tissue; ND, not detectable. Created with BioRender.com. Source data

The conditional *Acbp*/*Dbi* knockout^14^ was achieved by repeated intraperitoneal (i.p.) injection of tamoxifen into female mice bearing a floxed *Acbp*/*Dbi* gene (genotype: *Dbi*^*fl/fl*^) in combination with a latent ubiquitous or a hepatocyte-specific CRE recombinase (genotypes: *UBC-cre-ERT2* or *TTR-creTam*, respectively). *Dbi*^*fl/fl*^ mice lacking CRE were also injected with tamoxifen as controls (Fig. 3a). Then, the animals were treated with CORT in drinking water for 5 weeks. The whole-body *Acbp*/*Dbi* knockout^14^ rendered circulating ACBP/DBI and hepatic *Acbp*/*Dbi* mRNA undetectable (Fig. 3b,c), reduced CORT-induced appetite (Fig. 3d) and weight gain (Fig. 3e) and had no effects on CORT plasma levels (Supplementary Fig. 4a), but attenuated the CORT-induced hepatomegaly, expansion of adipose tissues, atrophy of the skeleton muscle, dyslipidaemia, insulin resistance fasting plasma glucose, HOMA-IR values and alterations in the GTT and ITT (Fig. 3f–j and Supplementary Fig. 4). Although hepatocyte-specific knockout did not reduce baseline ACBP/DBI plasma concentrations to undetectable levels (as this occurs in the whole-body knockout), it did prevent the CORT-induced surge in circulating ACBP/DBI, indicating that this elevation stems from CORT effects on the liver (Fig. 3k), in which *Acbp*/*Dbi* mRNA became undetectable (Fig. 3l). This effect of the hepatocyte-specific *Dbi* knockout correlated with reduced CORT-induced food intake (Fig. 3m) and weight gain (Fig. 3n), as well as the normalization of hepatomegaly, dyslipidaemia, insulin resistance, fasting plasma glucose and HOMA-IR values (Fig. 3o and Supplementary Fig. 5).

**Fig. 4:**
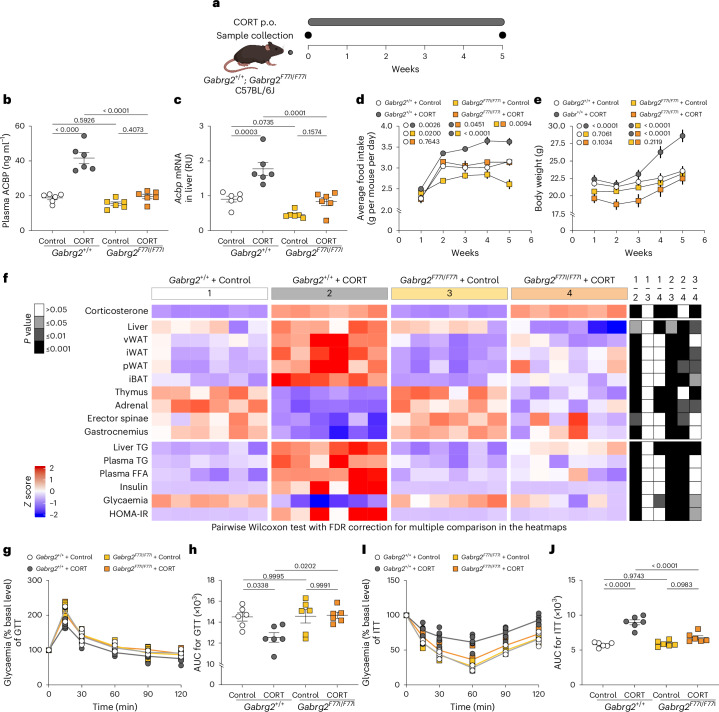
Genetic inhibition of ACBP/DBI prevents Cushing’s syndrome. **a**, A scheme showing the experimental schedule of CORT administration in female C57BL/6J mice (*Gabrg2*^*F77I/F77I*^) or wild-type controls (*Gabrg2*^*+/+*^). **b**–**j**, *Gabrg2*^*F77I/F77I*^ or *Gabrg2*^*+/+*^ mice were treated with CORT (100 μg ml^−1^ or vehicle control (control) in drinking water, p.o.) for 5 weeks (*n* = 10, 10, 9 and 10 mice per group): plasma ACBP was measured by ELISA (*n* = 6 per group) (**b**), and hepatic *Acbp* mRNA was assessed by qRT–PCR (*n* = 6 per group; AU, arbitrary units) (**c**); average food intake (*n* = 3 cages per group) (**d**) and body weight (*n* = 10, 10, 9 and 10 mice per group) (**e**) were monitored in the indicated groups (*P* values compare areas under the curve); the heatmap shows the standardized deviations (*z* scores) of tissue weights relative to body weight and the quantification of various biochemical parameters across the treatment groups (*n* = 6 mice per group) (**f**) (statistical comparisons were performed by pairwise (two-tailed) Wilcoxon test with false discovery rate (FDR) correction for multiple comparisons: *P* values are indicated); GTT (*n* = 6 mice per group) (**g**) and ITT (*n* = 6 mice per group) (**i**) were monitored in the indicated groups (the *P* value represents the comparison of areas under the curve; (GTT (**h**) and ITT (**j**)). Statistical comparisons were performed by pairwise Wilcoxon test with FDR correction for multiple comparison in the heatmaps (*P* values are indicated). All dot plots depict mean ± s.e.m. Two independent repeated experiments were conducted; only one representative result is shown. The curves in **d** and **e** were longitudinally analysed with type II ANOVA and pairwise comparisons. The data in **b**, **c**, **h** and **j** were analysed using one-way ANOVA with Tukey correction. vWAT, visceral white adipose tissue; iWAT, inguinal white adipose tissue; pWAT, perigonadal white adipose tissue; iBAT, interscapular brown adipose tissue. Created with BioRender.com. Source data

Mice homozygous for the *Gabrg2* F77I mutation (genotype: *Gabrg2*^*F77I/F77I*^) were compared with their wild type (genotype: *Gabrg2*^*+/+*^) controls (Fig. 4a). The *Gabrg2* F77I mutation blunted the CORT-induced augmentation of plasma ACBP/DBI (Fig. 4b), reduced the CORT-elicited increase in hepatic *Acbp*/*Dbi* mRNA expression (Fig. 4c) and prevented the surge in food intake (Fig. 4d) and weight gain (Fig. 4e). The *Gabrg2* F77I mutation had no effects on CORT levels but attenuated the CORT-induced atrophy of thymus, adrenal gland and skeleton muscle as it reversed the expansion of WAT and iBAT, as well as liver hypertrophy (Fig. 4f and Supplementary Fig. 6). *Gabrg2* F77I mutation also prevented CORT-induced signs of dyslipidaemia, hyperinsulinaemia and insulin resistance (Fig. 4f–j and Supplementary Fig. 6).

In conclusion, it appears that genetic inhibition of the ACBP/DBI system can prevent weight gain and other metabolic manifestations of Cushing’s syndrome induced by CORT, strongly suggesting that the results obtained with autoantibodies are due to on-target effects.

### Antibody neutralization of ACBP prevents Cushing’s syndrome

**Fig. 5:**
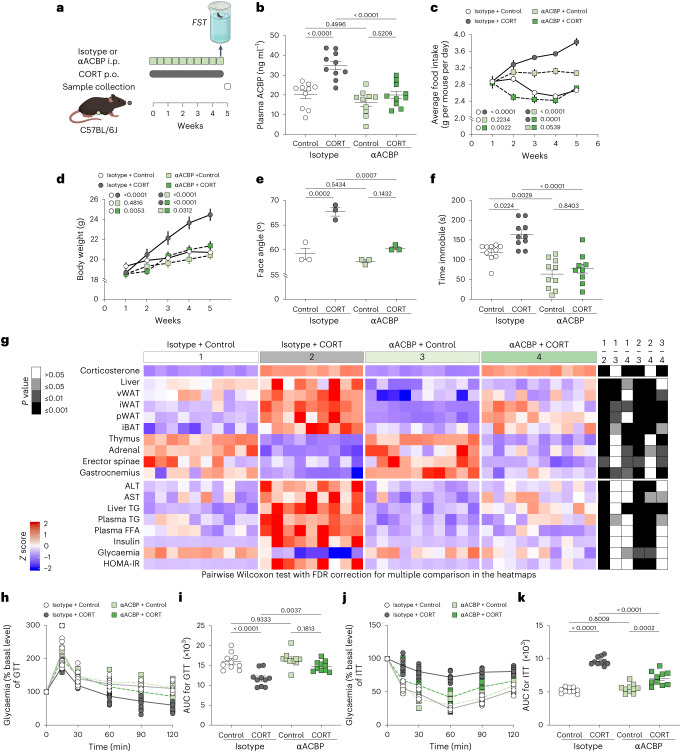
Passive immunization of mice by neutralizing monoclonal anti-ACBP/DBI mAb prevents the manifestation of Cushing’s syndrome. **a**, The experimental schedule for passive immunization. Female C57BL/6J mice were treated with CORT (100 μg ml^−1^ or vehicle control (control) in drinking water, p.o.) for 5 weeks together with ACBP/DBI mAb (αACBP, 5 mg kg^−1^ body weight, i.p., semiweekly). Isotype was used as the control. Animals were subjected to FST in the fifth week. **b**, Plasma ACBP was measured by ELISA in the indicated treatment groups (*n* = 10 per group). **c**,**d**, The average food intake (*n* = 4 cages per group) (**c**) and body weight (*n* = 10 per group) (**d**) was monitored in the indicated groups. **e**, Facial angles of mice from the indicated groups were measured (*n* = 3 mice per group). **f**, Immobility time assessed by FST (*n* = 10 mice per group). **g**, The heatmap shows the standardized deviations (*z* scores) of tissue weight relative to body weight and the quantification of various biochemical parameters across the treatment groups (*n* = 10, 9, 10 and 10 mice per group). Statistical comparisons were performed by pairwise (two-tailed) Wilcoxon test with false discovery rate (FDR) correction for multiple comparisons (*P* values are indicated). **h**–**k**, GTT (*n* = 10 mice per group) (**h**) and ITT (*n* = 10 mice per group) (**j**) were monitored in the indicated groups. The *P* value represents the comparison of areas under the curve (GTT (**i**) and ITT(**k**)). The *P* value represents the comparison of areas under the curve. Statistical comparisons were performed by pairwise Wilcoxon tests with FDR correction for multiple comparisons in the heatmaps (*P* values are indicated). All dot plots represent mean ± s.e.m. The curves in **c** and **d** were longitudinally analysed with type II ANOVA and pairwise comparisons. The data in **b**, **e**, **f**, **i** and **k** were analysed using one-way ANOVA with Tukey correction. vWAT, visceral white adipose tissue; iWAT, inguinal white adipose tissue; pWAT, perigonadal white adipose tissue; iBAT, interscapular brown adipose tissue. Created with BioRender.com. Source data

**Fig. 6:**
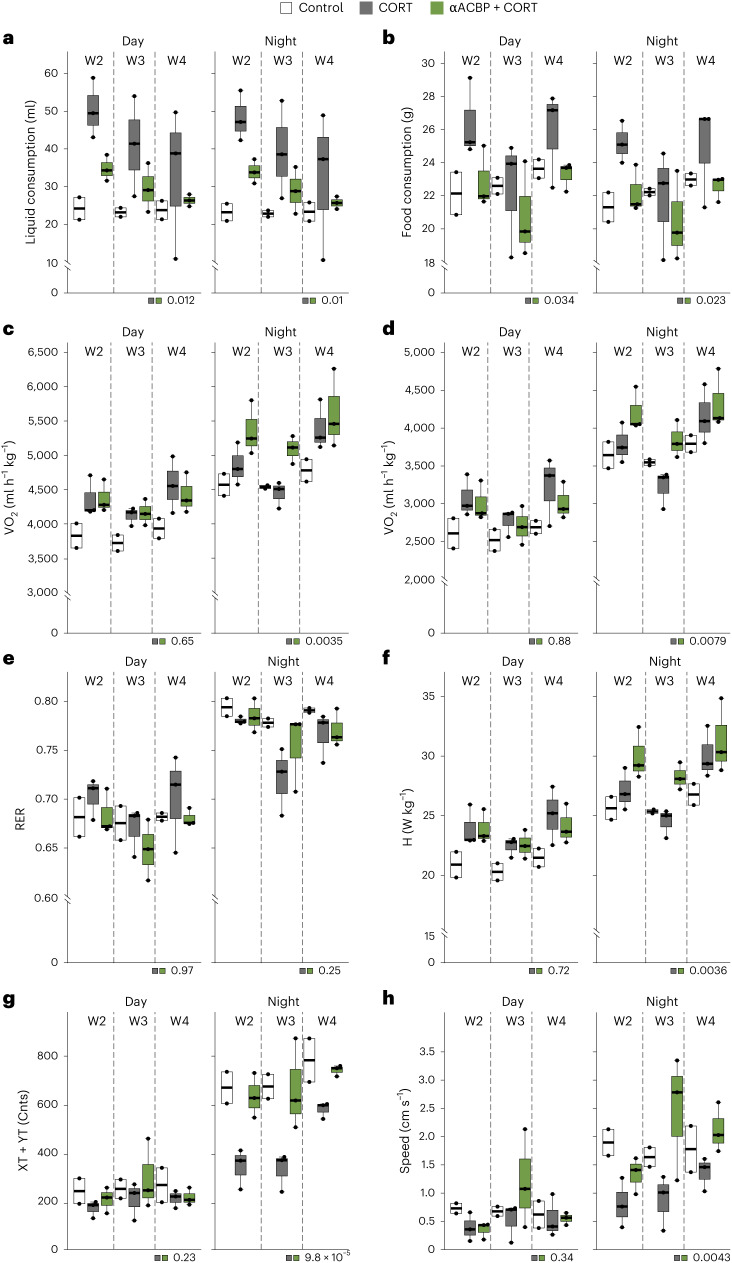
Metabolic effects of ACBP/DBI neutralization in mice undergoing glucocorticoid treatment. Metabolic parameters in mice subjected to vehicle, CORT or a combination of CORT and ACBP/DBI mAb (αACBP, 5 mg kg^−1^ body weight, i.p., semiweekly) for 4 weeks. **a**–**h**, Liquid consumption (**a**), food consumption (**b**), O_2_ consumption (VO_2_, **c**), CO_2_ production (VCO_2_, **d**), respiratory exchange rates (RER, **e**), heat production (H, **f**), sum of the ambulatory and fine movements (XT + YT yielding counts (Ctns), **g**), and speed (**h**) were measured in metabolic cages. Daily CORT exposure was adjusted to 500 µg. The data are presented as standard box plots (the centre line represents the median, box limits represent upper and lower quartiles, and whiskers represent minimum and maximum values) of metabolic parameters (*n* = 2, 3 and 3 mice per condition). Metabolic parameters are means evaluated over each 12 h period (night or day) and further averaged over every night and day period for each animal and for each week. For liquid and food consumption (**a** and **b**), cumulative values over 12 h periods are used instead of means. The *P* values were calculated by Fisher’s meta-analysis method. Source data

**Extended Data Fig. 4:**
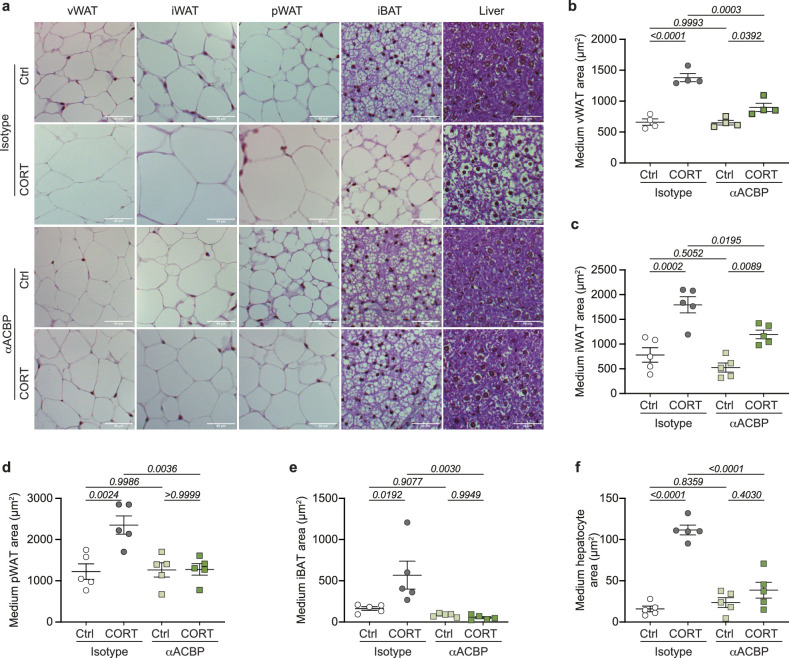
Attenuation of corticosterone-induced changes in hepatic morphology and adipose tissue by ACBP/DBI mAb-mediated neutralization of ACBP/DBI. (**a**) Representative hematoxylin and eosin stains of liver, white adipose tissue (WAT), and interscapular brown adipose tissue (iBAT) of female C57BL/6J mice treated with corticosterone (CORT) (100 μg/mL, or vehicle (Ctrl) in drinking water, *p.o*.) with or without αACBP (5 mg/kg body weight, *i.p*.) semiweekly for 5 weeks. Scale bar equals 40 µm. (**b-f**) Medium area of visceral WAT (vWAT), inguinal WAT (iWAT), perigonadal WAT (pWAT), iBAT and hepatocytes was assessed in the indicated groups (*n=4-5 mice tissue sections*/*condition*). One-way ANOVA with Tukey correction was used for statistical analysis (*P-*values are indicated). All dot plots depict means ± SEM. Source data

We next determined whether passive immunization of mice using a monoclonal antibody (mAb) specific for ACBP/DBI (αACBP, injected twice weekly at a dose of 5 mg kg^−1^ body weight) would be capable of preventing the Cushing’s phenotype (Fig. 5a). This protocol succeeded in neutralizing the increase of circulating ACBP/DBI induced by CORT (Fig. 5b) and prevented the increase in food intake (Fig. 5c), weight gain (Fig. 5d), adipocyte hypertrophy (Extended Data Fig. 4) and augmented face angle (Fig. 5e). A forced swimming experiment revealed that, compared with the control group, mice receiving CORT exhibited a longer immobility (a proxy of depression-like behaviour), while ACBP/DBI neutralization shortened immobility (Fig. 5f). Hence, ACBP/DBI inhibition can prevent CORT-induced behavioural changes. αACBP also largely prevented CORT-induced increases in circulating ALT and AST, liver and plasma TG, plasma FFA and hyperinsulinaemia, alterations in glycaemia suggestive of insulin resistance^35,36^, and elevated HOMA-IR values (Fig. 5g and Supplementary Fig. 7). Accordingly, αACBP normalized the CORT-induced alteration in glucose tolerance and insulin tolerance (Fig. 5h–k). Inhibition of ACBP/DBI by αACBP reversed the hypertrophy of adrenal, liver and adipose tissues but failed to prevent CORT-induced muscular and thymic atrophy (Fig. 5g and Supplementary Fig. 7). Experiments in metabolic cages revealed that αACBP reduced CORT-induced hyperdipsia (Fig. 6a) and hyperphagia (Fig. 6b) and had little effect on the respiratory exchange ratio (Fig. 6c–e) but enhanced nocturnal energy expenditure (Fig. 6f), ambulatory and fine movements (Fig. 6g) and speed of movement (Fig. 6h).

**Extended Data Fig. 5:**
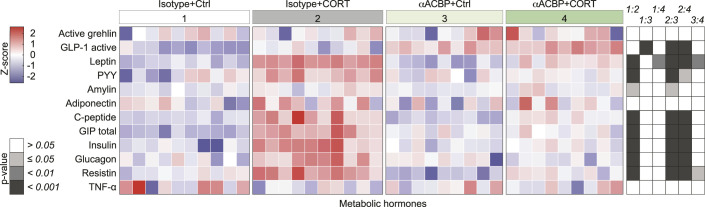
Effect of corticosterone and anti-ACBP/DBI mAb on plasma hormone concentrations. Female C57BL/6J mice were treated with corticosterone (CORT) (100 μg/mL, or vehicle (Ctrl) in drinking water, *p.o*.) with or without αACBP (5 mg/kg body weight, *i.p*.) semiweekly for 5 weeks. Abundance of plasma hormones was assessed by Luminex multiplex assay (*n=10,10,9,10*/*group*). The heatmap displays z-scores of the indicated metabolic hormones in the indicated treatment groups. Statistical comparisons were performed by pairwise (two tailed) Wilcoxon test with FDR correction for multiple comparisons. (*P*-values are indicated). Source data

**Extended Data Fig. 6:**
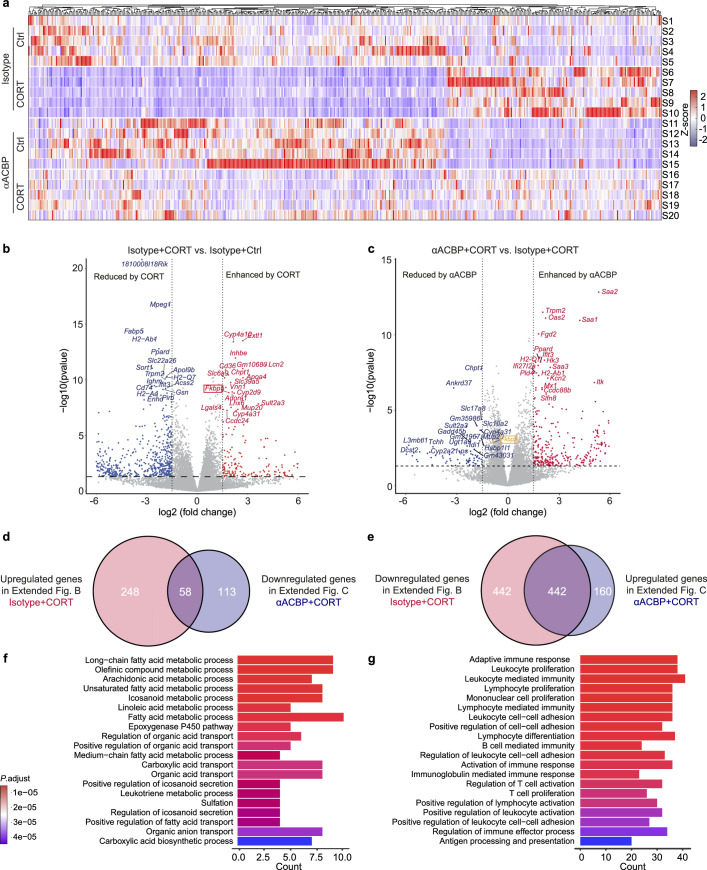
Liver RNA sequencing analysis of anti-ACBP/DBI mAb model. (**a**) At the end of the corticosterone (CORT) and anti-ACBP/DBI (αACBP) mAb experiment, liver tissue was collected for RNAseq analysis (*n=5/group*). Genes with a differential expression *P*-value ≤ 0.05 (Wald test) and abs (fold change) ≥ 2 were selected in (a). (**b**) Volcano plot of differential genes between isotype + CORT and isotype + Ctrl groups. (**c**) Volcano plot of differential genes between αACBP + CORT and isotype + CORT groups. Log2 (fold change)| ≥ 1, *P*-value < 0.05. (**d,e**) Venn diagrams illustrate overlaps of the transcriptomic CORT effects on isotype and αACBP mAb neutralization. (**f**) GO enrichment analysis of the genes obtained from the overlap in (**d**) (58 genes). (**g**) GO enrichment analysis of the genes obtained from the overlap in (**e**) (442 genes). Data were analyzed using R software with the DESeq2 package. Source data

**Extended Data Fig. 7:**
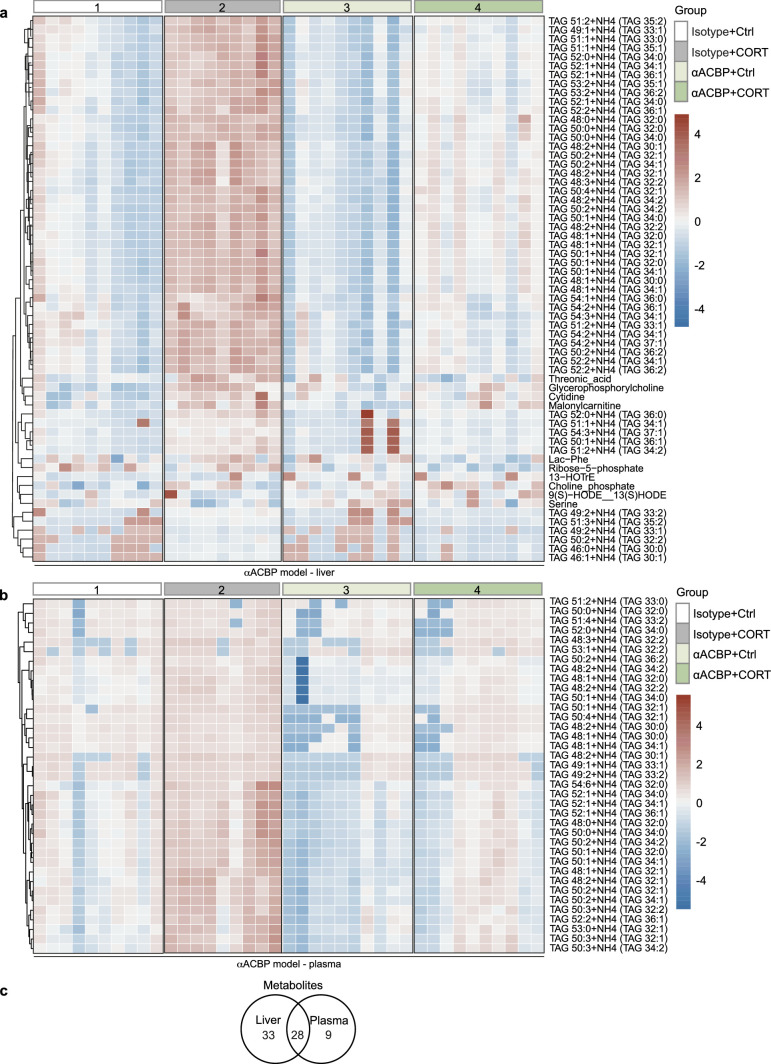
Liver and plasma metabolomics of Cushing’s syndrome treated with anti-ACBP/DBI mAb. (**a**) Heatmap showing metabolite profiling of liver from anti-ACBP/DBI (αACBP) mAb model. Student’s t test was used to compare isotype + corticosterone (CORT) versus αACBP + CORT, and metabolites which have less than 0.05 were kept for the heatmap. Data was Area Quality Control Corrected Log2 transformed and centered on the mean of all the biological samples. (**b**) Heatmap showing metabolite profiling of plasma from αACBP mAb treated mice. Student’s test was used to compare isotype + CORT Versus αACBP + CORT, and metabolites which have less than 0.05 were kept for the Heatmap. Data was Area Quality Control Corrected Log2 transformed and centered on the mean of all the biological samples. (**c**) Venn Diagram displaying the repartition across liver and plasma of the metabolites shown in **E7a** and **E7b**. Twenty-eight metabolites are common over the two biological matrices and are detailed in Supplementary Figs. 8,9. Data were analyzed using R software. Source data

Multi-omics analyses supported the idea that ACBP/DBI neutralization normalized most metabolic alterations induced by CORT. Thus, αACBP attenuated the CORT-induced hyperleptinaemia, as well as the increase in peptide tyrosine tyrosine, C-peptide, glucose-dependent insulinotropic polypeptide, glucagon and resistin (Extended Data Fig. 5). We performed RNA-sequencing-based transcriptomic analyses of liver tissues, choosing this organ because it is the principal source of CORT-induced ACBP/DBI. Of note, αACBP reversed most of the transcriptional changes induced by CORT (Extended Data Fig. 6a–e). At the transcriptional level, αACBP reduced CORT-elicited lipo-anabolic pathways (Extended Data Fig. 6f) but upregulated CORT-repressed immune-related genes in the liver (Extended Data Fig. 6g). Finally, mass spectrometric metabolomics of the liver and plasma confirmed a surge in TG metabolites induced by CORT that was prevented by ACBP/DBI neutralization (Extended Data Fig. 7 and Supplementary Figs. 8 and 9).

**Fig. 7:**
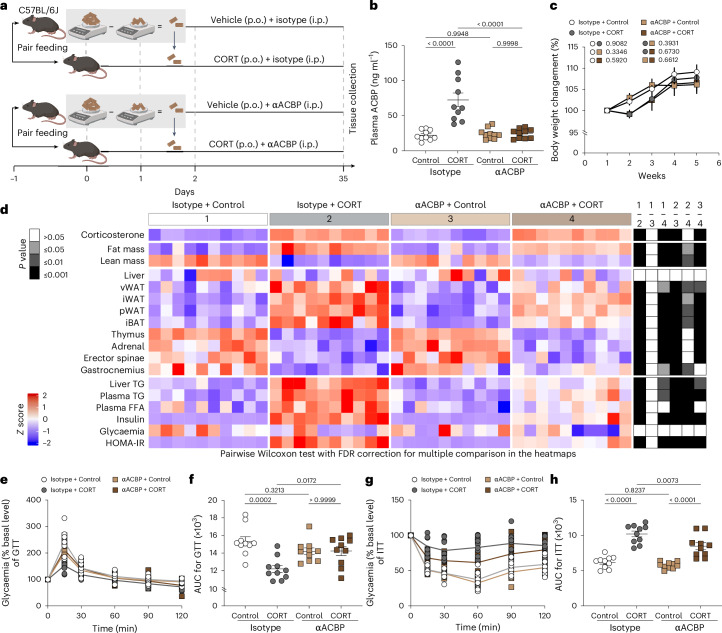
Mitigation of CORT-induced metabolic dysregulation in mice under pair feeding by ACBP/DBI neutralization. **a**, The experimental schedule for the pair-feeding experiment in C57BL/6J female mice receiving anti-ACBP/DBI antibody, isotype control antibody and/or CORT. A pair-feeding protocol was implemented where the average daily food intake of vehicle groups was measured daily to limit the amount of food provided to the other groups. **b**, Plasma ACBP was measured by ELISA (*n* = 10 per group). **c**, Body weight modifications (*n* = 10 per group) were monitored. **d**, The heatmap shows the standardized deviations (*z* scores) of tissue weights relative to body weight and the quantification of various biochemical parameters across the treatment groups (*n* = 10 mice per group). Statistical comparisons were performed by pairwise (two-tailed) Wilcoxon test with false discovery rate (FDR) correction for multiple comparisons (*P* values are indicated). **e**–**h**, GTT (*n* = 10 per group) (**e** and **f**) and ITT (*n* = 10 per group) (**g** and **h**) are shown (**e** and **g**) with the comparison of the areas under the curve (**f** and **h**). Statistical comparisons were performed by pairwise Wilcoxon tests with FDR correction for multiple comparison in the heatmaps. All dot plots depict mean ± s.e.m. The weight curves were longitudinally analysed with type II ANOVA and pairwise comparisons. The data in **b**, **f** and **h** were analysed using one-way ANOVA with Tukey correction. vWAT, visceral white adipose tissue; iWAT, inguinal white adipose tissue; pWAT, perigonadal white adipose tissue; iBAT, interscapular brown adipose tissue. Created with BioRender.com. Source data

**Extended Data Fig. 8:**
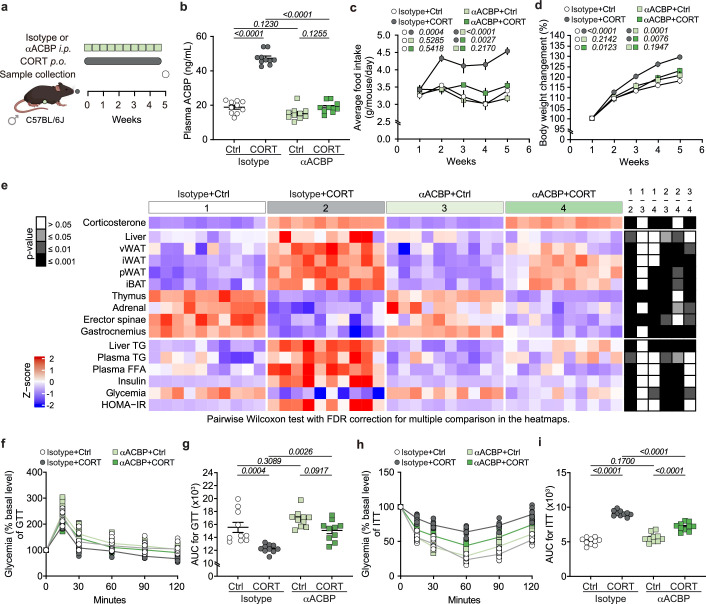
A monoclonal anti-ACBP/DBI antibody prevents the manifestation of Cushing’s syndrome in male mice. (**a**) Male C57BL/6J mice were treated with CORT (100 μg/mL) in drinking water (*p.o*.) for 5 weeks (*n=10/group*). (**b**) Plasma ACBP was quantified by ELISA (*n=10/group*). (**c**) Average food intake (*n=3 cages/group*) and (**d**) body weight (*n=10 mice/group*) were monitored. *P*-values compare areas under the curve. (**e**) The heatmap shows the standardized deviations (z-scores) of tissue weights relative to body weight and the quantification of various biochemical parameters across the treatment groups (*n=10 mice*/*group*). Statistical comparisons were performed by pairwise (two tailed) Wilcoxon test with FDR correction for multiple comparisons. (*P*-values are indicated). (**f-i**) Glucose tolerance tests (GTT) (*n=10 mice*/*group*) (**f**) and insulin tolerance tests (ITT) (*n=10 mice*/*group*) (**h**) were performed in the indicated groups. *P*-value refer to the comparison of areas under the curve (**g,i**). Statistical comparisons were performed by pairwise Wilcoxon tests with FDR correction for multiple comparison in the heatmaps. All dot plots depict means ± SEM. All curves were longitudinally analyzed with type II ANOVA and pairwise comparisons (**c,d**). One-way ANOVA with Tukey correction was used for statistical analysis (*P-*values are indicated) (**d,g,i**). Source data

All the aforementioned results have been obtained in female mice. To exclude any possible sexual dimorphism, we performed experiments in male C57BL/6J mice to demonstrate that CORT-induced hyperphagy, weight gain and metabolic syndrome are largely abolished by αACBP (Extended Data Fig. 8 and Supplementary Fig. 10). We also performed pair-feeding experiments in female mice to investigate whether αACBP solely interferes with CORT-induced metabolic syndrome by suppressing hyperphagy (Fig. 7a). The CORT induced increase of circulating ACBP/DBI was attenuated by αACBP (Fig. 7b). Even when pair feeding was performed in a way that the body weight of the animals treated with vehicle only, CORT, alone or in combination with the αACBP antibody was undistinguishable (Fig. 7c), the metabolic effects of CORT persisted (Fig. 7d and Supplementary Fig. 11). Thus, under these conditions, CORT caused dyslipidaemia (enhanced TG and FFA), hyperinsulinaemia, a shift in body composition from lean mass to fat mass determined by nuclear magnetic resonance relaxometry, an increase in adiposity and a reduction of muscle mass. Most of these signs of CORT-induced metabolic syndrome were attenuated by ACBP/DBI neutralization **(**Fig. 7d–h and Supplementary Fig. 11).

It is noteworthy that αACBP mAb completely failed to prevent weight gain and food intake (Supplementary Fig. 12a–c) induced by the antidepressant citalopram, which, in contrast to CORT, failed to increase ACBP/DBI in the plasma (Supplementary Fig. 12d,e) and ACBP/DBI depletion in the liver (and to upregulate ACBP/DBI in WAT) (Supplementary Fig. 12f–i). This finding suggests that ACBP/DBI neutralization is reducing appetite only when ACBP/DBI is elevated in the circulation.

In conclusion, a mAb neutralizing ACBP/DBI phenocopies the effects of autoantibody-mediated or genetic inhibition of ACBP/DBI, thus preventing the metabolic manifestations of Cushing’s syndrome.

### Endocrine inhibition of ACBP avoids Cushing’s syndrome

**Extended Data Fig. 9:**
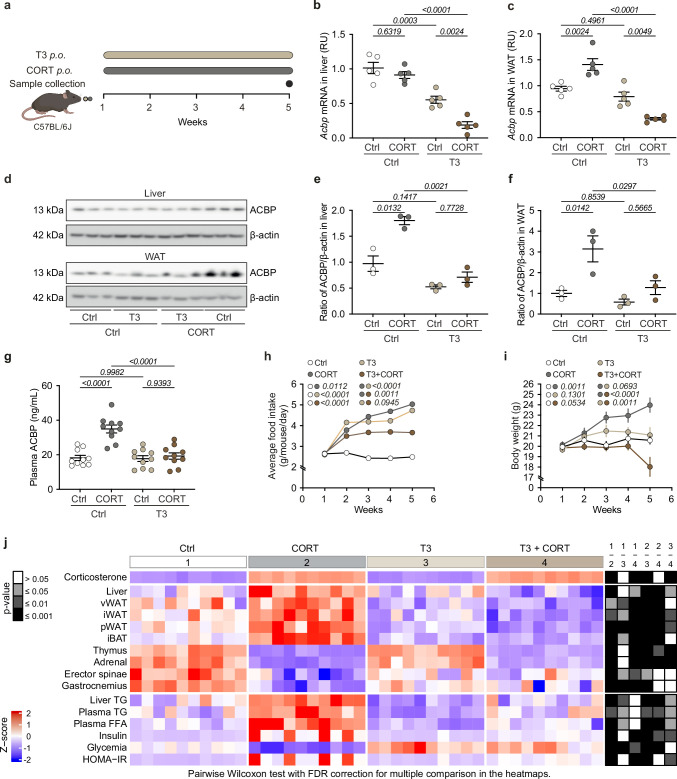
Effects of triiodothyronine (T3) administration on ACBP expression and metabolic parameters in mice under corticosterone treatment. (**a**) Scheme showing the experimental schedule of triiodothyronine (T3; *i.p*.) administration to female C57BL/6J mice treated with corticosterone (CORT) for 5 weeks. Liver (**b**) and WAT (**c**) *Acbp* mRNA levels (*n=6/group;* RU, relative units) were quantified. (**d**) Representative immunoblot shows ACBP level treated with different doses of T3 (*n=3/group*). β-actin was used as a loading control. Scatter plots show the ratio of ACBP/β-actin in liver (**e**) and in WAT (**f**). (**g**) Plasma ACBP were assessed by ELISA (*n=10*/*group*). (**h**) Average food intake (*n=3 cages/group*) and (**i**) body weight (*n=10 mice/group*) were monitored. (**j**) The heatmap shows the standardized deviations (z-scores) of tissue weights relative to body weight and the quantification of various biochemical parameters (*n=10 mice*/*group*). Statistical comparisons were performed by pairwise (two tailed) Wilcoxon test with FDR correction for multiple comparisons. (*P*-values are indicated). Statistical comparisons were performed by pairwise Wilcoxon tests with FDR correction for multiple comparison in the heatmaps (*P*-values are indicated). All dot plots indicate means ± SEM. All curves were longitudinally analyzed with type II ANOVA and pairwise comparisons (**h,i**). One-way ANOVA with Tukey correction was used for statistical analysis (*P-*values are indicated) (**b,c,e-g**). Source data

As indicated above, T3 downregulates *Acbp*/*Dbi* mRNA expression. We co-administered CORT and T3 over 5 weeks (Extended Data Fig. 9a), finding that this treatment led to a reduction in *Acbp*/*Dbi* mRNA in the liver and WAT, especially if combined with CORT (Extended Data Fig. 9b,c). Similarly, the levels of ACBP/DBI protein detectable in liver and WAT were lower in mice treated with CORT plus T3 than in animals receiving CORT alone (Extended Data Fig. 9d–f). Co-administration of T3 also reduced CORT-induced ACBP/DBI in plasma to normal levels (Extended Data Fig. 9g). As reported^37^, T3 alone stimulated appetite, and T3 was unable to prevent appetite stimulation by CORT (Extended Data Fig. 9h). However, T3 prevented weight gain induced by CORT (Extended Data Fig. 9i). The co-administration did not attenuate muscular atrophy. However, the increase in fat mass and liver weight as well as biochemical signs of metabolic syndrome due to CORT were suppressed when co-administered with T3 (Supplementary Fig. 13).

**Fig. 8:**
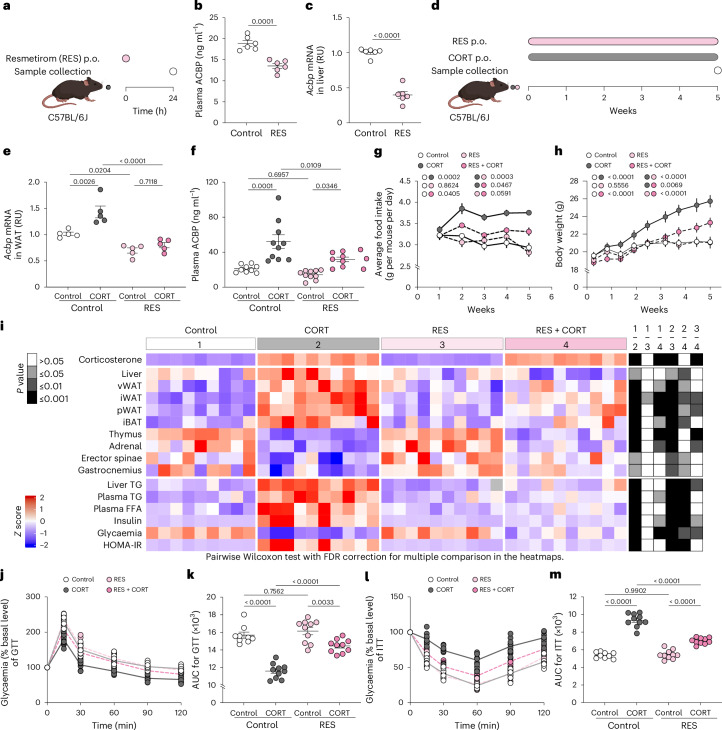
The THR-β agonist RES inhibits ACBP/DBI and prevents Cushing’s syndrome. **a**, The experimental schedule showing the treatment of female C57BL/6J mice with RES (0.033 mg ml^−1^ or vehicle control (control) per gavage). **b**,**c**, Plasma ACBP (**b**) and liver *Acbp* mRNA (**c**) were measured by ELISA and qRT–PCR, respectively (*n* = 6 per group*;* RU, relative units). **d**, The experimental schedule showing the treatment of mice with CORT (100 μg ml^−1^, p.o.) and RES (0.033 mg ml^−1^ in drinking water, p.o.) for 5 weeks. **e**,**f**, WAT *Acbp* mRNA (*n* = 5 per group) (**e**) and plasma ACBP (*n* = 9–10 per group) (**f**) were measured at the end of the fifth week in the indicated groups. **g**,**h**, The average food intake (*n* = 3 cages per group) (**g**) and body weight (*n* = 9–10 mice per group) (**h**) were monitored. *P* values refer to the comparison of areas under the curve. **i**, The heatmap shows the standardized deviations (*z* scores) of tissue weight relative to body weight and the quantification of various biochemical parameters across the treatment groups (*n* = 9, 10, 10 and 10 mice per group). Statistical comparisons were performed by pairwise (two-tailed) Wilcoxon test with false discovery rate (FDR) correction for multiple comparisons (*P* values are indicated). **j**–**m**, GTT (*n* = 9–10 mice per group) (**j**) and ITT (*n* = 9–10 mice per group) (**l**) was monitored in the indicated groups. *P* values refer to the comparison of areas under the curve (GTT (**k**) and ITT (**m**)). Statistical comparisons were performed by pairwise Wilcoxon tests with FDR correction for multiple comparison in the heatmaps. All dot plots depict means ± s.e.m. Arbitrary units, AU. The data in **b** and **c** were analysed by unpaired *t*-test (two tailed). The curves in **g** and **h** were longitudinally analysed with type II ANOVA and pairwise comparisons. The data in **e**, **f**, **k** and **m** were analysed using one-way ANOVA with Tukey correction. Created with BioRender.com. Source data

The therapeutic window of T3 is notoriously small^38^. We therefore resorted to the selective thyroid hormone receptor β (THR-β) agonist resmetirom (RES), which has been approved by the Food and Drug Administration (FDA) for the treatment of non-alcoholic steatohepatitis^39^. RES reduced ACBP/DBI expression in HepG2 cells as efficiently as T3 or T4, and this effect was blunted by knockdown of THR-β (Supplementary Fig. 14). RES led to a reduction of ACBP/DBI in plasma (Fig. 8a,b) and decreased *Acbp*/*Dbi* mRNA levels in the liver (Fig. 8c). Co-administration of CORT with RES (Fig. 8d) similarly reduced *Acbp*/*Dbi* mRNA in WAT (Fig. 8e) and ACBP/DBI protein in plasma (Fig. 8f). In contrast to T3, RES was able to prevent appetite stimulation by CORT (Fig. 8g). Accordingly, RES prevented weight gain induced by CORT (Fig. 8h). RES did not attenuate adrenal and thymic atrophy but fully reversed the increase in body fat and liver weight induced by CORT, abolished all biochemical signs of dyslipidaemia and T2D, and mediated partial effects on sarcopenia (Fig. 8i–m and Supplementary Fig. 15).

In conclusion, T3 as well as the selective THR-β agonist RES decreased *Acbp*/*Dbi* expression and reversed metabolic signs of Cushing’s syndrome.

## Discussion

Classical and tissue hormones as well as neurotransmitters are long- or short-distance messengers embedded in complex communication networks^30^. For this reason, we were intrigued by the possibility that ACBP/DBI would be connected to other neuroendocrine factors. In our screen (which was based on neuroblastoma and hepatoma cells), we found two other neuroendocrine factors, (1) glucocorticoids and (2) thyroid hormone, that negatively affected the cellular content of ACBP/DBI, although due to rather distinct mechanisms. Glucocorticoid receptor agonists enhanced autophagy and increased *ACBP*/*DBI* mRNA, suggesting that they induce the autophagy-dependent release of ACBP/DBI, which indeed accumulated in the supernatant of neuroblastoma or hepatoma cells. These findings echo a previous report demonstrating that glucocorticoids cause astrocytes to release ACBP/DBI protein into the supernatant^13^. In accordance with the in vitro results, mice treated with glucocorticoids showed an increase in circulating ACBP/DBI protein levels, and patients under corticotherapy or with endogenous Cushing’s syndrome also exhibited elevated ACBP/DBI levels. In contrast, T3 reduced *ACBP*/*DBI* mRNA without autophagy and without increasing extracellular ACBP/DBI in cell cultures or in the plasma of mice. Accordingly, T3 administration could block the corticotherapy-induced surge in ACBP/DBI plasma concentrations.

To demonstrate that ACBP/DBI is involved in Cushing’s syndrome, we used six different approaches for ACBP/DBI inhibition: (1) induction of ACBP/DBI neutralizing antibodies, (2) i.p. injection of a neutralizing mAb, (3) conditional whole-body or hepatocyte-specific knockout of the *Dbi* gene, (4) mutation of the ACBP/DBI receptor (*Gabrg2*^*F77I/F77I*^) and (5) treatment with T3 or (6) RES to block *Dbi* transcription. These six methods convergently abolished important facets of CORT-induced Cushing’s syndrome, particularly increased food intake, weight gain, adiposity affecting all WAT subtypes and iBAT with hypertrophy of adipocytes, dyslipidaemia with increased TG and FFA, and of T2D such as hyperinsulinaemia and insulin resistance. Thus, surprisingly, these major characteristics of Cushing’s syndrome are not directly mediated by the action of glucocorticoids on metabolically active cell types (such as adipocytes, hepatocytes and muscle cells) but apparently require the obligatory contribution of extracellular ACBP/DBI (that can be inhibited by neutralizing antibodies) acting on GABA_A_R receptors containing the GABRG2 subunit. These GABA_A_R receptors are indeed expressed by multiple metabolically active cell types outside of the central nervous system^11,40^. Since ACBP/DBI does not cross the blood–brain barrier^18^, it appears plausible that the metabolic effects of antibody-mediated neutralization of ACBP/DBI are mediated by such peripheral receptors. Other effects of Cushing’s syndrome such as adrenal and thymic atrophy, as well as a reduction in skeletal muscle mass, were less susceptible to ACBP/DBI inhibition, in line with reported direct effects of glucocorticoids on thymocytes^41^, on the hypothalamic–hypophyseal axis producing ACTH^42^ and on skeleton muscle cells^43^.

It is possible that some of the metabolic effects of glucocorticoids are mediated through additional neuroendocrine factors, as this has previously been demonstrated for endocannabinoids^44^. Indeed, although ACBP/DBI supplementation alone (without glucocorticoids) did induce an increase in adiposity and weight gain, it failed to induce a full Cushing’s phenotype that would include a reduction of lean mass with sarcopenia. Thus, ACBP/DBI supplementation alone did not significantly modulate lean mass and actually attenuated the loss of lean mass induced by chronic restraint stress or chemotherapy in mouse models of anorexia^18^. Similarly, ACBP/DBI supplementation alone tended to increase the cross-sectional diameter of skeleton muscle fibres and prevented the atrophy of muscle fibres induced by chemotherapy^18^. Chronic restraint stress, which causes anorexia in mice, leads to an increase of circulating glucocorticoid concentrations^18^, and this elevation is also observed in human anorexia nervosa^45^. In mice under chronic restraint stress, the elevation in CORT plasma levels was attenuated by ACBP/DBI supplementation^18^. Of note, neither glucocorticoids nor glucocorticoid antagonists are effective against human anorexia nervosa^46,47^. The two diseases, Cushing’s syndrome and anorexia nervosa, are completely different nosological entities. Altogether, these findings suggest that ACBP is necessary but not sufficient for the induction of Cushing’s syndrome. Moreover, at this point, it is not clear whether the anti-Cushing’s effects of T3 and RES are entirely due to ACBP/DBI downregulation or whether they involve additional effects mediated by THR-β.

It is noteworthy that ACBP/DBI neutralization apparently has appetite and weight gain-inhibitory effects. Thus, as shown here, ACBP/DBI inhibition prevented hyperphagia and adiposity induced by glucocorticoids but not by citalopram. We reported in the past that ACBP/DBI neutralization suppressed hyperphagia induced by previous food deprivation or the thiazolidinedione antidiabetic rosiglitazone but not by the injection of ghrelin^11,14^. Of note glucocorticoids, rosiglitazone and starvation increase circulating ACBP/DBI levels, while citalopram and ghrelin fail to do so^11,14^. These findings suggest that, in a heterogeneous population of individuals with overweight or obesity, elevated plasma ACBP/DBI concentrations might constitute a biomarker that predicts sensitivity to ACBP/DBI inhibition. Nonetheless, this conjecture requires further scrutiny in suitable pre-clinical models and future clinical trials. Moreover, pair-feeding experiments in which caloric intake (and, as a result, body weight) are kept constant indicate that glucocorticoids can induce signs of metabolic syndrome (such as dyslipidaemia and insulin resistance) in the absence of weight gain and that ACBP/DBI neutralization reverses such effects independently from its effects on food intake.

At a more speculative level, it appears that ACBP/DBI, the (hypothetically) oldest protein hormone^7,29^, is controlled by two phylogenetically younger non-peptide hormones, namely, thyroid hormone (that is produced by protochordates and vertebrates)^48^ and glucocorticoids (found only in vertebrates)^49^. Thus, in a possible scenario, ACBP/DBI might have been placed under the control of these hormones during (pre-)vertebrate evolution. While glucocorticoids increase circulating ACBP/DBI levels, inhibition of ACBP/DBI does not affect the level of circulating endogenous glucocorticoids in unstressed mice, indicating a clearly hierarchical relationship without feedback loops. Future work must understand through which (direct or indirect) mechanisms ACBP/DBI controls other neuroendocrine factors, including those involved in metabolic regulation (such as insulin and leptin), and when this control has been established during vertebrate evolution.

Irrespective of these uncertainties, it appears that a glucocorticoid-induced increase in extracellular (plasma) ACBP/DBI levels is mechanistically involved in the phenotypic manifestation of several signs of Cushing’s syndrome including increased appetite, weight gain, excessive adiposity, liver damage, dyslipidaemia and T2D. These results obtained in a murine model of Cushing’s syndrome require future clinical validation.

## Methods

### Ethics statement

All patients gave written informed consent, and study protocols were approved by the local ethics committees. The study of cohort I was approved by the Institutional Review Board of Dermatology and Hospital for Skin Diseases and the Ethics Committee of Suzhou Institute of Systems Medicine, Chinese Academy of Medical Sciences and Peking Union Medical College with the approval (2021) Linkuaishen (005) and (2023) Lunshen015, respectively. The study protocol for cohort II was approved by Marseille, Comité de Protection des Personnes, Sud Méditerranée II (identification 2014-A01302-45 and 2016-A00026-45; clinical trial identification: NCT02335996 and NCT02848703). All animal experimentation procedures applied to institutional rules and guidelines and were approved by the Gustave Roussy ethics committee (project numbers 2023_011_40501, 2023_053_44146 and 2024_040_50288).

### Cell culture and reagents

Human neuroglioma H4 cells wild type (cat. no. HTB-148, ATCC) or stably expressing GFP–LC3 were cultured in Dulbecco’s modified Eagle medium (cat. no. 11995065, Thermo Fisher Scientific), supplemented with 10% (v/v) foetal bovine serum (cat. no. A5256701, Thermo Fisher Scientific), 100 U ml^−1^ penicillin and 100 μg ml^−1^ streptomycin (cat. no. 15140122, Thermo Fisher Scientific) at 37 °C in a humidified atmosphere with 5% CO_2_. Human hepatocellular carcinoma HepG2 cells wild type (cat. no. HB-8065, ATCC) or stably expressing GFP–LC3 were cultured in Eagle’s Minimum Essential Medium (cat. no. 30-2003, ATCC) supplemented as above. Plastic material was from Corning. Dialysed foetal bovine serum (cat. no. F0392) was from Thermo Fisher Scientific.

### High-throughput screening

Two thousand H4 GFP–LC3 cells per well were seeded in 384-well µClear imaging plates (cat. no. 781091, Greiner Bio-One) 24 h before experimentation. Following compound treatment (Supplementary Table 1), cells were fixed with 4% paraformaldehyde containing 10 mM Hoechst 33342 (cat. no. H3570, Thermo Fisher Scientific) in phosphate-buffered saline (PBS) for 30 min at room temperature (RT), permeabilized with 0.1% Triton X-100 for 10 min and blocked with 5% bovine serum albumin (BSA) in PBS for 1–2 h, followed by overnight incubation with anti-ACBP antibody (cat. no. sc-376853, Santa Cruz Biotechnology) at 4 °C. After 2× washing with PBS, cells were incubated with AlexaFluor-conjugated secondary antibody (cat. no. A-11004; Thermo Fisher Scientific) for 2 h at RT. Cells were washed 3× before acquisition using an ImageXpressMicroXL bioimager (Molecular Devices) equipped with a 20× PlanApo objective (Nikon). Subsequently, images were analysed with R software (http://www.r-project.org/) using EBImage (https://bioconductor.org/) and MetaxpR (https://github.com/asauvat/MetaxpR) packages. Viability assessment, data processing and statistical evaluation were conducted as described in ref. ^50^.

### Human cohort

Ninety-five dermatology patients with skin disease were included in cohort I (median age 46.8 years, range 11–93 years) (Supplementary Table 3). Subjects in the treatment group (*n* = 56) received glucocorticoid therapy, and patients (*n* = 3) who developed resistance to synthetic glucocorticoids were excluded. For cohort II (Supplementary Table 4), patients with ACTH-dependent Cushing’s syndrome (21 with ACTH-dependent pituitary Cushing’s syndrome, that is, Cushing’s disease, and 3 with ectopic ACTH secretion due to a bronchial carcinoid) (median age 56.5 years, range 22–73 years) were prospectively recruited from 2014 to 2017 at Marseille University Hospital, France. The ‘active’ group consisted of 11 newly diagnosed patients. The ‘remission’ group consisted of 13 patients in remission for at least 2 years, but no more than 6 years, regardless of the treatment modality.

#### Animal experimentation

Six-week-old female and male C57BL/6J mice (Envigo) were group-housed and subjected to a 12 h light–dark cycle, under temperature-controlled specific pathogen-free (SPF) conditions with food and water ad libitum. Food intake per day per mouse was calculated by measuring chow weight semiweekly. Mice were kept for 1 week to acclimate upon arrival before commencing experiments. For ACBP/DBI autoimmunization, autoantibody production via active immunization was initiated by conjugating keyhole limpet haemocyanin (KLH, cat. no. 77649, Thermo Fisher Scientific) and mouse recombinant ACBP/DBI (KLH–ACBP) as described^34^. Wild-type 6-week-old female C57BL/6J mice were immunized via i.p. injections of 30, 30, 30 and 10 μg of KLH–ACBP, or KLH alone, emulsified (1:1) with Montanide ISA-51vg (cat. no. 36362/FL2R3, Seppic) on days 0, 7, 14 and 21, respectively. After 4 weeks, ACBP autoantibodies were assessed by subjecting plasma to immunoblotting against recombinant target protein. For further validation enzyme-linked immunosorbent assay (ELISA) was used to assess circulating ACBP levels. Starting from week 6, Cushing’s syndrome was induced by administration for 5 weeks of CORT (cat. no. 27840, Merck) dissolved in EtOH, at 100 μg ml^−1^ in drinking water. Final EtOH concentration was 0.66% (ref. ^36^). Water consumption was measured semiweekly, and CORT concentration was adjusted to maintain an average daily CORT exposure of approximately 500 μg per mouse. The control group received 0.66% vehicle in drinking water. C57BL/6 *Gabrg2*^*tm1Wul*^/J *Gabra* flox mice came from the Jackson Laboratory. All mice used for experimentation were female. C57BL/6J Acbp^*fl/fl*^ with loxP sites flanking *Acbp* exon 2 was generated by Ozgene. To activate the CRE recombinase, tamoxifen was administered i.p. at a dosage of 75 mg kg^−1^ body weight per mouse per day for five consecutive days. Tamoxifen was diluted in 90% corn oil and 10% EtOH (v/v) at a concentration of 20 mg ml^−1^ and was agitated overnight at 37 °C. After tamoxifen administration, mice underwent a washout period of at least 1 week before the commencement of treatments. This knockout strategy was applied in combination with the expression of either a ubiquitous or hepatocyte-specific CRE recombinase (genotypes: *UBC-cre-ERT2* or *TTR-creTam*, respectively). Following the procedure, mice were kept for at least a week before starting the treatment. For the neutralization of DBI by anti-ACBP mAb, experiments were conducted with 8-week-old female C57BL/6J mice. Passive immunization was performed by semiweekly i.p. injections of 5 mg kg^−1^ body weight anti-ACBP mAb (clone 7G4a, homemade) or isotype control (IgG2a, κ, cat. no. BE0085, Bio X Cell). For RES treatment, experiments were conducted with 8-week-old female C57BL/6J mice. RES (cat. no. HY-12216, MedChemExpress) was prepared in 10% dimethyl sulfoxide (DMSO), 40% PEG300, 5% Tween 80 and 45% drinking water (v/v), at a final concentration of 0.033 mg ml^−1^. For the T3 system, experiments were conducted with 8-week-old female C57BL/6J mice. The initial concentration of T3 (cat. no. T2877, Merck) was 3.3 μg ml^−1^and was adjusted to maintain an average daily exposure of approximately 10 μg per mouse. For citalopram treatment, experiments were conducted with 8-week-old female C57BL/6J mice. Citalopram (Seropram, Lundbeck) was administered at a concentration of 0.15 mg ml^−1^ (diluted in water). For all experiments described above, CORT was administered as described above. Water consumption, body weight and food intake were measured semiweekly. At the end of the fifth week, mice were killed, and tissues were collected and weighed.

#### Co-administration of DEX and mifepristone

Experiments were conducted with 8-week-old female C57BL/6J mice. DEX (cat. no. D4902, Merck) was diluted in 10% DMSO and 90% corn oil (v/v) and administered i.p. (5 mg kg^−1^ body weight). Mifepristone (cat. no. M8046, Merck) was diluted in drinking water containing 1% carboxymethyl cellulose (cat. no. 419281, Merck) with 0.20% Tween 80 (Cat. P4780, Merck) (v/v) and administered by oral gavage (120 mg kg^−1^ body weight). DEX was injected daily for 2 weeks. Mifepristone was administered from day 7 to day 14. For SAFit2 and fasting, experiments were conducted with 8-week-old female C57BL/6J mice. SAFit2 (cat. no. HY102080 MedChemExpress) was solubilized in vehicle (4% EtOH, 5% Tween80, and 5% PEG400 (v/v) in 0.9% saline (Veh-1)). CORT was dissolved in 100% EtOH, to a final EtOH concentration of 0.66% (Veh-2). Fasting was performed by removing food for 24 h. SAFit2 was injected i.p. at 40 mg kg^−1^per day, and CORT (500 µg per mouse) was given by oral gavage. Daily CORT exposure was adjusted to approximately 500 µg per mouse.

#### Pair feeding

C57BL/6J female mice were housed under standard conditions with a 12 h light–dark cycle and ad libitum access to water. Mice were randomly assigned to four treatment groups: vehicle, CORT, anti-ACBP/DBI antibody and CORT plus anti-ACBP/DBI antibody. To ensure controlled food intake, a pair-feeding protocol was implemented. Initially, baseline body weights and food consumption were measured over a 3-day period to establish average intake. The vehicle-treated group served as the control for food intake. The average daily food intake of the vehicle-treated group was calculated and used to determine the amount of food provided to the other groups. Mice in the CORT, anti-ACBP/DBI antibody and CORT plus anti-ACBP/DBI antibody groups were given the same amount of food consumed by the control group on the previous day. Food intake and body weights were recorded daily to ensure precise matching of food quantities across groups. Adjustments in food allocation were made on the basis of the control groups’ consumption. Daily CORT exposure was adjusted to approximately 500 µg per mouse.

### Indirect calorimetry measurements

Indirect calorimetry was conducted using automated metabolic cages (Labmaster, TSE Systems GmbH), in which mice were individually housed for consecutive 7-day periods over 4 weeks. Each cage was equipped with bedding, and mice were provided unrestricted access to food and water. Food and water consumption was continuously monitored. Measurements included oxygen (O_2_) consumption, carbon dioxide (CO_2_) production, the respiratory exchange rate (RER = VCO_2_/VO_2_) and heat production. Locomotor activity, including ambulatory and fine movements as well as speed, was tracked using an infrared light beam-based system. O_2_ and CO_2_ volumes were assessed at the inlet ports of each cage and periodically calibrated against a reference empty cage. All measurements were conducted at 4-min intervals throughout the experiment, ensuring continuous recording during both light and dark phases.

### Forced swim test

For the forced swim test (FST), mice were placed in a vertical glass cylinder filled with water and behaviour was observed for 5 min. Water temperature was maintained at 25 °C. Distinct phases of active swimming and immobility were documented. The time spent immobile during the test was considered an indicator of behavioural despair. Conversely, less time spent immobile suggested potential antidepressant effects.

### Face angle assessment

Mice were anaesthetized and placed on a scaled matrix with a protractor. Bird’s-eye-view images were taken and then analysed to measure the angle between the edges of the two cheeks considering the tip of the nose as the vertex.

### Immunoblot

For protein extraction, cells were washed twice with with PBS and collected in radioimmunoprecipitation assay buffer (cat. no. 89901, Thermo Fisher Scientific), subjected to ultrasonication for three pulses of 10 s on ice and then centrifuged for 10 min at 13,000*g*. Analogously 30 μg liver tissues and 60 μg adipose tissue were collected in Precellys lysing kits (cat. no. P000911-LYSK0-A, Bertin Technologies) with radioimmunoprecipitation assay buffer and protease/phosphatase inhibitors (cat. no. A32959, Thermo Fisher Scientific), followed by two cycles of homogenization for 20 s at 5,500 rpm using a Precellys homogenizer (Bertin Technologies). Then, samples were centrifuged at 13,000*g* for 30 min and supernatants were collected. The Bio-Rad BCA assay (DC Protein Assay Kit II, cat. no. 5000112, Bio-Rad) was used for protein concentration assessment. Loading buffer and reducing agent (cat. nos. NP0008 and NP0009, Thermo Fisher Scientific) were added before denaturation (100 °C for 15 min). After SDS–PAGE and electrotransfer to polyvinylidene fluoride membranes, unspecific binding sites were blocked for 1–2 h with 5% BSA at RT, followed by overnight incubation at 4 °C with primary anti-human ACBP/DBI antibody (1:500; cat. no. sc-376853, Santa Cruz Biotechnology), anti-mouse ACBP/DBI antibody (1:1,000; cat. no. ab231910, Abcam), anti-LC3B antibody (1:1,000; cat. no. ab192890, Abcam), anti-SQSTM1/p62 antibody (1:1,000; cat. no. ab109012, Abcam), anti-glucocorticoid receptor (D6H2L) XP antibody (1:1,000; cat. no. 12041, Cell Signaling Technology) or horseradish peroxidase (HRP)-coupled anti-β-actin antibody (1:2,000; cat. no. ab49900, Abcam). Membranes were washed and processed by incubation with HRP-coupled secondary antibody (1:2,000; cat. no. 4050-05 goat anti-rabbit IgG(H + L), mouse/human ads-HRP and cat. no. 1031-05 goat anti-mouse IgG(H + L), human ads-HRP, SouthernBiotech) for 1 h at RT. Imaging and quantification were conducted by using an ImageQuant LAS4000 and ImageJ software, respectively.

### Mouse and human ACBP/DBI ELISA

Cells were treated and culture supernatants were collected, centrifuged at 500*g* for 5 min and stored at −80 °C until analysis. For in vivo experiments, mouse plasma was collected with lithium heparin separator (cat. no. 450535, Greiner Bio-One), then centrifuged at 1,500*g* for 10 min, and ACBP/DBI levels were measured by ELISA. Human anti-ACBP/DBI (cat. no. MBS768488, MyBioSource) and murine anti-ACBP/DBI capture antibodies (cat. no. ab231910, Abcam) diluted 1 µg ml^−1^ in PBS were used for coating high-binding 96-well plates (Corning) with 100 μl per well overnight at 4 °C. Subsequently, plates were washed twice with washing solution (0.05% Tween 20 (v/v) in PBS), and unspecific binding was blocked with 100 μl sterile blocking buffer (1% BSA and 0.05% Tween 20 (v/v) in PBS) for 2 h at RT. For sample assessment, 100 μl per well of either sample or standard (human serum at 1:50–1:75, murine plasma at 1:20 and cell culture supernatant at 1:4 dilution, with the flexibility to adjust as dictated by experimental requirements) was incubated for 2 h at RT and subsequently rinsed 3× with washing buffer. Then 100 μl per well human anti-ACBP/DBI (LS-C299614, Lifespan Biosciences) and murine anti-ACBP/DBI detection antibodies (cat. no. MBS2005521, MyBioSource) diluted 1 µg ml^−1^ in PBS were added for 1 h at RT followed by 3× rinsing with washing buffer. Subsequently, plates were incubated 30 min at RT with 100 μl of HRP-coupled avidin diluted in PBS (1/5,000 for human and 1/1,000 for murine samples). Subsequently plates were rinsed 4× with washing buffer. To visualize bound protein, 100 μl of 1-Step Ultra TMB-ELISA substrate solution (cat. no. 34029, Thermo Fisher Scientific) was added and incubated 10–30 min at RT in the dark. Then, 50 μl of stop solution (2 N H_2_SO_4_) was added and absorbance was measured at 450 nm using a FLUOstar OPTIMA microplate reader.

### RNA interference

Small interfering RNAs (siRNAs) were purchased from Horizon Discovery (ON-TARGETplus, GE Healthcare Dharmacon) and used following the manufacturer’s protocol and employing the following siRNA oligos:

*siCtr*, 5′-UAGCGACUAAACACAUCAA-3′;

*siNR3C1*-#1, 5′-GAACUUCCCUGGUCGAACA-3′;

*siNR3C1*-#2, 5′-GCAUGUACGACCAAUGUAA-3′;

*siATG5*, 5′-GGCAUUAUCCAAUUGGUUU-3′;

*siATG7*, 5′-CCAACACACUCGAGUCUUU-3′;

*siTHRA*, 5′-GUAUAUCCCUAGUUACCUG-3′;

*siTHRB*, 5′-GGACAAGCACCAAUAGUCA-3′.

### Biochemical assays

ELISA kits were used for detecting biochemical indices, such as a mouse ALT ELISA kit (cat. no. ab282882, Abcam), mouse AST ELISA kit (cat. no. ab263882, Abcam), mouse insulin ELISA kit (cat. no. 10-1247-01, Mercodia), TG assay kit (cat. no. ab65336, Abcam), FFA assay kit (cat. no. ab65341, Abcam) and plasma CORT ELISA kit (cat. no. ab108821, Abcam). Plasma CORT samples were collected during the first hour of light at 8:00, and the collection process was performed under general anaesthesia using isoflurane inhalation. All procedures strictly followed the manufacturer’s protocol. For Luminex multiplex assays, plasma was collected in an EDTA anti-coagulant collecting tube with additional dipeptidyl peptidase IV inhibitor, protease inhibitor cocktail, aprotinin and serine protease inhibitor (Merck), then centrifuged for 10 min at 1,000*g* within 30 min of collection, aliquoted and stored at −80 °C. Mouse hormones were detected by using the mouse metabolic hormone magnetic bead panel (cat. no. MMHMAG-44K, Merck) and adiponectin single kit (cat. no. MADPNMAG-70K-01, Merck) by Luminex following the manufacturer’s protocol.

### GTT and ITT

Mice were trained for tail pinch adaptation 1 week in advance. Animals were fasted for 6 h to perform a GTT. Blood for glycaemia measurement was collected from tail vein incisions 0, 15, 30, 60, 90 and 120 min after the injection of d-glucose (2 g kg^−1^ body weight, i.p.; cat. no. D3179, Merck). For ITT, animals were fasted 2–4 h before injection of insulin (NovoRapid, 0.5 U kg^−1^ body weight, i.p.). Blood was collected from tail cuts at 0, 15, 30, 60, 90 and 120 min, and glucose was measured using a precision glucometer (Accu-Chek Performa). Mice were monitored frequently, and hypoglycaemic shock was avoided by administration of 20% glucose solution. The HOMA-IR is calculated using the following formula: fasting plasma glucose (measured after 16 h of fasting, in millimolar) multiplied by fasting plasma insulin (measured after 16 h of fasting, in microunits per litre), divided by 22.5.

### Histopathology

Fresh tissue was collected and fixed in 4% paraformaldehyde (or 10% formalin) for a maximum of 24 h at RT, then processed by serial paraffin embedment. Ten-micrometre-thick slices were obtained with a microtome. Standard haematoxylin–eosin staining was performed, and slides were scanned by a semi-automatic slide scanner (Nanozoomer 2.0 HT, Hamamatsu) equipped with 20× and 40× objectives. The images were analysed and quantified using Fiji software.

### Gene expression analyses

For RNA extraction, the RNeasy Plus Mini kit (cat. no. 74134, QIAGEN) was used. About 25–30 mg of tissue was collected in lysis buffer (Buffer RLT Plus). The tissue was homogenized in two cycles using a Precellys homogenizer (Bertin Technologies) for 20 s at 5,500 rpm. The lysate was centrifuged and subjected to further purification procedures as described by the manufacturer. About 1 μg total RNA was reversed transcribed using the Maxima First Strand cDNA Synthesis Kit (cat. no. K1642, Thermo Fisher Scientific). Quantitative real-time PCR (qRT–PCR) was conducted by using PowerUp SYBR Green Master Mix (cat. no. A25776, Thermo Fisher Scientific) with a StepOnePlus Real-Time PCR System (Applied Biosystems, Thermo Fisher Scientific). The 2^−ΔΔCT^ method was used for the analysis of real-time PCR data with the following primers (Eurofins Scientific):

*ACBP*/*DBI* forward primer: CAGAGGAGGTTAGGCACCTTA;

*ACBP*/*DBI* reverse primer: TATGTCGCCCACAGTTGCTTG;

*GAPDH* forward primer: TGTGGGCATCAATGGATTTGG;

*GAPDH* reverse primer: ACACCATGTATTCCGGGTCAAT;

*Acbp*/*Dbi* forward primer: GCTTTCGGCATCCGTATCAC;

*Acbp*/*Dbi* reverse primer: ACATCGCCCACAGTAGCTTG;

*Gapdh* forward primer: CGACTTCAACAGCAACTCCCACTCTTCC;

*Gapdh* reverse primer: TGGGTGGTCCAGGGTTTCTTACTCCTT.

### Bulk RNA sequencing

RNA was isolated from murine liver tissue according to the manufacturer’s protocol (cat. no. 217004, miRNeasy mini kit, QIAGEN). Sequencing was conducted on a NovaSeq 6000 PE150 instrument, yielding paired-end reads of 2 × 150 base pairs, with a total of 40 million reads per sample (Novogene). Alignment and mapping to the GRCm39 (mm39) genome assembly were accomplished using HISAT2 (version 2.2.1). The resulting SAM file was processed by HTSeq-count (version 2.0.2) utilizing the union mode and including non-unique features for the generation of gene count tables. Software and websites used for analysis were RStudio (version 4.3.1), Cytoscape and https://string-db.org/.

### Differential gene expression analysis

For gene expression comparison, volcano plots using the Enhanced Volcano R package, Venn diagrams using the Venn R package and heatmaps using the Complex Heatmap R package were generated. Differentially expressed genes (*P* ≤ 0.05 and |log_2_(fold change)| ≥1.0) were selected and further assessed by functional enrichment analysis, using various R packages, including clusterProfiler (v4.8.2^51^), tidyverse, ggplot, forcats, biomaRt, stringr and org.Mm.eg.db. The gene background was defined using all sequenced genes.

### Liver sample preparation and metabolite analysis

Approximately 30 mg of liver sample was homogenized as previously described^52^. For the extraction of endogenous metabolites, samples were mixed with 1 ml of ice-cold 90% methanol, 10% water (v/v) at −20 °C, along with a cocktail of internal standards and homogenized using a Precellys tissue homogenizer (Bertin Technologies), applying 3 cycles of 20 s at 6,500 rpm. After centrifugation (10 min at 15,000*g*, 4 °C), supernatants were divided into fractions and processed following established protocols. One fraction was used for short-chain fatty acid analysis (derivatization before injection). Another fraction was allocated to liquid chromatography–mass spectrometry (MS) analysis, while the third fraction was used for gas chromatography (GC)–MS analysis. Then, the analysis fractions were collected, dried (Techne DB3) at 40 °C and subsequently kept at −80 °C. Widely targeted analysis was conducted using a 7890A GC system (Agilent Technologies) coupled to a QQQ 7000C triple quadrupole mass spectrometer (Agilent Technologies) for GC–MS/MS. For the analysis of polyamines, short-chain fatty acids and bile acids, liquid chromatography-MS/MS was used utilizing a 1290 UHPLC system (Agilent Technologies) coupled to a QQQ 6470 triple quadrupole mass spectrometer (Agilent Technologies). Furthermore, a pseudo-targeted analysis was performed using an ultra-high performance liquid chromatography-high-resolution mass spectrometry system (UHPLC-HRMS), using a Dionex U3000 system coupled with an Orbitrap q-Exactive mass spectrometer (Thermo Fisher Scientific). All data were processed with the GRMeta in R (version 4.0) package (https://github.com/Kroemerlab/GRMeta). Data analysis and visualization were performed using AreaQCorrLog2Cen in R (version 4.2.1).

### Data analysis

Unless otherwise specified, data are presented as mean ± SEM Before conducting statistical analysis, normal distribution of the results was assessed using the D’Agostino and Pearson normality test, Shapiro–Wilk normality test and Kolmogorov–Smirnov test. For data that exhibited a Gaussian distribution, unpaired two-tailed Student’s *t*-test or one-way analysis of variance (ANOVA) or two-way ANOVA was used. In the case of data with non-Gaussian distributions, the Mann–Whitney *U* test was used for two-group comparisons, while the Kruskal–Wallis test followed by Dunn’s post hoc test was used for comparisons involving multiple groups. Body weight curves and food intake were longitudinally analysed with type II ANOVA and pairwise comparisons using the TumGrowth application (https://kroemerlab.shinyapps.io/TumGrowth/). All other statistical analyses were performed using GraphPad Prism 9 or R software.

### Reporting summary

Further information on research design is available in the Nature Portfolio Reporting Summary linked to this article.

## Supplementary information

Supplementary Information Supplementary figure legends, Supplementary Figs. 1–15 and Source data of immunoblots included in supplementary figures.

Reporting Summary

Supplementary Table 1 Supplementary Table 1.

Supplementary Tables 2–4 Supplementary Tables 2–4.

Supplementary Data 1 Source data of supplementary figures.

## Source data

Source Data Fig. 1 Numerical source data.

Source Data Fig. 1 Unprocessed western blots.

Source Data Fig. 2 Numerical source data.

Source Data Fig. 2 Unprocessed western blots.

Source Data Fig. 3 Numerical source data.

Source Data Fig. 4 Numerical source data.

Source Data Fig. 5 Numerical source data.

Source Data Fig. 6 Metabolic cage raw data.

Source Data Fig. 7 Numerical source data.

Source Data Fig. 8 Numerical source data.

Source Data Extended Data Fig./Table 1 Numerical source data.

Source Data Extended Data Fig. 1 Unprocessed western blots.

Source Data Extended Data Fig./Table 2 Numerical source data.

Source Data Extended Data Fig. 2 Unprocessed western blots.

Source Data Extended Data Fig./Table 3 Numerical source data.

Source Data Extended Data Fig./Table 4 Numerical source data.

Source Data Extended Data Fig./Table 5 Numerical source data.

Source Data Extended Data Fig./Table 6 Numerical source data.

Source Data Extended Data Fig./Table 7 Numerical source data.

Source Data Extended Data Fig./Table 8 Numerical source data.

Source Data Extended Data Fig./Table 9 Numerical source data.

Source Data Extended Data Fig. 9 Unprocessed western blots.

## Data Availability

The datasets generated during and/or analysed during the current study are annexed as Source data. RNA sequencing data are available at National Center for Biotechnology Information Gene Expression Omnibus database under the accession number GSE248672. Source data are provided with this paper.
