# Supplementary material for: Pathogenic role of acyl coenzyme A binding protein (ACBP) in Cushing’s syndrome
Source: Nat Metab. 2024 Nov 22;6(12):2281–99. doi: 10.1038/s42255-024-01170-0 (PMC11659162; doi:10.1038/s42255-024-01170-0)
Supplement: Supplementary file 1 — Supplementary figure legends, Supplementary Figs. 1–15 and Source data of immunoblots included in supplementary figures. [file 42255_2024_1170_MOESM1_ESM.pdf]

nature metabolism

Supplementary information https://doi.org/10.1038/s42255-024-01170-0

Pathogenic role of acyl coenzyme A binding protein (ACBP) in Cushing's syndrome

In the format provided by the authors and unedited

Additional (supplemental) information

Legends to Supplemental Figures

Fig. S1. Validation of hits in HepG2 GFP-LC3 cells. (**a-c**) Human hepatocellular carcinoma HepG2 cells stably expressing GFP-LC3 were treated with increasing doses of dexamethasone (DEX; 0.5, 1, 5 μM), hydrocortisone (HCS; 0.5, 1, 5 μM) or triiodothyronine (T3; 0.5, 1, 5 μM) for 24 h. Rapamycin (RAPA) was used as positive control. Scale bar equals 5 μm. Representative immunofluorescence images are shown in (**a**) and normalized ACBP fluorescent intensity (**b**) and normalized GFP-LC3 puncta formation (**c**) are shown in dot plots. Representative results of three technical replicates are shown. One-way ANOVA with Dunnett correction was used for statistical analysis (*P*-values are indicated). All dot plots depict means ± SD.

Fig S2. Dexamethasone induced ACBP/DBI secretion in a dose-dependent manner and its effects are blunted by mifepristone. (**a**) Scheme of administration of different doses of dexamethasone (DEX, 0, 5, 10 mg/kg) for 24 h in female C57BL/6J mice. (**b**) Scatter plot showing the relative weight of thymus after 24 h DEX treatment (*n=5/group*). Scatter plot showing plasma ACBP (**c**) and liver *Acbp/Dbi* mRNA level (**d**) after 24 h DEX treatment (*n=5/group*). (**e-h**) Representative immunoblot shows ACBP, LC3II/I, p62 level in liver after 24 h DEX treatment. Scatter plot showing the ratio of ACBP/β-actin (**f**), LC3II/LC3I (**g**), p62/β-actin (**h**) in liver respectively (randomly selected *n = 3/group*). (**I**) Representative immunoblot shows ACBP level in white adipose tissue (WAT) after 24 h DEX treatment. (**j**) Scatter plot showing the ratio of ACBP/β-actin in (**i**) (randomly selected *n=3/group*). (**k**) Scheme of the co-administration of DEX (5 mg/kg body weight, *i.p.*) and Mifepristone (Mif, 120 mg/kg body weight, oral gavage) in female C57BL/6J mice. (**l**) Representative immunoblot shows ACBP level in liver and WAT after co-administrated with DEX and Mif (randomly selected *n = 3/group*). (**m,n**) Scatter plot showing the ratio of ACBP/β-actin in liver (**m**) and in WAT (**n**) after co-administrated with DEX and Mif (randomly selected *n=3/group*). (**o**) Scatter plot showing the concentration of plasma ACBP of mice under different treatment (*n=6/group,* AU, arbitrary units). One-way ANOVA with Dunnett/Tukey correction was used for statistical analysis (*P*-values are indicated). All dot plots depict means ± SEM.

Fig S3. Original data corresponding to the heatmap in Fig. 2j showing corticosterone levels, organ weights, and biochemical parameters in the context of autoantibody-mediated neutralization of ACBP/DBI. (**a-r**) Female C57BL/6J mice were treated with corticosterone (CORT; 100 μg/mL or vehicle control (Ctrl) in drinking water, *p.o.*) for 5 weeks together with KLH-ACBP (for autoimmunization) or KLH alone both administered *i.p.* (*n=10 mice/group*). Mice were sacrificed and tissues were collected and weighed. Corticosterone levels are shown in (**a**). Scatter plots show the relative mass of liver (**b**), visceral fat (**c**), inguinal fat (**d**), perigonadal fat (**e**), interscapular brown adipose tissue (iBAT) (**f**), thymus (**g**), adrenal (**h**), erector spinae

(**i**) and gastrocnemius (**j**). Alanine aminotransferase (ALT) (**k**), aspartate aminotransferase (ALT) (**l**), triglycerides (TG) in plasma (**m**), TG in liver (**n**), free fatty acids in plasma (**o**), insulin (**p**), fasting glycemia (**q**) and HOMA-IR (**r**) are represented in scatter graphs. One-way ANOVA with FDR correction was used for statistical analysis (*P*-values are indicated). All dot plots depict means ± SEM.

Fig S4. Original data corresponding to the heatmap in Fig. 3f showing corticosterone levels, organ weights and biochemical parameters of conditional *Acbp/Dbi* whole-body knockout mice. (**a-p**) Female C57BL/6J mice (*Dbi*$^{-/-}$) or wild type controls (*Dbi*$^{+/+}$) were treated with corticosterone (CORT; 100 µg/mL or vehicle control (Ctrl) in drinking water, *p.o.*) for 5 weeks (*n=6 mice/group*). Mice were sacrificed and tissues were collected and weighed. Corticosterone levels are shown in (**a**). Scatter plots show the relative mass of liver (**b**), visceral fat (**c**), inguinal fat (**d**), perigonadal fat (**e**), interscapular brown adipose tissue (iBAT) (**f**), thymus (**g**), adrenal (**h**), erector spinae (**i**) and gastrocnemius (**j**). Triglycerides (TG) in plasma (**k**), TG in liver (**l**), free fatty acids in plasma (**m**), insulin (**n**), fasting glycemia (**o**) and HOMA-IR (**p**) are represented in scatter graphs. One-way ANOVA with FDR correction was used for statistical analysis (*P*-values are indicated). All dot plots depict means ± SEM.

Fig S5. Original data corresponding to the heatmap in Fig. 3o showing corticosterone level and organ weights in conditional hepatocyte-specific *Acbp/Dbi* knockout mice. (**a-g**) Female C57BL/6J mice (liver *Dbi*$^{-/-}$) or wild type controls (liver *Dbi*$^{+/+}$) were treated with corticosterone (CORT; 100 µg/mL or vehicle control (Ctrl) in drinking water, *p.o.*) for 5 weeks (*n=6 mice/group*). Mice were sacrificed and tissues were collected and weighed. Corticosterone levels are shown in (**a**). Triglycerides in liver (**b**), triglycerides in plasma (**c**), free fatty acids in plasma (**d**), insulin (**e**), fasting glycemia (**f**) and HOMA-IR (**g**) are represented in scatter graphs. One-way ANOVA with FDR correction was used for statistical analysis (*P*-values are indicated). All dot plots depict means ± SEM.

Fig S6. Original data corresponding to the heatmap in Fig. 4f showing of corticosterone levels, organ weights and biochemical parameters of *Gabrg2*$^{F77I/F77}$ mice. (**a-p**) Female C57BL/6J mice (*Gabrg2*$^{F77I/F77I}$) or wild type controls (*Gabrg2*$^{+/+}$) were treated with corticosterone (CORT; 100 µg/mL or vehicle control (Ctrl) in drinking water, *p.o.*) for 5 weeks (*n=6 mice/group*). Mice were sacrificed and tissues were collected and weighed. Corticosterone levels are shown in (**a**). Scatter plots show the relative mass of liver (**b**), visceral fat (**c**), inguinal fat (**d**), perigonadal fat (**e**), interscapular brown adipose tissue (iBAT) (**f**), thymus (**g**), adrenal (**h**), erector spinae (**i**) and gastrocnemius (**j**). Triglycerides (TG) in plasma (**k**), TG in liver (**l**), free fatty acids in plasma (**m**), insulin (**n**), fasting glycemia (**o**) and HOMA-IR (**p**) are represented in scatter graphs. One-way ANOVA with FDR correction was used for statistical analysis (*P*-values are indicated). All dot plots depict means ± SEM.

Fig S7. Original data corresponding to the heatmap in Fig. 5g showing

corticosterone levels, organ weights and biochemical parameters of mice treated with a neutralizing monoclonal αACBP/DBI antibody. (**a-r**) Female C57BL/6J mice were treated with corticosterone (CORT; 100 µg/mL or vehicle control (Ctrl) in drinking water, *p.o.*) for 5 weeks together with ACBP/DBI mAb (αACBP, 5 mg/kg body weight, *i.p.* semiweekly) or isotype control (*n=10,9,10,10/group*). Mice were sacrificed and tissues were collected and weighed. Corticosterone levels are shown in (**a**). Scatter plots show the relative mass of liver (**b**), visceral fat (**c**), inguinal fat (**d**), perigonadal fat (**e**), interscapular brown adipose tissue (iBAT) (**f**), thymus (**g**), adrenal (**h**), erector spinae (**i**) and gastrocnemius (**j**). Alanine aminotransferase (ALT) (**k**), aspartate aminotransferase (ALT) (**l**), triglycerides (TG) in plasma (**m**), TG in liver (**n**), free fatty acids in plasma (**o**), insulin (**p**), fasting glycemia (**q**) and HOMA-IR (**r**) are represented in scatter graphs (*n=10,9,10,10 mice/group*). One-way ANOVA with FDR correction was used for statistical analysis (*P*-values are indicated). All dot plots depict means ± SEM.

Fig S8. Liver cross-metabolite comparisons. Violin plots depicting distribution and probability density of liver metabolites (*n=28*, obtained from the Venn diagram in **Extended Fig. 8c**). All data were processed with the GRMeta in R (version 4.0) package (https://github.com/Kroemerlab/GRMeta). Data analysis and visualization were performed using AreaQCorrLog2Cen in R (version 4.2.1). In all inset boxplots, the center line represents the median, box limits represent upper and lower quartiles, and whiskers represent minimum and maximum values and points outside of this are considered outliers.. The width of the plot represents the data point density. Kruskal-Wallis test followed by Dunn's post hoc test was used for statistical analysis.

Fig S9. Plasma cross-metabolite comparisons. Violin plots depicting distribution and probability density of plasma metabolites (*n=28*, obtained from the Venn diagram in **Extended Fig. 8c**). All data were processed with the GRMeta in R (version 4.0) package (https://github.com/Kroemerlab/GRMeta). Data analysis and visualization were performed using AreaQCorrLog2Cen in R (version 4.2.1). In all inset boxplots, the center line represents the median, box limits represent upper and lower quartiles, and whiskers represent minimum and maximum values and points outside of this are considered outliers. The width of the plot represents the data point density. Kruskal-Wallis test followed by Dunn's post hoc test was used for statistical analysis.

Fig S10. Original data corresponding to the heatmap in Extended Fig. 9 showing corticosterone levels, organ weights and biochemical parameters of male mice receiving corticosterone together with a monoclonal antibody neutralizing ACBP/DBI. (**a-p**) Male C57BL/6J mice were treated with corticosterone (CORT; 100 µg/mL or vehicle control (Ctrl) in drinking water, *p.o.*) for 5 weeks together with ACBP/DBI mAb (αACBP, 5 mg/kg body weight, *i.p.* semiweekly) or isotype control (*n=10 mice/group*). Mice were sacrificed and tissues were collected and weighed. Corticosterone levels are shown in (**a**). Scatter plots depict the relative mass of liver (**b**), visceral fat (**c**), inguinal fat (**d**), perigonadal fat (**e**), interscapular brown adipose tissue

(iBAT) (**f**), thymus (**g**), adrenal (**h**), erector spinae (**i**) and gastrocnemius (**j**). Triglycerides (TG) in plasma (**k**), TG in liver (**l**), free fatty acids in plasma (**m**), insulin (**n**), fasting glycemia (**o**) and HOMA-IR (**p**) are represented as scatter graphs. One-way ANOVA with FDR correction was used for statistical analysis (*P*-values are indicated). All dot plots depict means ± SEM.

Fig S11. Original data corresponding to the heatmap in Fig. 7d showing corticosterone levels, organ weights, and biochemical parameters controls and pair-fed corticosterone-treated mice optionally receiving a monoclonal antibody neutralizing ACBP/DBI. Female C57BL/6J mice were treated with corticosterone (CORT; 100 μg/mL or vehicle control (Ctrl) in drinking water, *p.o.*) for 5 weeks together with ACBP/DBI mAb (αACBP, 5 mg/kg body weight, *i.p.* semiweekly) or isotype control (*n=10 mice/group*). Mice were divided into four groups: vehicle, corticosterone, anti-ACBP/DBI antibody, and corticosterone plus anti-ACBP/DBI antibody, and housed under standard conditions. Using a pair feeding protocol, the vehicle group's average daily food intake determined the food provided to other groups. Food intake and body weights were recorded daily, with adjustments based on the control group's consumption. Mice were sacrificed and tissues were collected and weighed. Corticosterone levels are shown in (**a**). The relative mass of fat and lean mass detected by nuclear magnetic resonance relaxometry are shown in (**b-c**). Scatter plots show the relative mass of liver (**d**), visceral fat (**e**), inguinal fat (**f**), perigonadal fat (**g**), interscapular brown adipose tissue (iBAT) (**h**), thymus (**i**), adrenal (**j**), erector spinae (**k**) and gastrocnemius (**l**). Triglycerides (TG) in plasma (**m**), TG in liver (**n**), free fatty acids in plasma (**o**), insulin (**p**), fasting glycemia (**q**) and HOMA-IR (**r**) are represented in scatter graphs (*n=10 mice/group*). One-way ANOVA with FDR correction was used for statistical analysis (*P*-values are indicated). All dot plots depict means ± SEM.

Fig. S12. Citalopram effects on ACBP/DBI. (**a**) Scheme showing passive immunization of female C57BL/6J mice with ACBP/DBI mAb (αACBP, 5 mg/kg body weight, injection *i.p.* semiweekly) and co-administrated with citalopram (CTP, 0.15 mg/mL or vehicle control (Ctrl) in drinking water, *p.o.*) for 8 weeks. Isotype (5 mg/kg body weight, injection *i.p.* semiweekly) was used as control (**b**) Average food intake (*n=3 cages/group*) and (**c**) body weight (*n=10 mice/group*) were measured weekly in different treatment groups. *P*-value depicts the comparison of areas under the curve. (**d**) Scheme showing mice administrated with corticosterone (CORT, 100 μg/mL solution, *p.o.*) or citalopram (CTP, 0.15 mg/mL in drinking water, *p.o.*) for 24 h in female C57BL/6J mice. (**e**) Plasma ACBP level under CORT and CTP treatments (*n=6/group*). (**f**) Representative immunoblot shows ACBP level in liver treated with CORT or CTP (randomly selected *n=3/group*). β-actin was used as a loading control. (**g**) Scatter plot showing the ratio of ACBP/β-actin. (**h**) Representative immunoblot shows ACBP level in white adipose tissue (WAT) treated with CORT or CTP (randomly selected *n=3/group*). β-actin was used as a loading control. (**i**) Scatter plot showing the ratio of ACBP/β-actin. One-way ANOVA with Tukey correction was used for statistical analysis (*P*-values are indicated). All dot plots depict means ± SEM. All curves were

longitudinally analyzed with type II ANOVA and pairwise comparisons.

Fig. S13. Original data corresponding to the heatmap in Extended Fig. 9 showing corticosterone levels, organ weights, and biochemical parameters of mice treated with corticosterone alone or in combination with triiodothyronine (T3). Female C57BL/6J mice were treated with corticosterone (CORT; 100 µg/mL or vehicle control (Ctrl) in drinking water, *p.o.*) together with T3 (T3, 3.3 µg/mL, drinking water, *p.o.*) for 5 weeks (*n=10 mice/group*). Mice were sacrificed and tissues were collected and weighed. Corticosterone levels are shown in (**a**). Scatter plots show the relative mass of liver (**b**), visceral fat (**c**), inguinal fat (**d**), perigonadal fat (**e**), interscapular brown adipose tissue (iBAT) (**f**), thymus (**g**), adrenal (**h**), erector spinae (**i**) and gastrocnemius (**j**). Triglycerides (TG) in plasma (**k**), TG in liver (**l**), free fatty acids in plasma (**m**), insulin (**n**), fasting glycemia (**o**) and HOMA-IR (**p**) are represented in scatter graphs. One-way ANOVA with FDR correction was used for statistical analysis (*P*-values are indicated). All dot plots depict means ± SEM.

Fig S14. Effects of short-term RES administration on ACBP/DBI expression. (**a,b**) Human hepatocellular carcinoma HepG2 wildtype cells were treated with siRNA targeting the thyroid hormone receptor (*THR*) β or control siRNA (*siCtrl*) with or without the resmetirom (RES, 0.3 µM) treatment. Upon knockdown cells were treated as indicated (*n=3/group;* RU, relative units). *THRβ* mRNA level (**a**) and ACBP mRNA level (**b**) were measured. (**c**) Normalized ACBP fluorescent intensity of HepG2 cells treated with 0.3 µM of different thyroid hormone receptor agonists or antagonists for 24 h (*n=4/group;* AU, arbitrary units; means±SD). Rapamycin (RAPA) was used as a positive control. One-way ANOVA with Dunnett/Tukey correction was used for statistical analysis (*P*-value*s* are indicated). All dot plots depict means ± SEM. *P*-values are shown compared to Ctrl.

Fig S15. Original data corresponding to the heatmap in Extended Fig. 8i showing corticosterone levels, organ weights, and biochemical parameters of mice treated with corticosterone alone or in combination with resmetirom. Female C57BL/6J mice were treated with corticosterone (CORT; 100 µg/mL or vehicle control (Ctrl) in drinking water, *p.o.*) together with resmetirom (RES, 0.033 mg/mL, drinking water, *p.o.*) for 5 weeks (*n=9,10,10,10 mice/group*). Mice were sacrificed and tissues were collected and weighed. Corticosterone levels are shown in (**a**). Scatter plots show the relative mass of liver (**b**), visceral fat (**c**), inguinal fat (**d**), perigonadal fat (**e**), interscapular brown adipose tissue (iBAT) (**f**), thymus (**g**), adrenal (**h**), erector spinae (**i**) and gastrocnemius (**j**). Triglycerides (TG) in plasma (**k**), TG in liver (**l**), free fatty acids in plasma (**m**), insulin (**n**), fasting glycemia (**o**) and HOMA-IR (**p**) are represented in scatter graphs (*n=9,10,10,10 mice/group*). One-way ANOVA with FDR correction was used for statistical analysis (*P*-values are indicated). All dot plots depict means ± SEM.

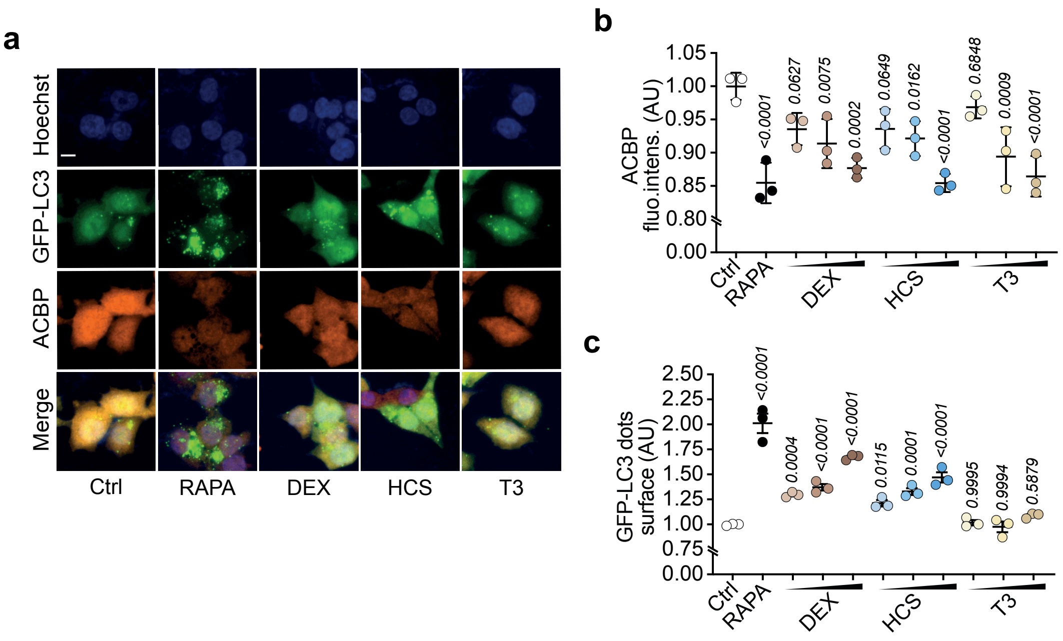

Supplemental Fig. 1

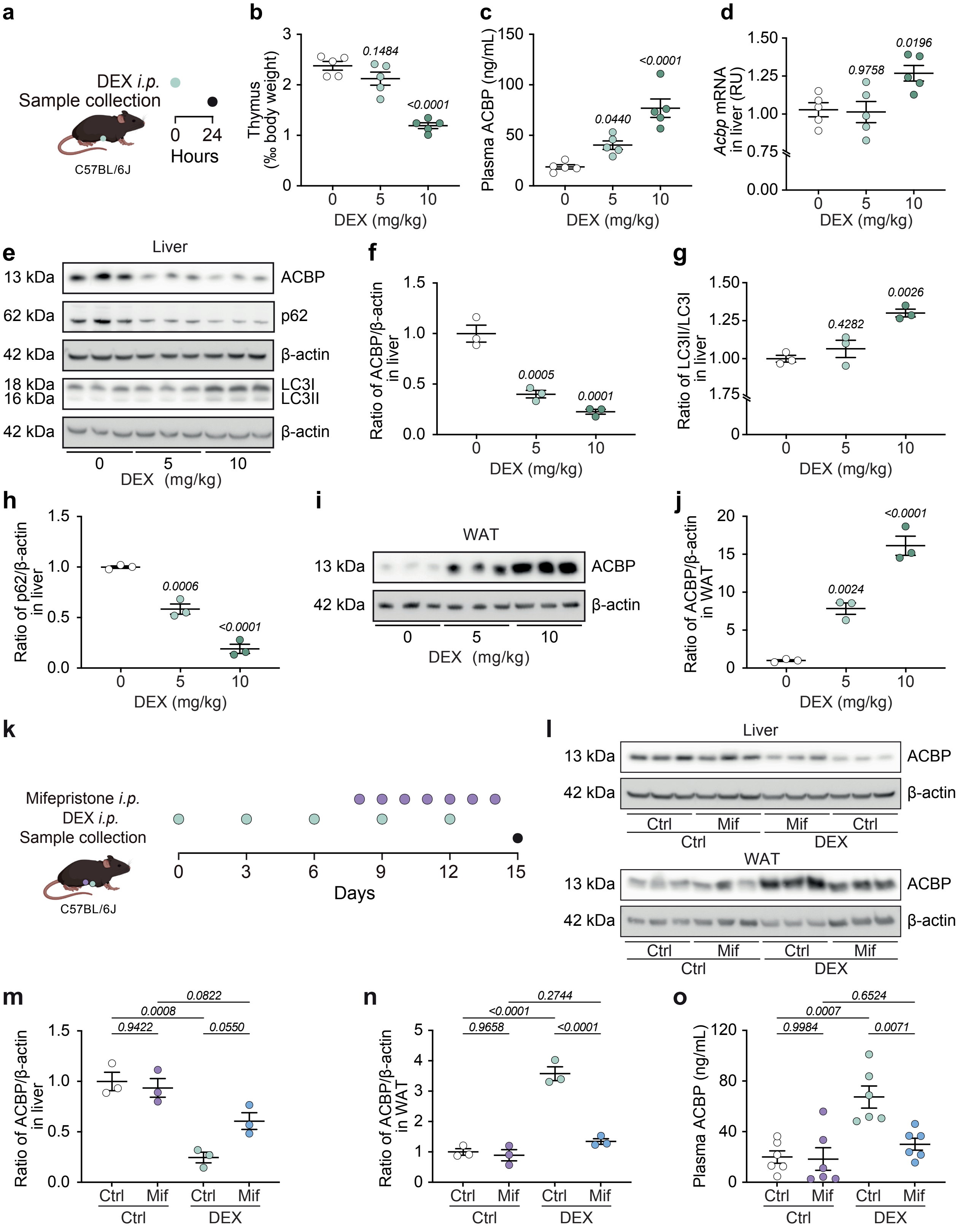

Supplemental Fig. 2

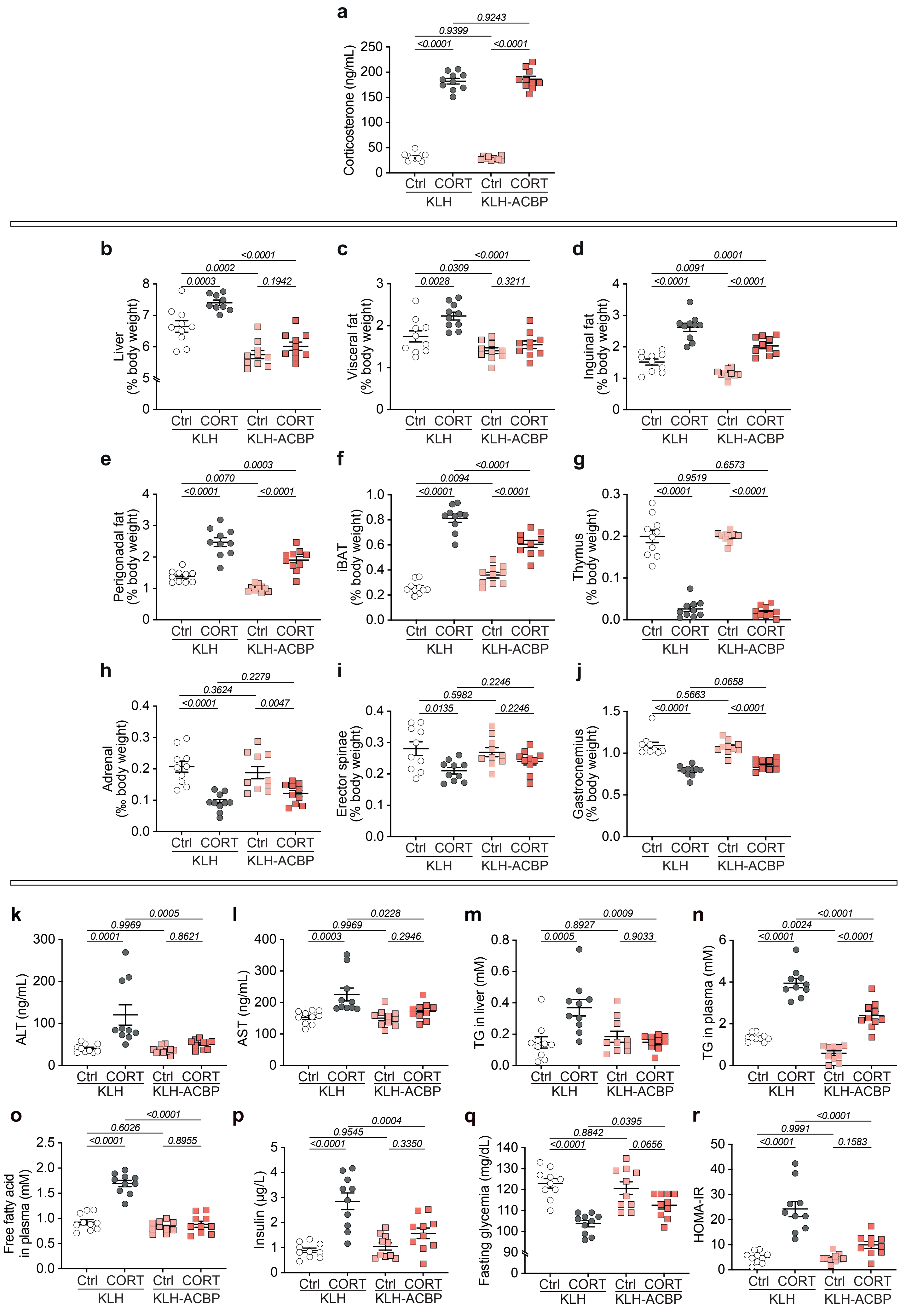

Supplemental Fig. 3

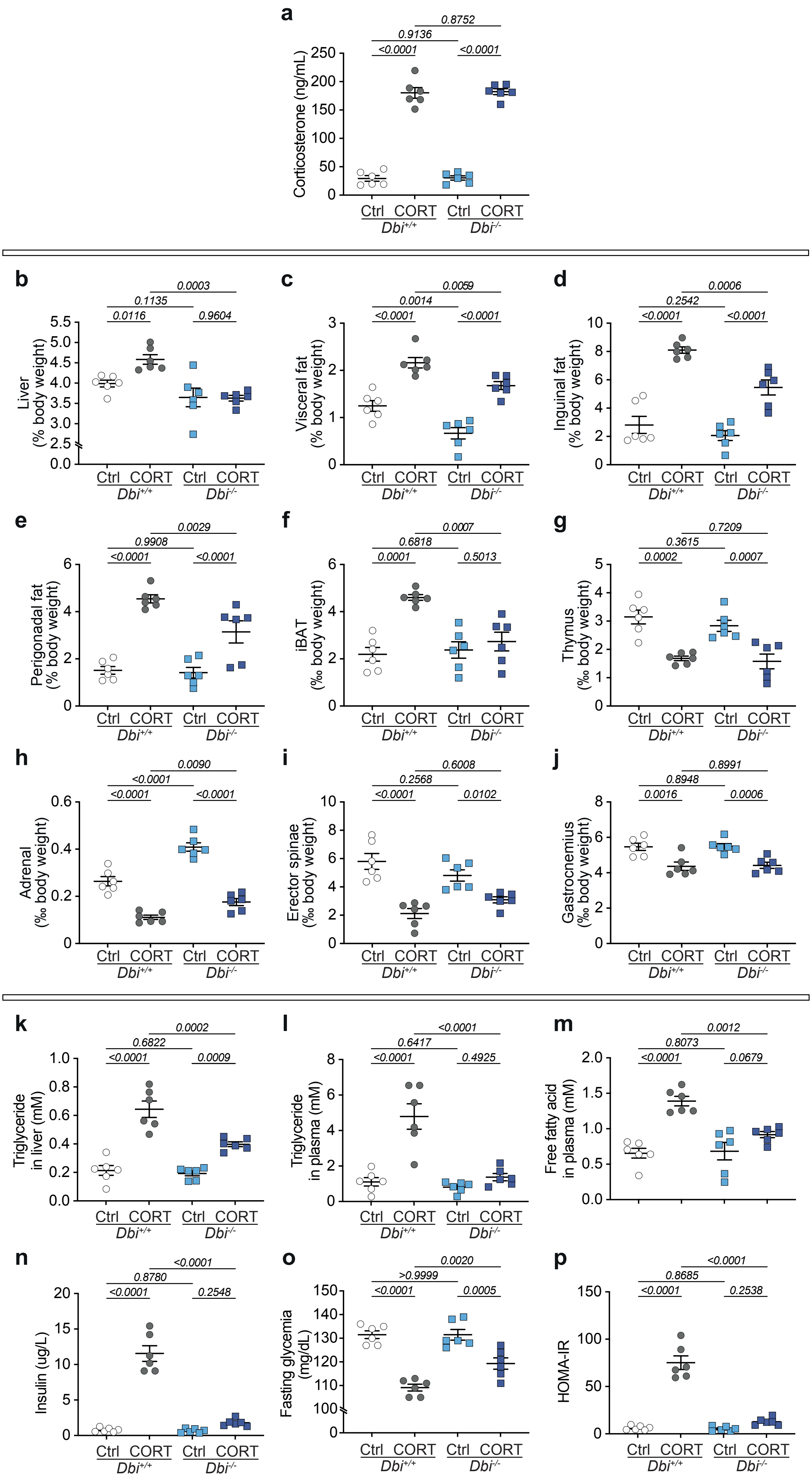

Supplemental Fig. 4

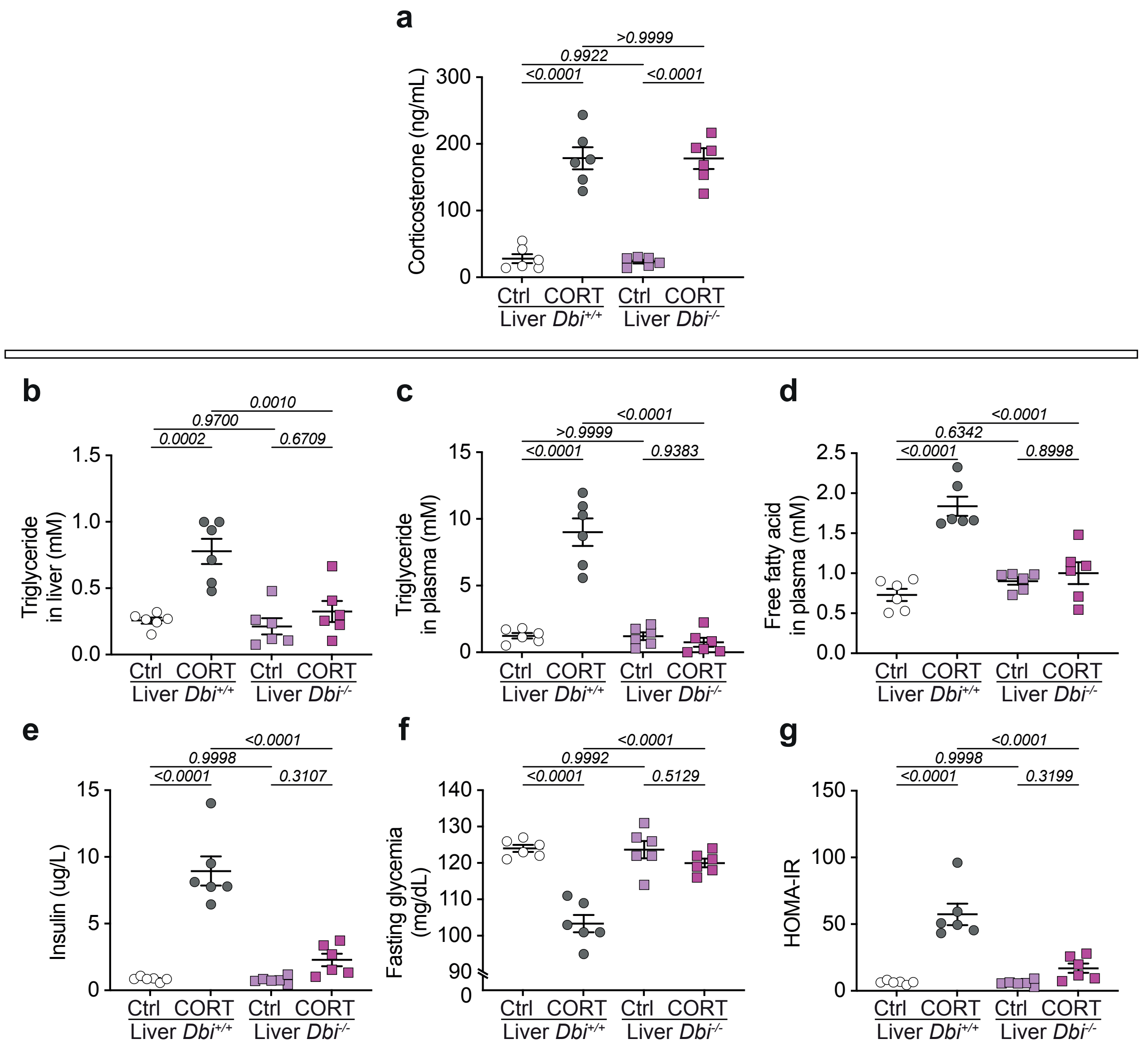

Supplemental Fig. 5

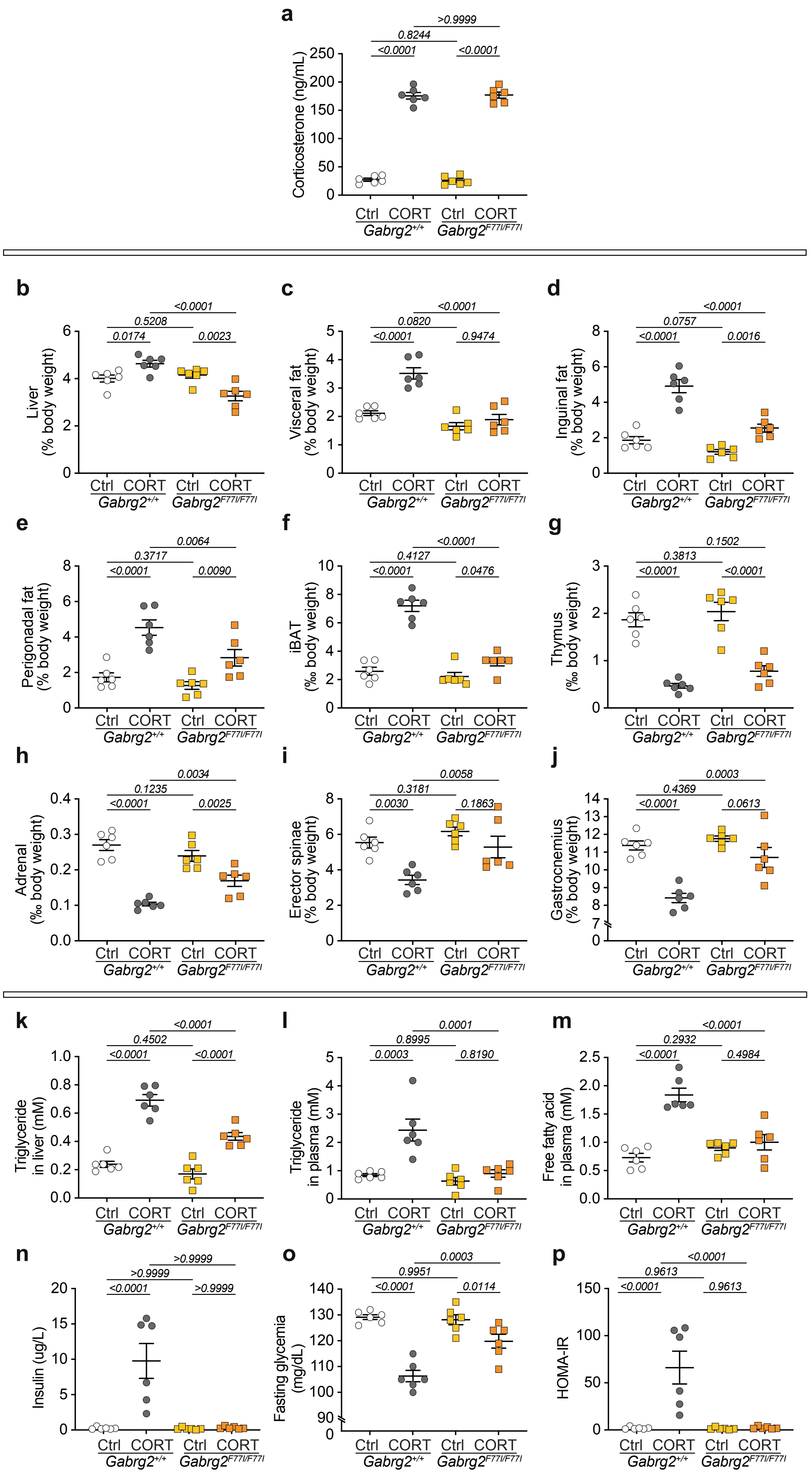

Supplemental Fig. 6

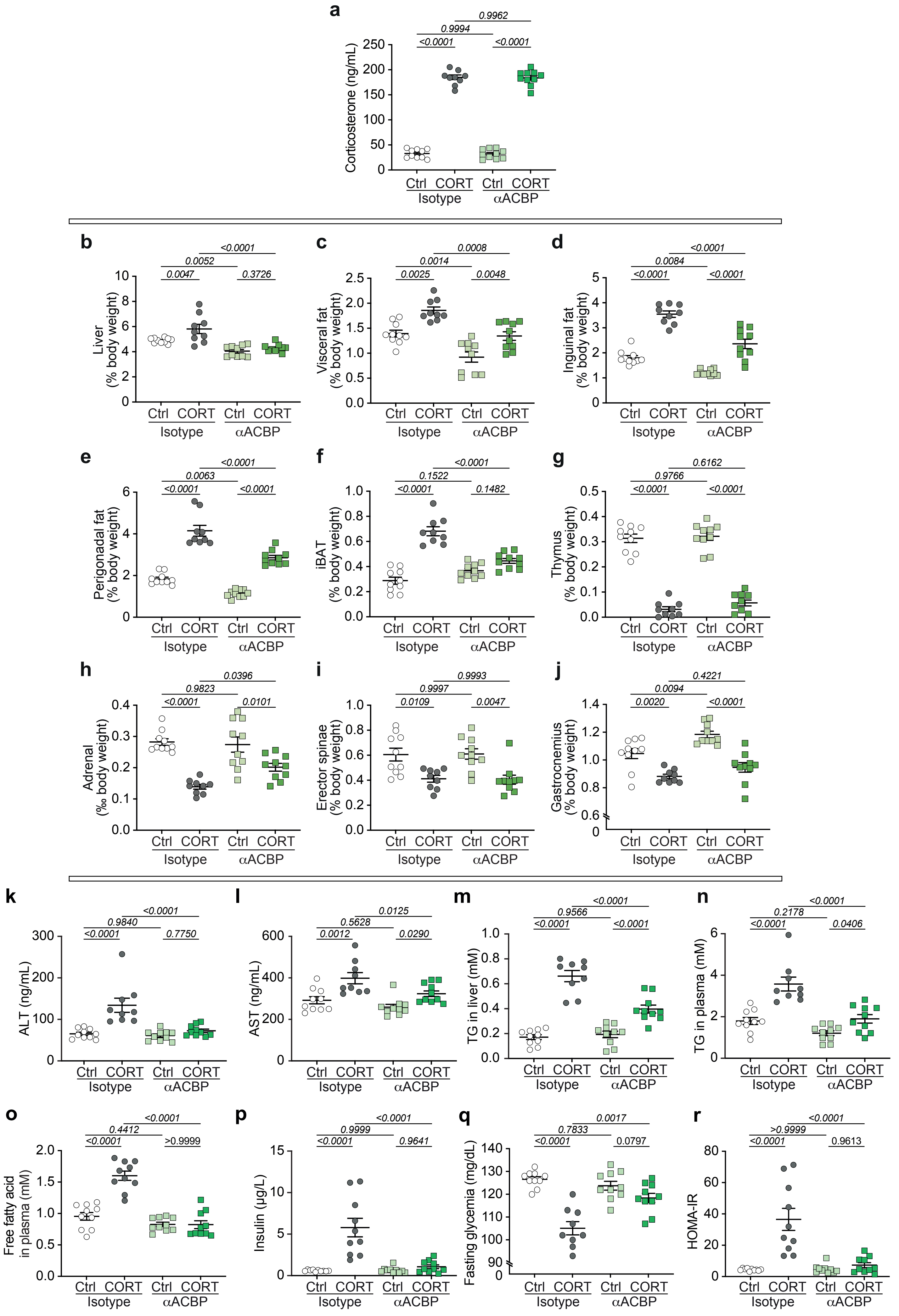

Supplemental Fig. 7

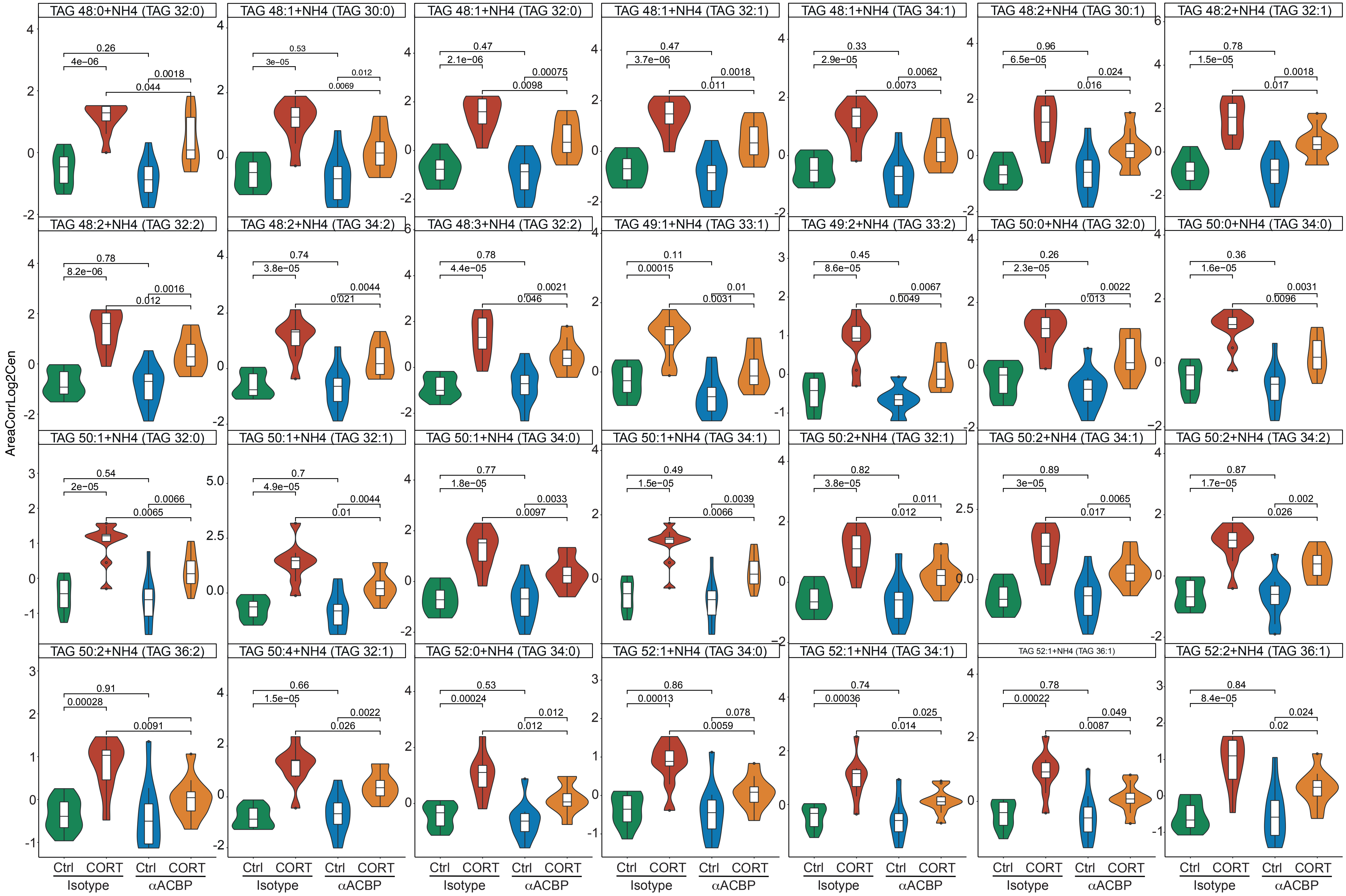

Supplemental Fig. 8

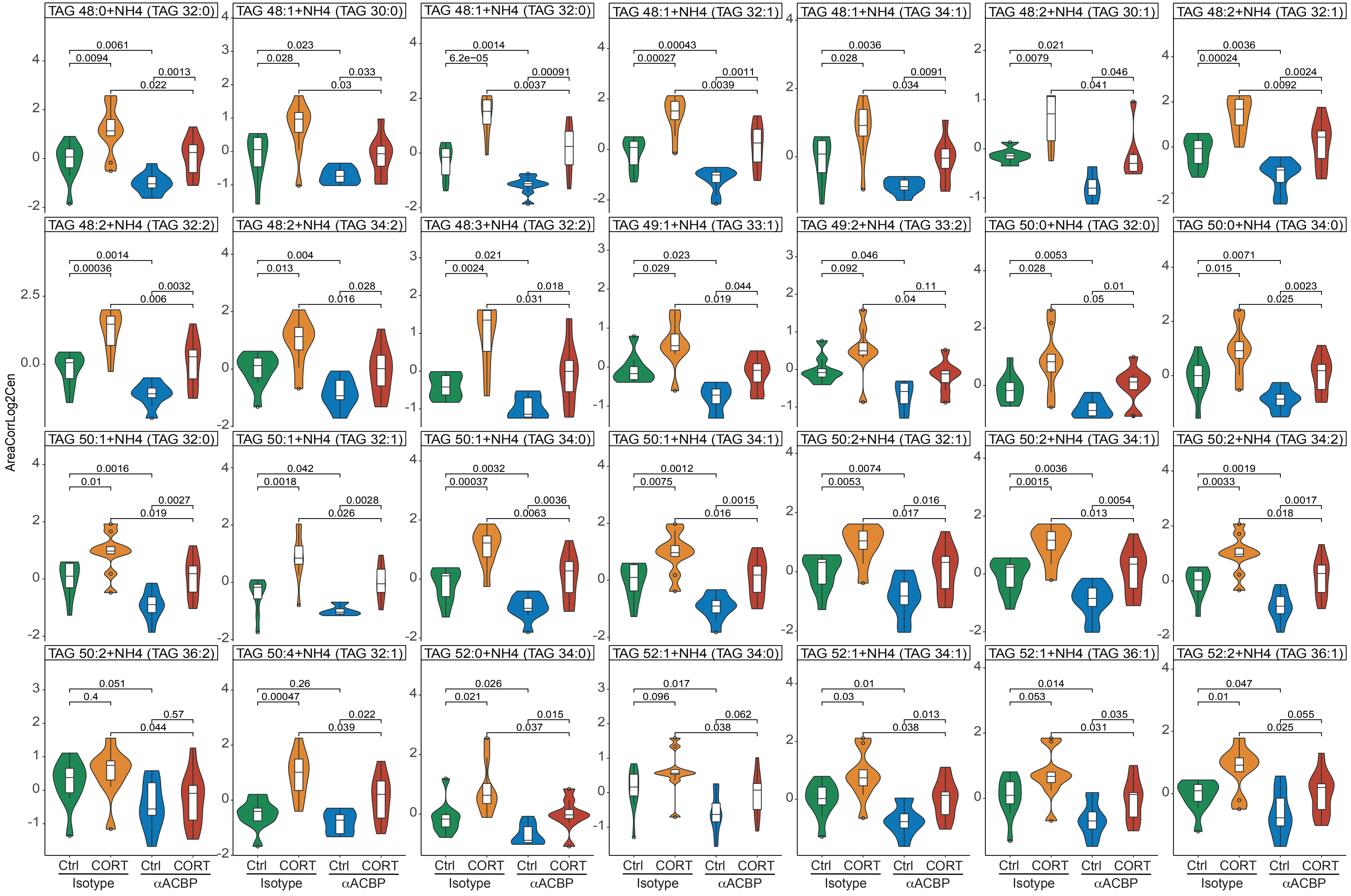

Supplemental Fig. 9

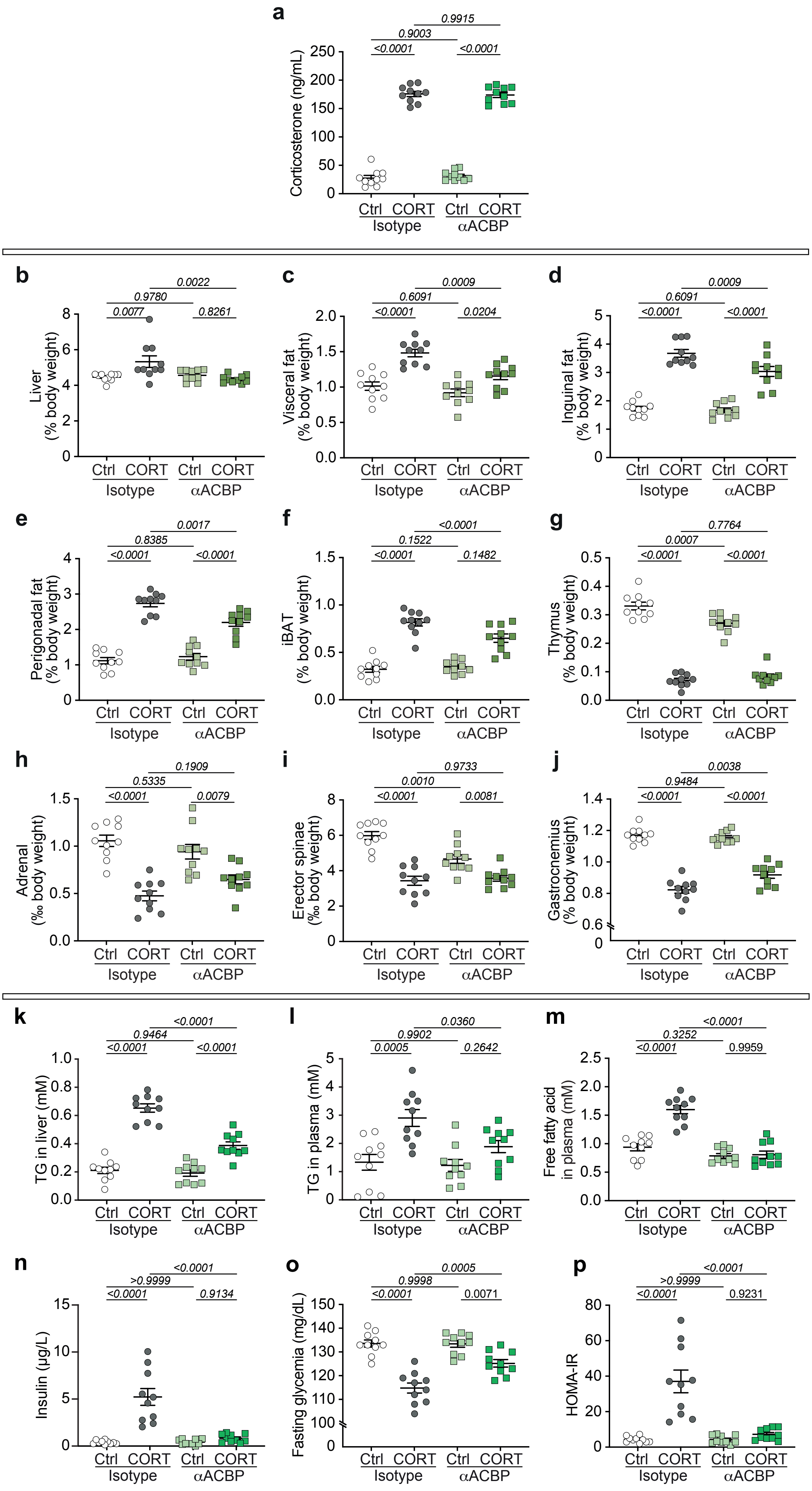

Supplemental Fig. 10

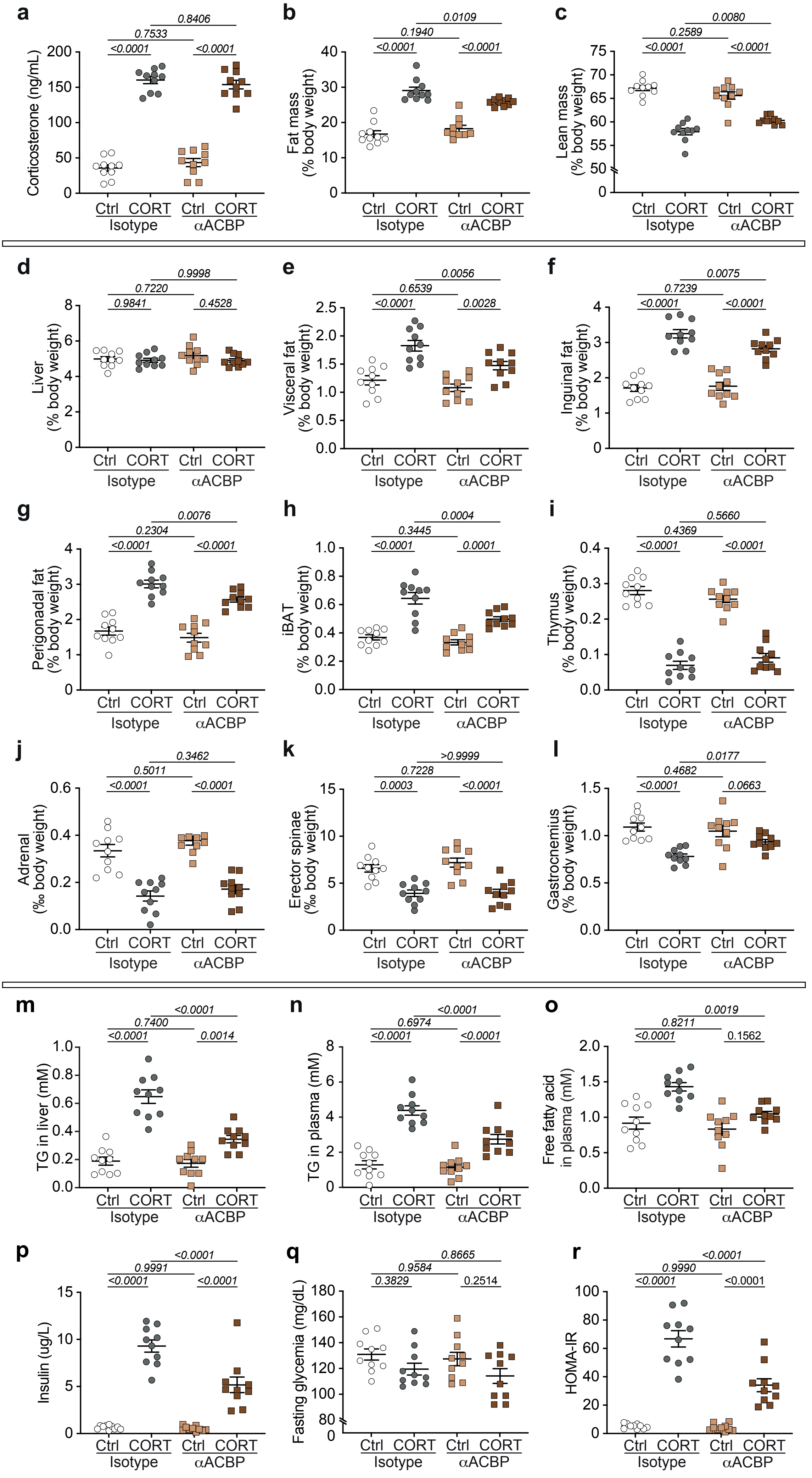

Supplemental Fig. 11

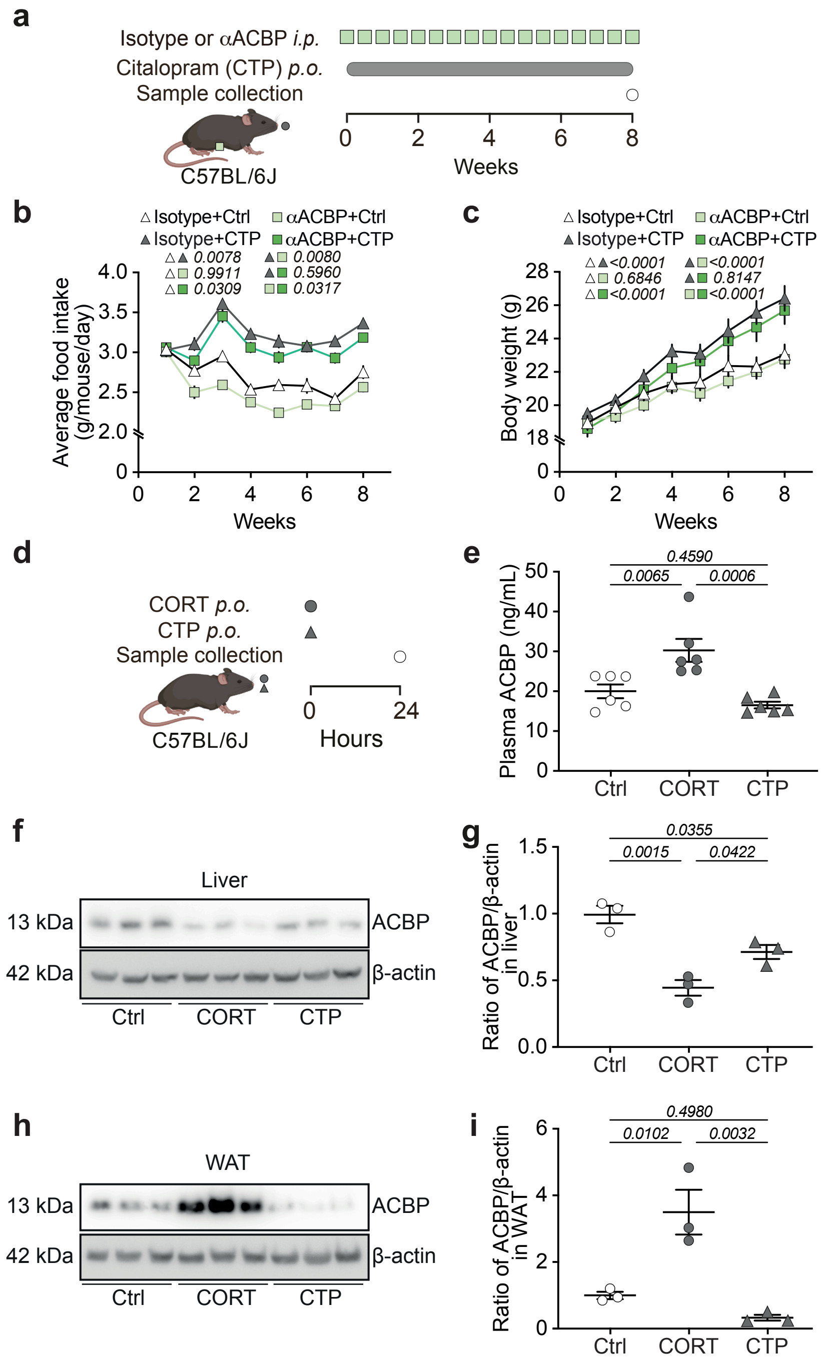

Supplemental Fig. 12

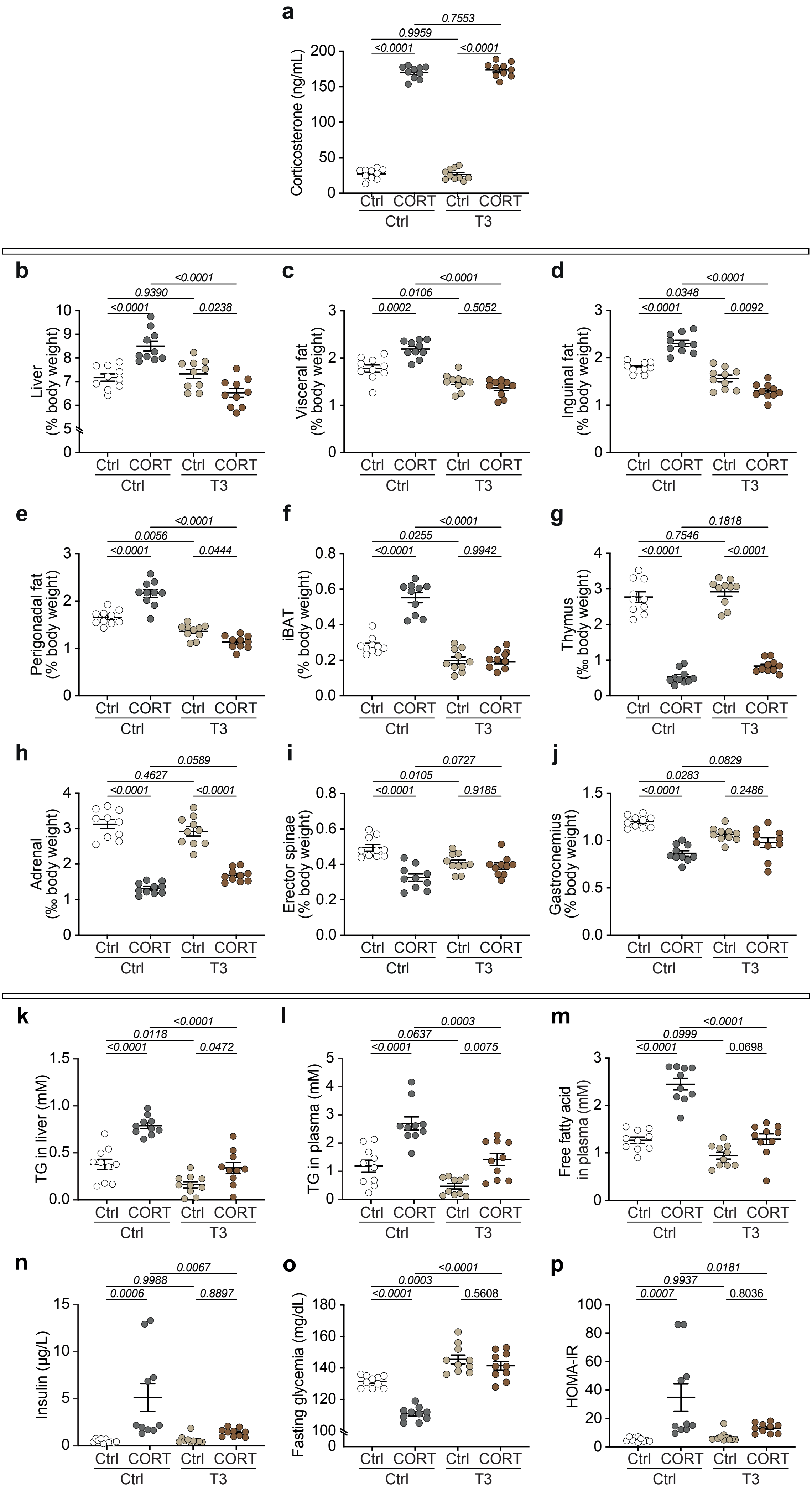

Supplemental Fig. 13

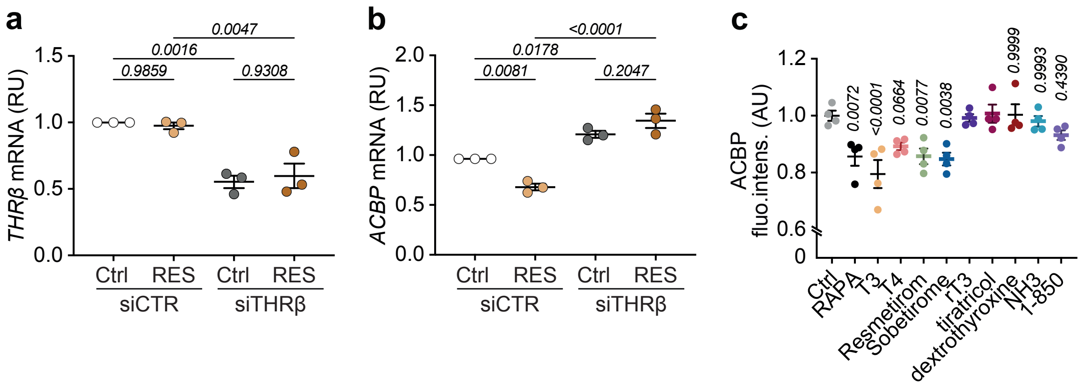

Supplemental Fig. 14

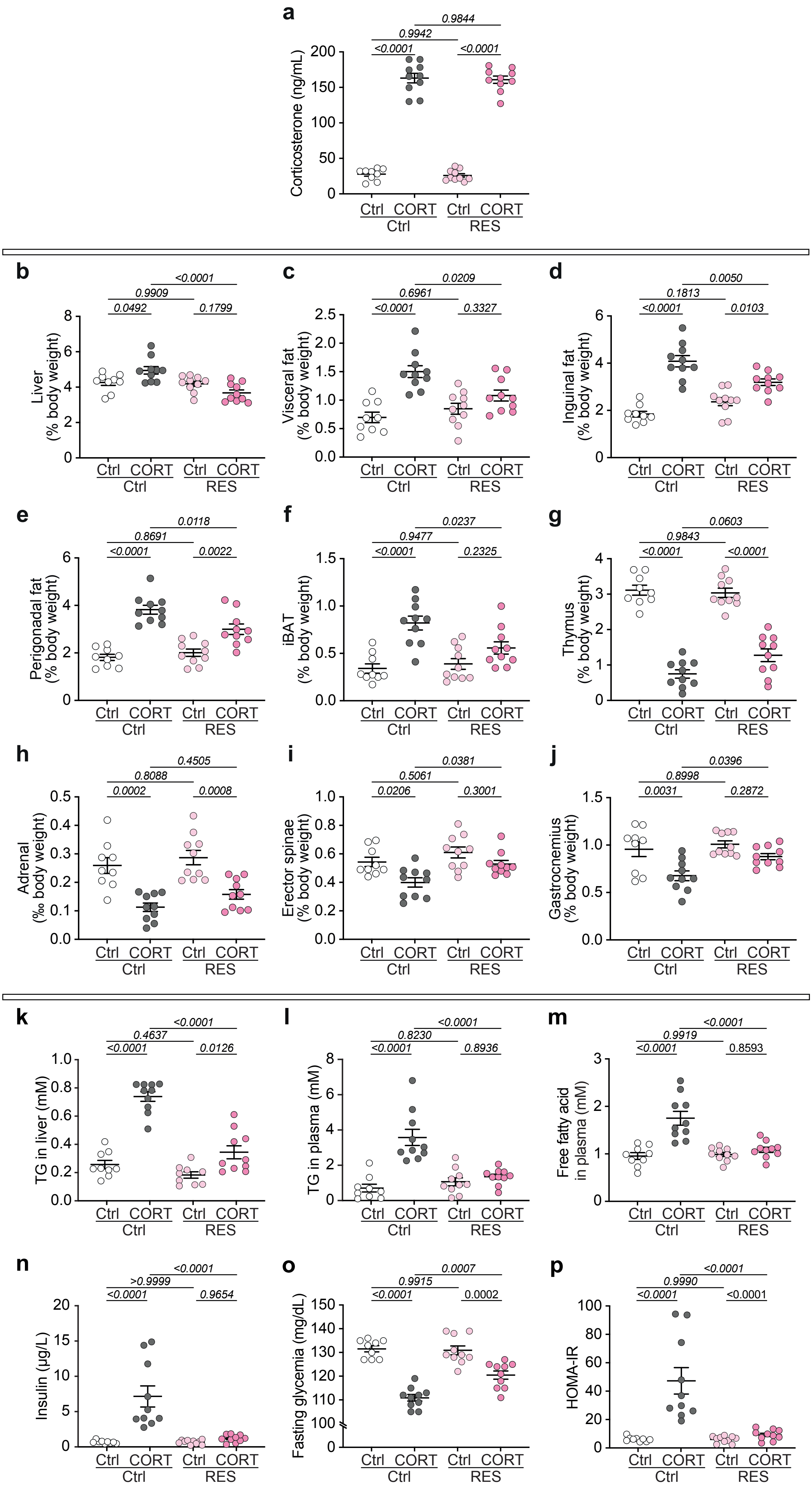

Supplemental Fig. 15

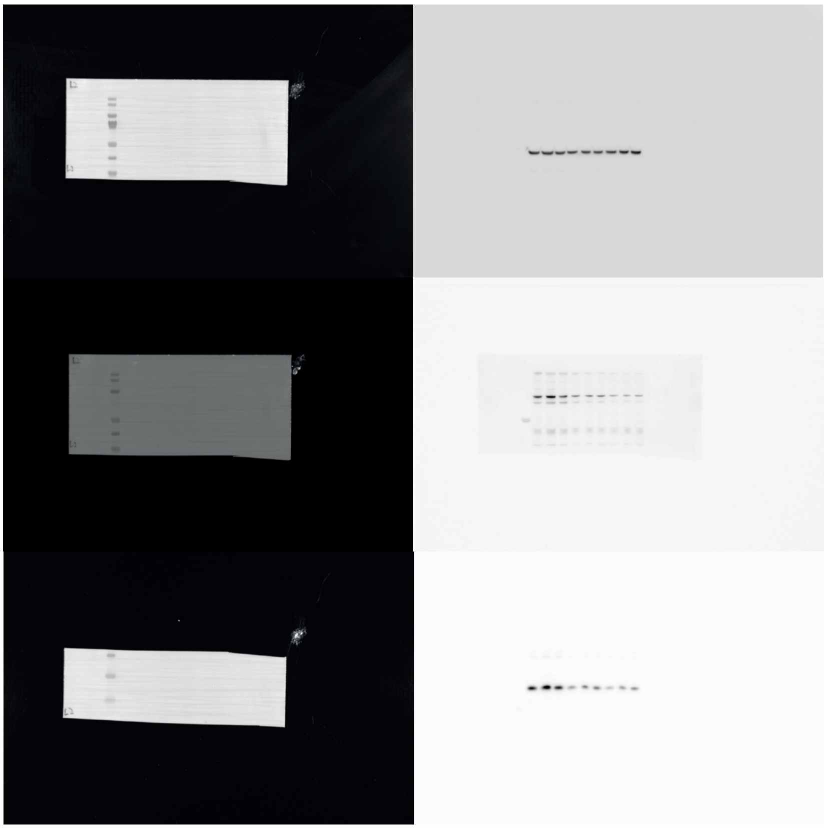

β-actin

SQSTM1/p62

ACBP/DBI

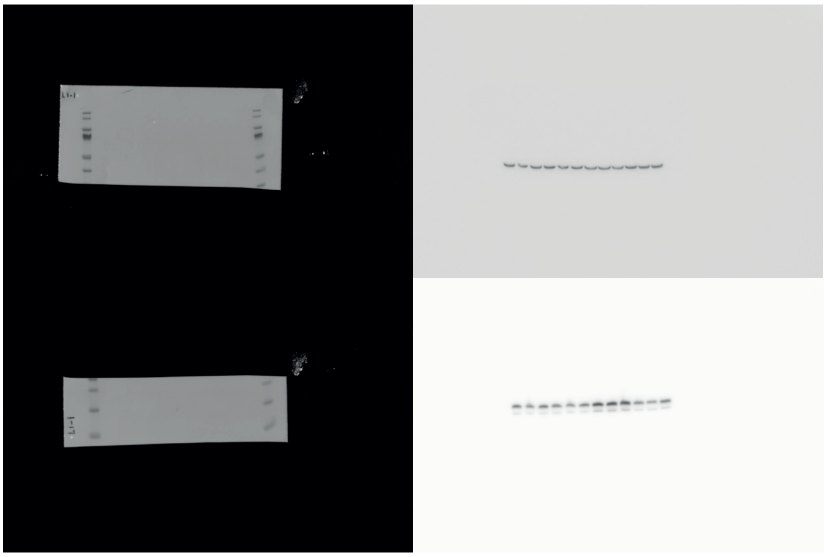

β-actin

LC3II//LC3I

Supplemental Fig.2E

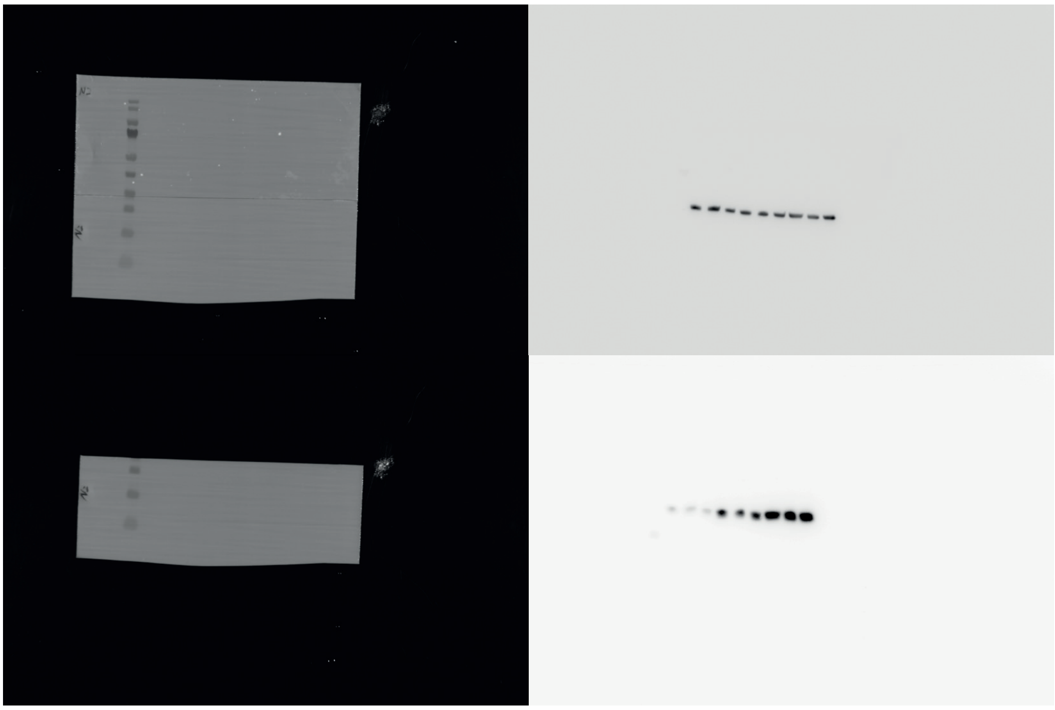

β-actin

ACBP/DBI

Supplemental Fig.2I

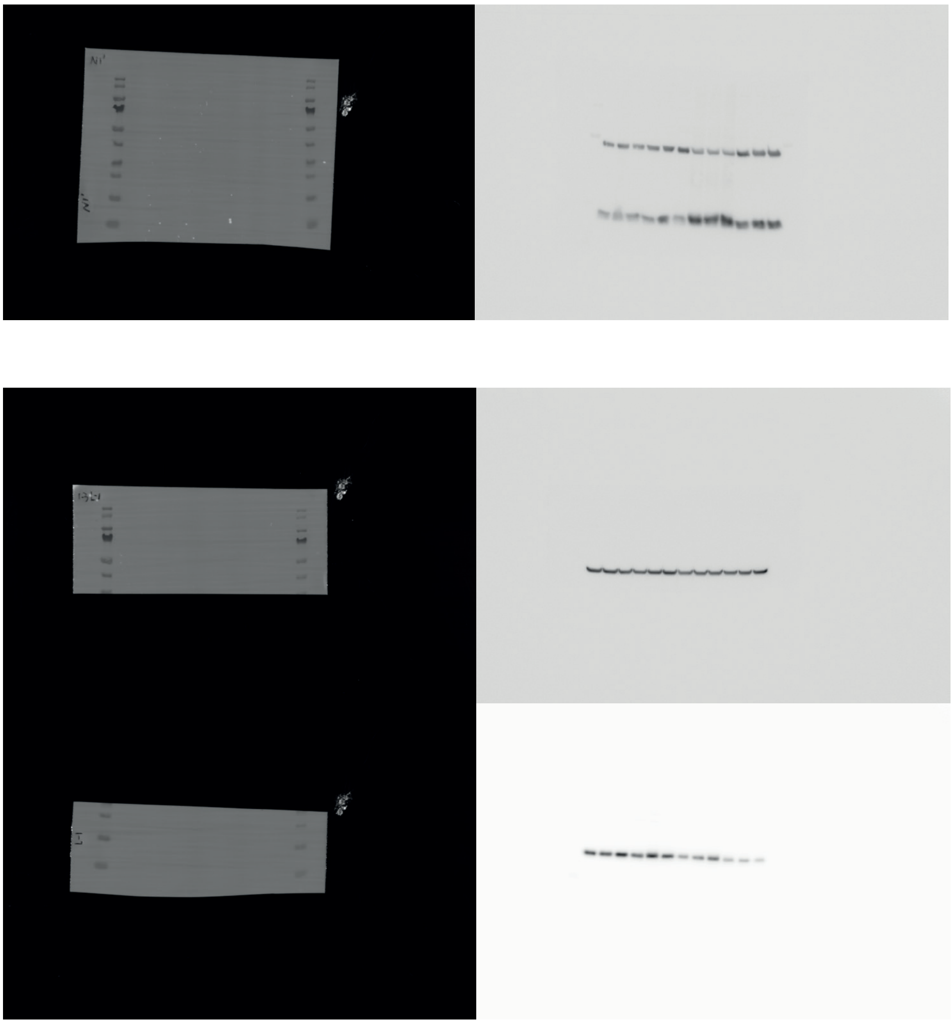

β-actin

ACBP/DBI

β-actin

ACBP/DBI

Supplemental Fig.2L

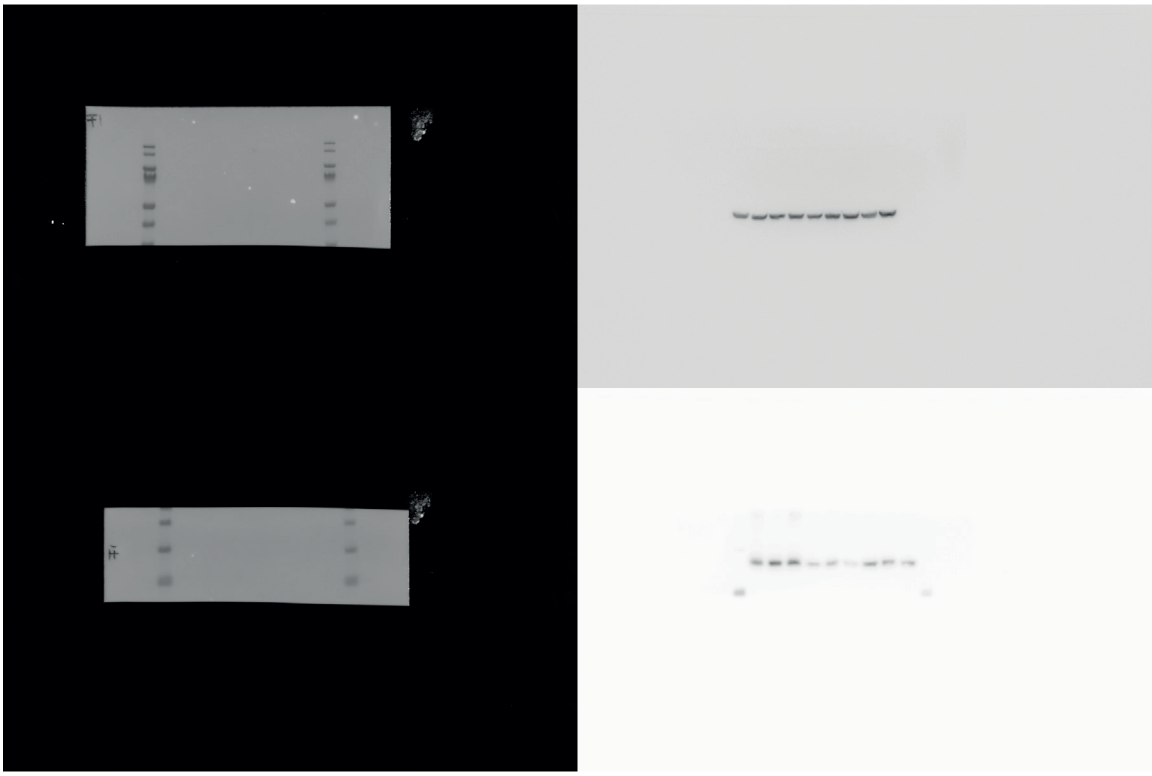

β-actin

ACBP/DBI

Supplemental Fig.12F

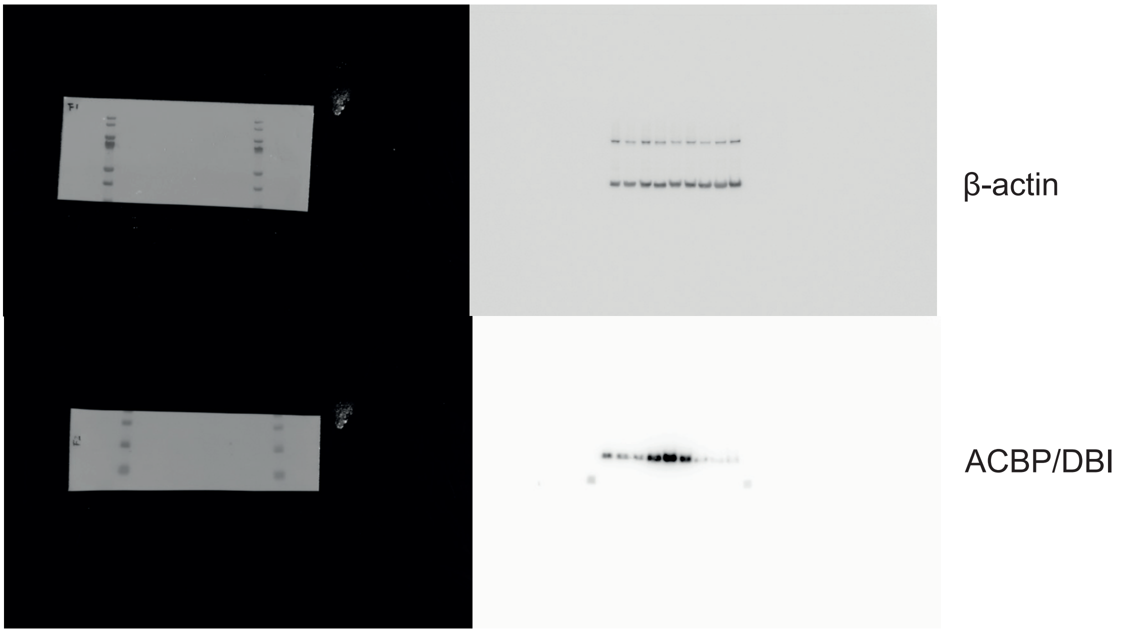

Supplemental Fig.12H
