# Supplementary material for: Pathogenic role of acyl coenzyme A binding protein (ACBP) in Cushing’s syndrome
Source: Nat Metab. 2024 Nov 22;6(12):2281–99. doi: 10.1038/s42255-024-01170-0 (PMC11659162; doi:10.1038/s42255-024-01170-0)
Supplement: Supplementary file 2 — Reporting Summary [file 42255_2024_1170_MOESM2_ESM.pdf]

nature portfolio

Corresponding author(s): Isabelle Martins, Oliver Kepp and Guido Kroemer

Last updated by author(s): Oct 4, 2024

Reporting Summary

Nature Portfolio wishes to improve the reproducibility of the work that we publish. This form provides structure for consistency and transparency in reporting. For further information on Nature Portfolio policies, see our Editorial Policies and the Editorial Policy Checklist.

Statistics

For all statistical analyses, confirm that the following items are present in the figure legend, table legend, main text, or Methods section.

n/a	Confirmed	
☐	☒	The exact sample size (*n*) for each experimental group/condition, given as a discrete number and unit of measurement
☐	☒	A statement on whether measurements were taken from distinct samples or whether the same sample was measured repeatedly
☐	☒	The statistical test(s) used AND whether they are one- or two-sided<br>*Only common tests should be described solely by name; describe more complex techniques in the Methods section.*
☐	☒	A description of all covariates tested
☐	☒	A description of any assumptions or corrections, such as tests of normality and adjustment for multiple comparisons
☐	☒	A full description of the statistical parameters including central tendency (e.g. means) or other basic estimates (e.g. regression coefficient) AND variation (e.g. standard deviation) or associated estimates of uncertainty (e.g. confidence intervals)
☐	☒	For null hypothesis testing, the test statistic (e.g. *F*, *t*, *r*) with confidence intervals, effect sizes, degrees of freedom and *P* value noted<br>*Give P values as exact values whenever suitable.*
☒	☐	For Bayesian analysis, information on the choice of priors and Markov chain Monte Carlo settings
☒	☐	For hierarchical and complex designs, identification of the appropriate level for tests and full reporting of outcomes
☐	☒	Estimates of effect sizes (e.g. Cohen's *d*, Pearson's *r*), indicating how they were calculated

Our web collection on statistics for biologists contains articles on many of the points above.

Software and code

Policy information about availability of computer code

Data collection	MetaXpress 6.7.290; QuPath v0.4.2; QuantStudio v1.5.2; Optima control v2.20R2; ImageQuant LAS4000 Version 1.2; Bruker minispec plus
Data analysis	ImageJ2 Version 2.9.0/1.53t; QuantStudio v1.5.2; GraphPad Prism 9.4.0; R software Version 4.3.1; R-packages, including clusterProfiler (v4.8.2)41; tidyverse (2.0.0); ggplot (3.4.4); forcats (1.0.0); biomaRt(2.56.1); stringr(1.5.1); org.Mm.eg.db(3.17.0); GRMeta(1.03), tumGrowth, MetaxpR (0.6.1),EBImage (4.42.0)

For manuscripts utilizing custom algorithms or software that are central to the research but not yet described in published literature, software must be made available to editors and reviewers. We strongly encourage code deposition in a community repository (e.g. GitHub). See the Nature Portfolio guidelines for submitting code & software for further information.

Data

Policy information about availability of data

All manuscripts must include a data availability statement. This statement should provide the following information, where applicable:

- Accession codes, unique identifiers, or web links for publicly available datasets
- A description of any restrictions on data availability
- For clinical datasets or third party data, please ensure that the statement adheres to our policy

The datasets generated during and/or analyzed during the current study have been annexed. RNA sequencing data is available at NCBI GEO database under the accession number GSE248672.

Research involving human participants, their data, or biological material

Policy information about studies with human participants or human data. See also policy information about sex, gender (identity/presentation), and sexual orientation and race, ethnicity and racism.

Reporting on sex and gender	Cohort I in total 92 participant; from which the control group entailed 21 female and 18 male participant and the treatment group had 31 female and 22 male participants.<br>Cohort II in total 24 participant; from which the remission group (13) entailed 12 female and 1 male participant and the active disease (11) group had 7 female and 4 male participants.
Reporting on race, ethnicity, or other socially relevant groupings	Cohort I included Chinese patients.
Population characteristics	Cohort I median age is 46.8 years; range, 11-93; Cohort II median age is 56.5 years; range, 22-73
Recruitment	In cohort I patients with skin disease were included without any age, gender or self-selection bias. Three patients who developed resistance to synthetic glucocorticoids were excluded. All patients gave their written informed consent, and the study protocol was approved by the local ethics committee.<br>For Cohort II patients with ACTH-dependent Cushing syndrome were prospectively recruited from December 2014 to June 2017 in the Endocrinology and in the Neurosurgery departments of Marseille University Hospital, France. The « active » group consisted of newly diagnosed patients. The « remission » group consisted of patients in remission for at least 2 years, but no more than 6 years, regardless of their treatment modality. All the patients gave their written informed consent, and the study protocol was approved by the local ethics committee.
Ethics oversight	The study of cohort I was approved by the Institutional Review Board of Dermatology & Hospital for Skin Diseases and the Ethics Committee of Suzhou Institute of Systems Medicine, Chinese Academy of Medical Sciences & Peking Union Medical College with the approval (2021) Linkuaishen (005) and (2023) Lunshen015, respectively. The study protocol for cohort II was approved by Marseille, Comité de Protection des Personnes, Sud Méditerranée II (identification 2014-A01302-45 and 2016-A00026-45, clinical trial identification: NCT02335996 and NCT02848703).

Note that full information on the approval of the study protocol must also be provided in the manuscript.

Field-specific reporting

Please select the one below that is the best fit for your research. If you are not sure, read the appropriate sections before making your selection.

☒ Life sciences ☐ Behavioural & social sciences ☐ Ecological, evolutionary & environmental sciences

For a reference copy of the document with all sections, see nature.com/documents/nr-reporting-summary-flat.pdf

Life sciences study design

All studies must disclose on these points even when the disclosure is negative.

Sample size	The number of animals for in vivo studies was predetermined by means of the InVivoStat package for R software. For long-term experiments (5, 8, 10 weeks), the expected effect is a weight gain of at least 30% in mice. We set the alpha risk at 0.02 and the power at 0.80. Weight gain or recovery were statistically analyzed by means of "ANOVA" tests.
Data exclusions	Outlier exclusion was performed by ROUT method in GraphPad Prism.
Replication	In vitro experiments were repeat independently three times. In vivo experiment were repeated independently at least twice. The number of replicates is indicated for each experiment.
Randomization	Mice were randomized according to body weight. 4-5 tissue samples for H&E were randomly selected per group. For immunoblot analysis and face angle assessment 3 samples per group were randomly selected.
Blinding	HCS data was analyzed in a blinded fashion. In our experiment, implementing a double-blind design was challenging due to the nature of handling and observing the mice. Researchers need to directly interact with the mice during the experiment, making it impossible to ensure that all operations are blinded. Additionally, the mice exhibited noticeable differences in appearance (weight gain) and behavior, making it easy for researchers to identify the experimental and control groups. Nonetheless, we have taken measures such as random grouping and independent data evaluation to minimize potential bias.

Reporting for specific materials, systems and methods

We require information from authors about some types of materials, experimental systems and methods used in many studies. Here, indicate whether each material, system or method listed is relevant to your study. If you are not sure if a list item applies to your research, read the appropriate section before selecting a response.

Materials & experimental systems

n/a	Involved in the study	
☐	☒	Antibodies
☐	☒	Eukaryotic cell lines
☒	☐	Palaeontology and archaeology
☐	☒	Animals and other organisms
☐	☒	Clinical data
☒	☐	Dual use research of concern
☒	☐	Plants

Methods

n/a	Involved in the study	
☒	☐	ChIP-seq
☒	☐	Flow cytometry
☒	☐	MRI-based neuroimaging

Antibodies

Antibodies used	ACBP Antibody (C-9) Santa Cruz Biotechnology Mouse monoclonal IgG 1 κ, Cat. No.:sc-376853<br>Mouse ACBP/DBI antibody Abcam Rabbit Polyclonal IgG antibody, Cat. No.:ab231910<br>Recombinant Anti-LC3B antibody Abcam Rabbit monoclonal IgG antibody, Cat. No.: ab192890<br>Recombinant Anti-SQSTM1 / p62 antibody Rabbit monoclonal IgG antibody, Cat. No.: ab109012<br>Glucocorticoid Receptor (D6H2L) XP® Rabbit mAb Cell Signaling Technology Rabbit monoclonal IgG antibody, Cat. No.:12041<br>Anti-beta Actin antibody [AC-15] (HRP) Abcam HRP Mouse monoclonal [AC-15] to beta Actin, Cat. No.: ab49900<br>Human anti-ACBP/DBI capture antibody (Rabbit anti-Human, Mouse DBI Polyclonal Antibody) MyBioSource Rabbit Polyclonal IgG antibody, Cat. No.: MBS768488<br>Human anti-ACBP/DBI detection antibody (DBI / ACBD1 Antibody) Lifespan Biosciences Polyclonal antibody to ACBD1 (DBI) (aa1-87), Cat. No.:LS-C299614<br>Goat Anti-Rabbit IgG(H+L), Mouse/Human ads-HRP, SouthernBiotech, Cat. No.: 4050-05<br>Goat Anti-Mouse IgG(H+L), Human ads-HRP, SouthernBiotech, Cat. No.: 1031-05<br>InVivoMAb mouse IgG2a isotype control, Bioxcell, Cat. No.: #BE0085<br>Anti-ACBP mAb (clone 7G4a), homemade.
Validation	All antibodies were validate by the manufacturers except the homemade Anti-ACBP mAb (clone 7G4a) which was validated inhouse by ELISA and immunoblot using appropriate positive and negative controls.

Eukaryotic cell lines

Policy information about cell lines and Sex and Gender in Research

Cell line source(s)	ATCC (H4 (Cat No.: HTB-148 ™) and HepG2 (Cat No.: HB-8065™) cell lines)
Authentication	Cells were authenticated by the provider (STR analysis (intraspecies)).
Mycoplasma contamination	All cell lines were routinely tested for mycoplasma contamination and were negative.
Commonly misidentified lines<br>(See ICLAC register)	NA

Animals and other research organisms

Policy information about studies involving animals; ARRIVE guidelines recommended for reporting animal research, and Sex and Gender in Research

Laboratory animals	Mice; C57BL/6J (6-8 weeks old); C57BL/6J Gabrg2tm1Wul/J Gabra flox (7-12 weeks old); C57BL/6J Acbpfl/fl (6-10 weeks old), C57BL/6J Acbpfl/fl UBC-CRE-ERT2(6-10 weeks old),C57BL/6J Acbpfl/fl TTR-CreTam(6-10 weeks old). Mice were group-housed and subjected to a 12 h light/dark cycle, under temperature-controlled SPF conditions with food (except the pairfeeding experiment) and water ad libitum. The ambient temperature was maintained at 22 ± 2°C, and the relative humidity was kept between 40% and 60%.
Wild animals	The study did not involve wild animals
Reporting on sex	Female and male mice were used for the present manuscript
Field-collected samples	The study does not involve samples collected from the field.
Ethics oversight	All animal experimentation procedures approved by the Gustave Roussy ethics committee (project number: 2023_053_44146, 2023_011_40501 and 2024_040_50288).

Note that full information on the approval of the study protocol must also be provided in the manuscript.

Clinical data

Policy information about clinical studies

All manuscripts should comply with the ICMJE guidelines for publication of clinical research and a completed CONSORT checklist must be included with all submissions.

Clinical trial registration	*Provide the trial registration number from ClinicalTrials.gov or an equivalent agency.*
Study protocol	*Note where the full trial protocol can be accessed OR if not available, explain why.*
Data collection	*Describe the settings and locales of data collection, noting the time periods of recruitment and data collection.*
Outcomes	*Describe how you pre-defined primary and secondary outcome measures and how you assessed these measures.*

Plants

Seed stocks	*Report on the source of all seed stocks or other plant material used. If applicable, state the seed stock centre and catalogue number. If plant specimens were collected from the field, describe the collection location, date and sampling procedures.*
Novel plant genotypes	*Describe the methods by which all novel plant genotypes were produced. This includes those generated by transgenic approaches, gene editing, chemical/radiation-based mutagenesis and hybridization. For transgenic lines, describe the transformation method, the number of independent lines analyzed and the generation upon which experiments were performed. For gene-edited lines, describe the editor used, the endogenous sequence targeted for editing, the targeting guide RNA sequence (if applicable) and how the editor was applied.*
Authentication	*Describe any authentication procedures for each seed stock used or novel genotype generated. Describe any experiments used to assess the effect of a mutation and, where applicable, how potential secondary effects (e.g. second site T-DNA insertions, mosiacism, off-target gene editing) were examined.*
