# Supplementary figures and images for: Pathogenic role of acyl coenzyme A binding protein (ACBP) in Cushing’s syndrome
Source: Nat Metab. 2024 Nov 22;6(12):2281–99. doi: 10.1038/s42255-024-01170-0 (PMC11659162; doi:10.1038/s42255-024-01170-0)

i

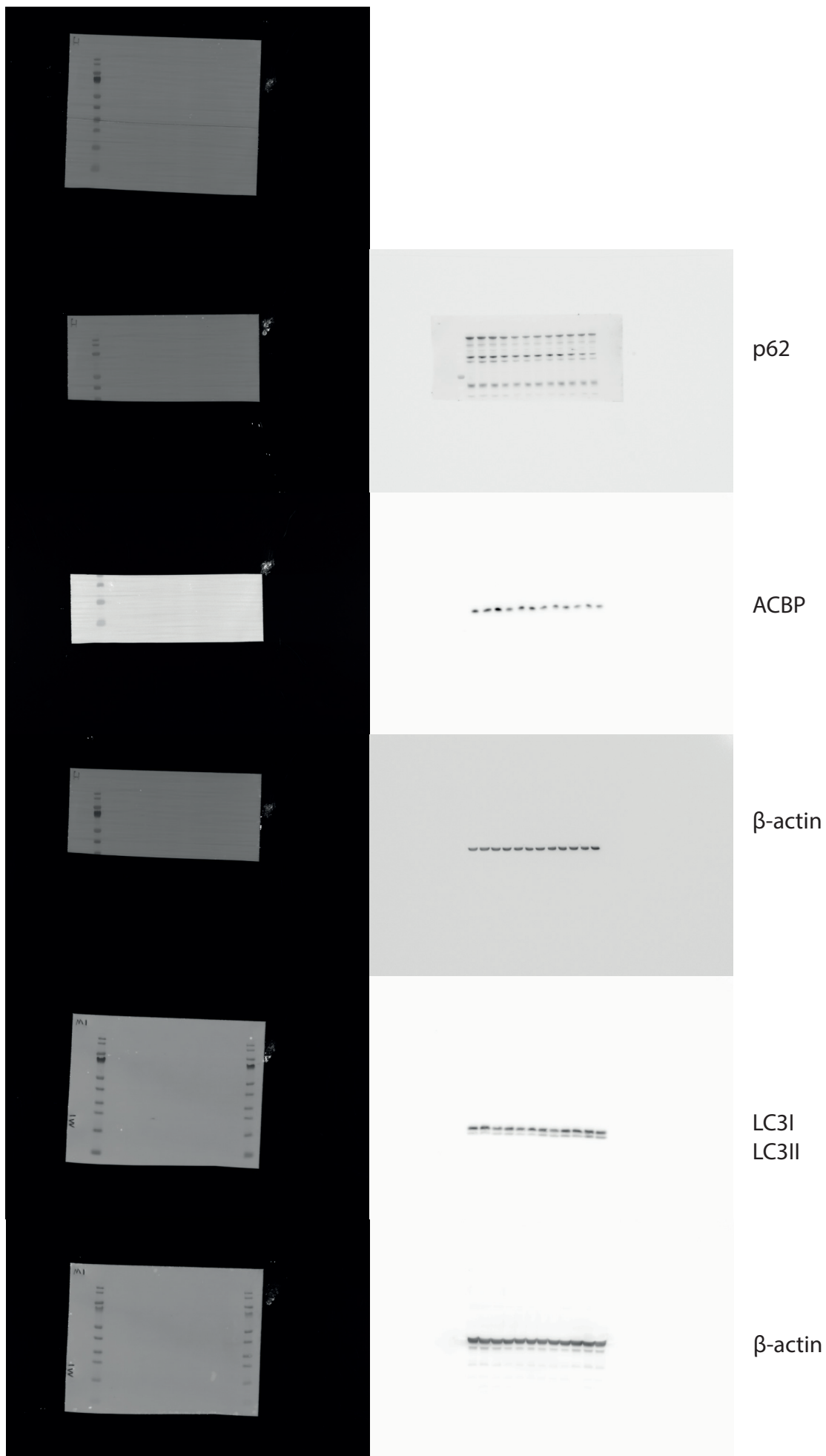

Figure 1i

Supplement: Supplementary file 7 — Unprocessed western blots. [file 42255_2024_1170_MOESM7_ESM.pdf]

a

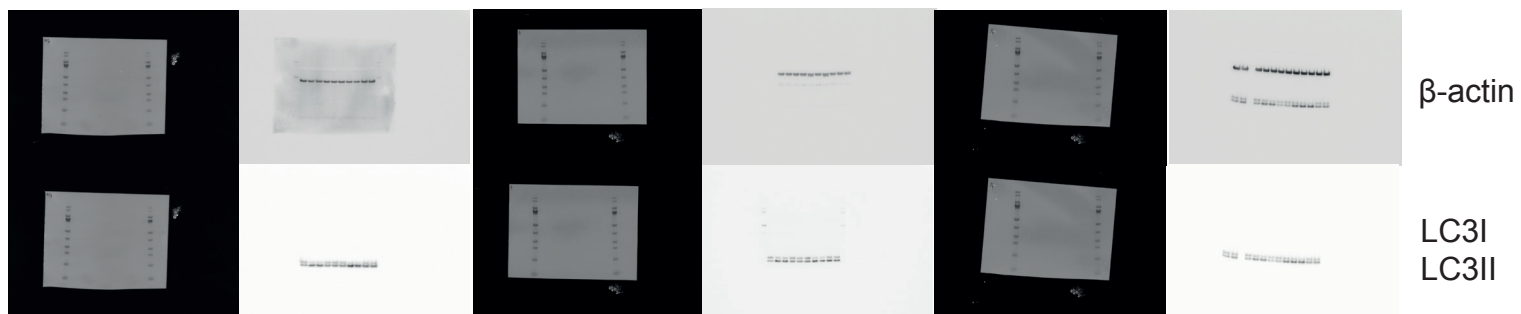

d

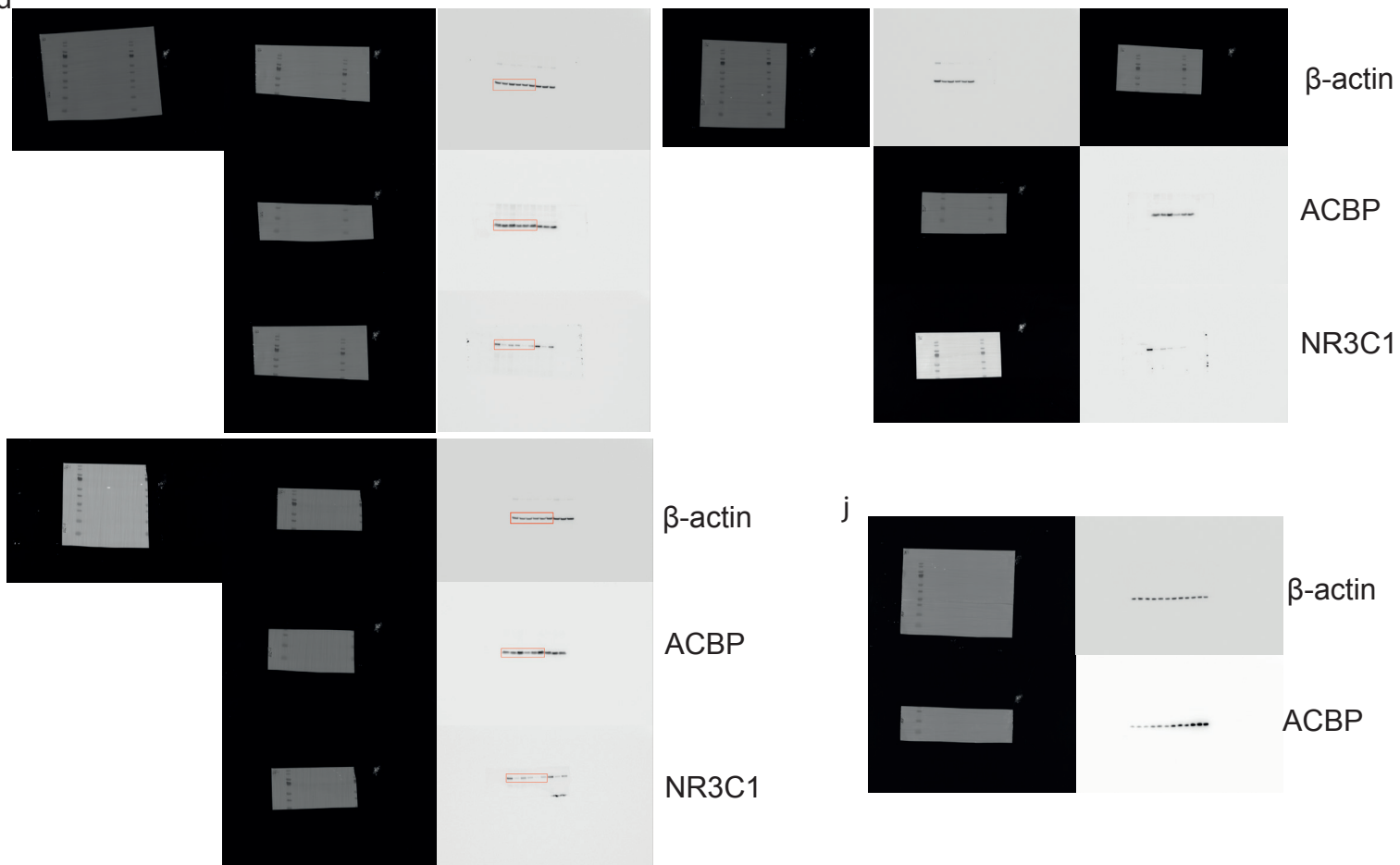

j

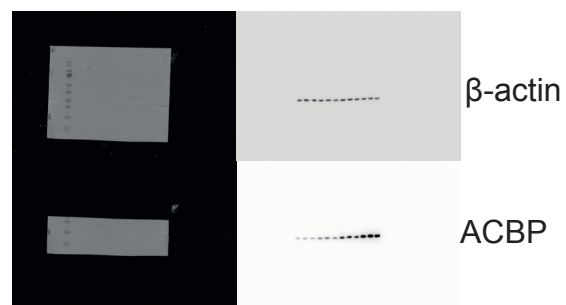

o

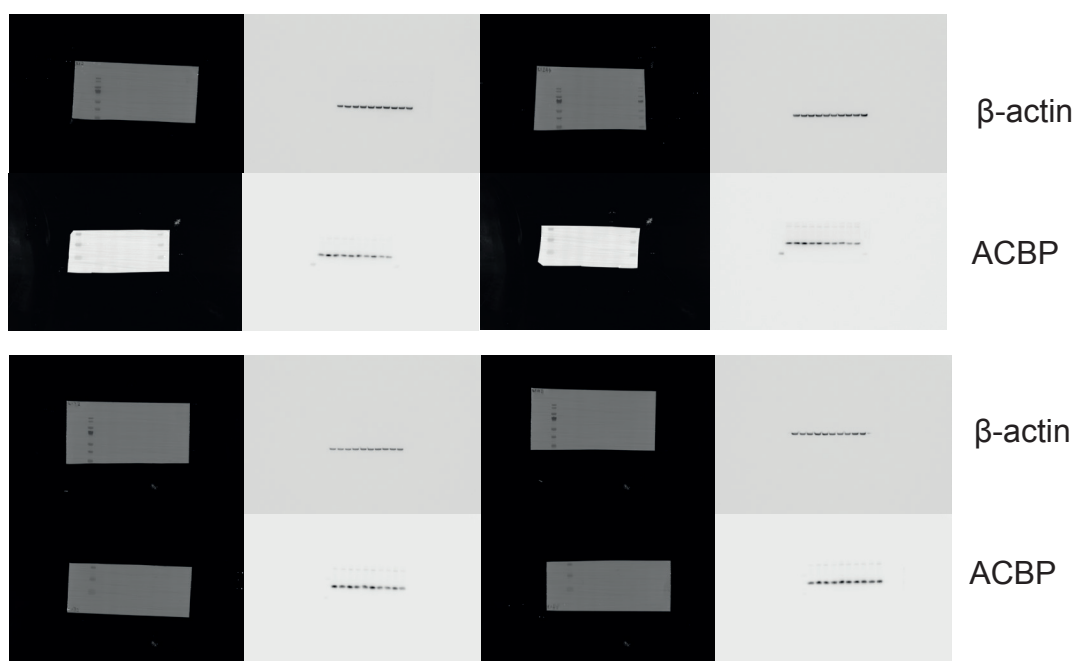

Supplement: Supplementary file 17 — Unprocessed western blots. [file 42255_2024_1170_MOESM17_ESM.pdf]

h

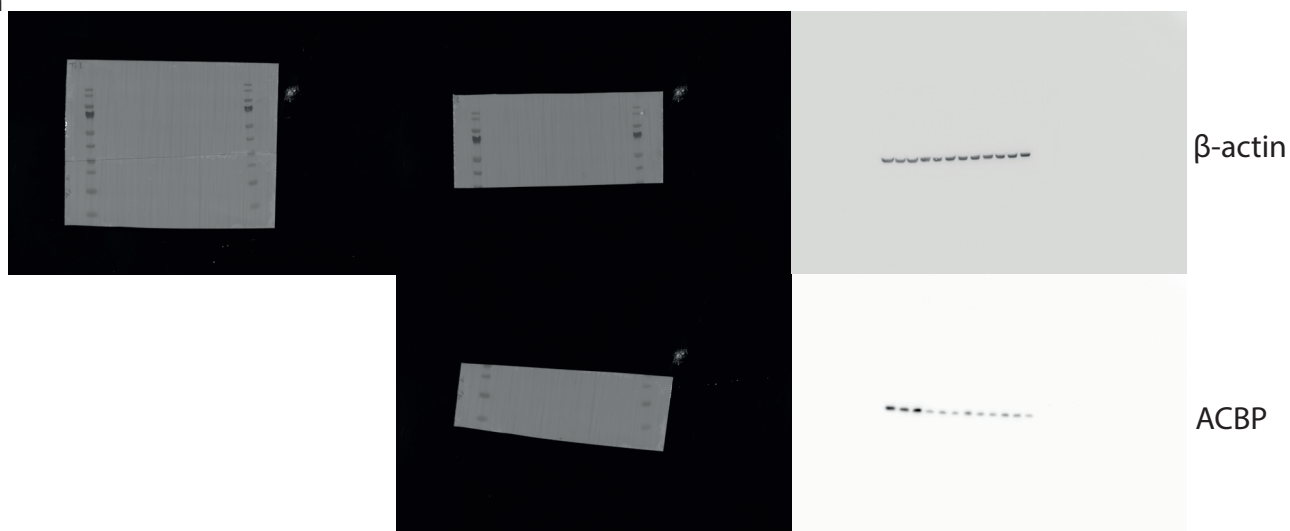

Extended Fig 2

Supplement: Supplementary file 19 — Unprocessed western blots. [file 42255_2024_1170_MOESM19_ESM.pdf]

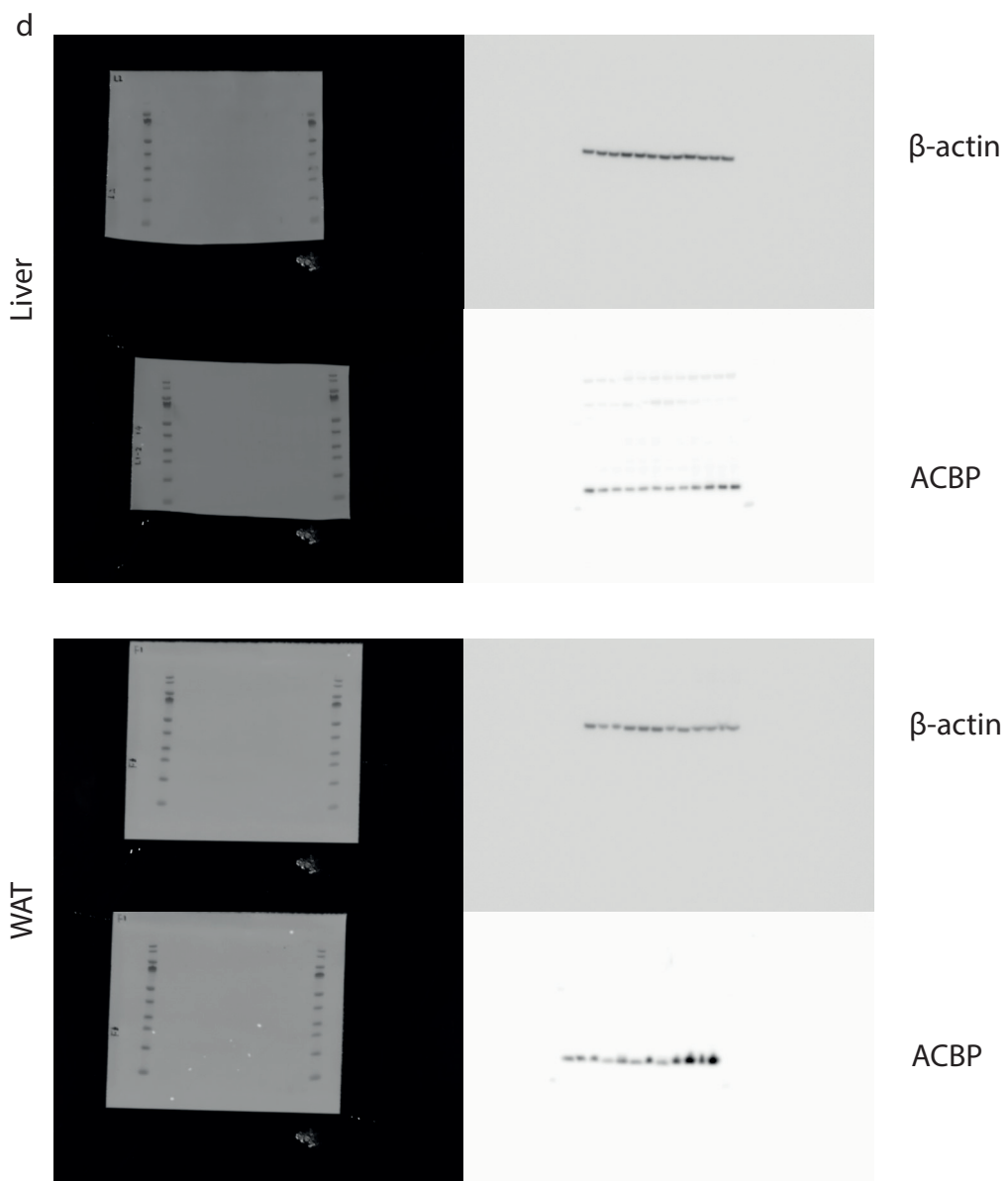

Extended Fig.9

Supplement: Supplementary file 27 — Unprocessed western blots. [file 42255_2024_1170_MOESM27_ESM.pdf]
