# Supplementary material for: Pathogenic role of acyl coenzyme A binding protein (ACBP) in Cushing’s syndrome
Source: Nat Metab. 2024 Nov 22;6(12):2281–99. doi: 10.1038/s42255-024-01170-0 (PMC11659162; doi:10.1038/s42255-024-01170-0)
Supplement: Supplementary file 9 — Unprocessed western blots. [file 42255_2024_1170_MOESM9_ESM.pdf]

c

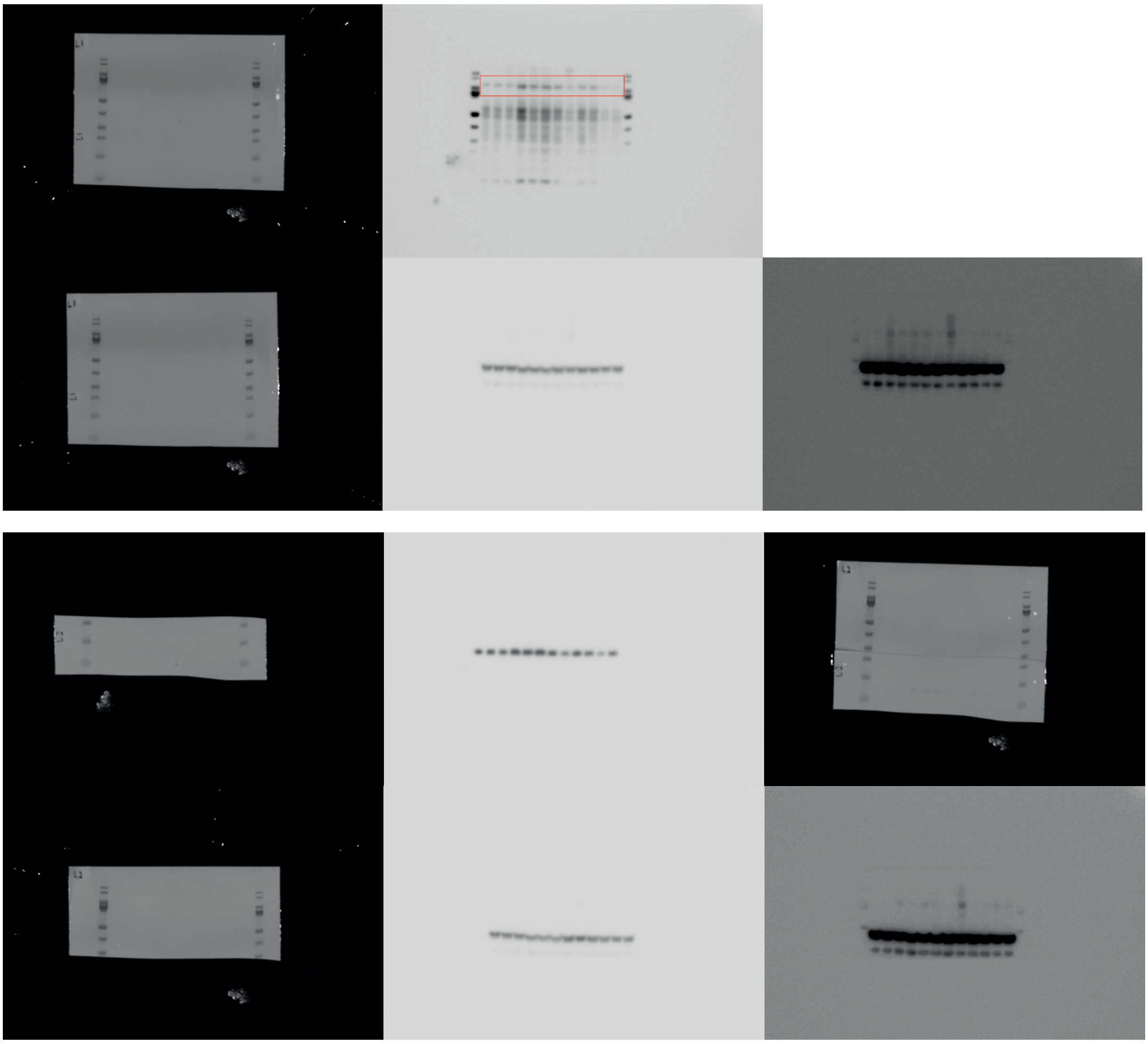

NR3C1

β-actin

ACBP

β-actin

Figure 2c
